# Significant contributions of combustion-related sources to ammonia emissions

**DOI:** 10.1038/s41467-022-35381-4

**Published:** 2022-12-13

**Authors:** Zhi-Li Chen, Wei Song, Chao-Chen Hu, Xue-Jun Liu, Guan-Yi Chen, Wendell W. Walters, Greg Michalski, Cong-Qiang Liu, David Fowler, Xue-Yan Liu

**Affiliations:** 1 School of Earth System Science, Tianjin University, Tianjin, 300072 China; 2 College of Resources and Environmental Sciences, China Agricultural University, Beijing, 100193 China; 3 School of Environmental Science and Engineering, Tianjin University, Tianjin, 300072 China; 4 Institute at Brown for Environment and Society, Brown University, 85 Waterman St, Providence, RI 02912 USA; 5 Department of Earth, Atmospheric, and Planetary Sciences, Purdue University, 550 Stadium Mall Drive, West Lafayette, IN 47907 USA; 6 Centre for Ecology and Hydrology, Bush Estate, Penicuik, Midlothian EH26 0QB United Kingdom

**Keywords:** Element cycles, Atmospheric chemistry, Geochemistry

## Abstract

Atmospheric ammonia (NH_3_) and ammonium (NH_4_^+^) can substantially influence air quality, ecosystems, and climate. NH_3_ volatilization from fertilizers and wastes (v-NH_3_) has long been assumed to be the primary NH_3_ source, but the contribution of combustion-related NH_3_ (c-NH_3_, mainly fossil fuels and biomass burning) remains unconstrained. Here, we collated nitrogen isotopes of atmospheric NH_3_ and NH_4_^+^ and established a robust method to differentiate v-NH_3_ and c-NH_3_. We found that the relative contribution of the c-NH_3_ in the total NH_3_ emissions reached up to 40 ± 21% (6.6 ± 3.4 Tg N yr^−1^), 49 ± 16% (2.8 ± 0.9 Tg N yr^−1^), and 44 ± 19% (2.8 ± 1.3 Tg N yr^−1^) in East Asia, North America, and Europe, respectively, though its fractions and amounts in these regions generally decreased over the past decades. Given its importance, c-NH_3_ emission should be considered in making emission inventories, dispersion modeling, mitigation strategies, budgeting deposition fluxes, and evaluating the ecological effects of atmospheric NH_3_ loading.

## Abstract (web summary)

By integrating nitrogen isotope systematics of ammonia emissions and transformations in the atmosphere, this study quantified the combustion-related ammonia emission and uncovered its importance for mitigating strategies of ammonia pollution.

## Introduction

Ammonia (NH_3_) is a highly water-soluble and reactive gas in the atmosphere^1^. The uptake of NH_3_ within clouds and rain and onto atmospheric aerosols causes the formation of ammonium (NH_4_^+^) within atmospheric particulates and precipitation (denoted as p-NH_4_^+^ and w-NH_4_^+^, respectively)^1–3^. Over the last century, urbanization and industrial and agricultural intensification have greatly increased NH_3_ production and have led to a continuous increase in the NH_3_ emission amounts and deposition fluxes^4–8^. Globally, the NH_3_ emission has increased from 20.6 Tg N yr^−1^ in 1860 to 58.2 Tg N yr^−1^ in 1993 and may double to 118.0 Tg N yr^−1^ by 2050^4^. Since the 1950s, East Asia, North America, and Europe have been three regions of high NH_3_ emissions^9,10^, with total emission amounts of 10.7 ± 0.4, 3.7 ± 0.2, and 3.5 ± 0.3 Tg N yr^−1^ during 2000–2015, respectively, according to emission inventory data (Supplementary Table 1). As a result, atmospheric NH_3_ concentrations (averaging 2.9 ± 2.4, 1.4 ± 1.8, 1.2 ± 1.3 μg m^−3^, respectively^11^) and NH_x_ deposition (the sum of NH_3_, p-NH_4_^+^, and w-NH_4_^+^) (averaging 12.0, 4.7, and 6.9 Tg N yr^−1^, respectively^12,13^) in the above three regions also remain high. In human-disturbed areas, excessive NH_3_ has promoted secondary aerosol production and air pollution^2–4^. Elevated NH_x_ concentrations and deposition have caused negative impacts on ecosystem structure and function (e.g., biodiversity declines, soil acidification, water eutrophication^2,3,14^) and huge economic loss^15–17^.

There are two major groups of atmospheric NH_3_ emission sources. One is NH_3_ volatilization from NH_4_^+^-containing substrates (mainly fertilized and natural soils, animal wastes, and natural and N-polluted water) (denoted as v-NH_3_)^2,9,18,19^. The dissolved NH_3_ volatilizes from liquid-phase substrates containing NH_4_^+^ at favorable pH, temperature, and pressure conditions^20^. The other is NH_3_ emission from combustion-related sources (mainly coal combustion, vehicle exhausts, and biomass burning) (denoted as c-NH_3_)^21–24^. NH_3_ would be released from industrial coal combustion, heavy-duty and light-duty diesel vehicles equipped with selective catalytic or non-catalytic reduction systems because of excessive urea/NH_3_ used for the catalytic degradation of nitrogen oxides (NO_x_)^25,26^. It is also produced via steam reforming from hydrocarbons^27^ and or catalytic reaction of nitric oxide with molecular hydrogen^28^ in light and medium-dust gasoline vehicles equipped with three-way catalytic converters, relating to the catalyst temperatures and air-to-fuel ratios^29^. The biomass N, typically as amides (R-(C = O)-NH-R’) and amines (R-NH_2_), can produce NH_3_ under poor mixing conditions during biomass burning^30^. So far, there have been direct observations on emission factors of various v-NH_3_ sources to budget the v-NH_3_ emission amount (Supplementary Table 1; Supplementary Fig. 1a). According to statistical emission inventories, the v-NH_3_ is the dominant source of regional NH_3_ emissions, accounting for 94 ± 1%, 90 ± 1%, and 95 ± 1% of the total emission in East Asia during 2001–2015, North America during 1970–2019, and Europe during 1970–2018, respectively (Supplementary Fig. 1b). In contrast, it has long been difficult to estimate the c-NH_3_ emission because of limited data on emission factors of c-NH_3_ sources and uncertainties associated with the amount of combusted materials, especially biomasses^22,23^.

However, evidence from laboratory simulations, in-situ observations, satellite observations, and emission inventories points to an underestimation of c-NH_3_ emissions relative to previous assessments (summarized in Supplementary Table 2). First, laboratory simulations found that biomass burning and light-duty diesel vehicles equipped with selective catalytic reduction are important NH_3_ sources and have been overlooked (Supplementary Table 2). Second, ground observations found that ambient NH_3_ concentrations at sites impacted by biomass burning, traffic pollution, industrial pollution, or urban pollution were enhanced by a factor of 1.4–20 compared to unpolluted sites (Supplementary Table 2). Third, satellite observations revealed that the NH_3_ from biomass burning controlled seasonal variations of surface NH_3_ concentrations in the southern and high-emission regions of China, the USA, and Europe in the northern hemisphere (Supplementary Table 2). Spatially, satellite observations also identified 13 hotspots of urban NH_3_ pollution and 266 hotspots of industrial NH_3_ pollution from coal combustion and coal-related industries (Supplementary Table 2). Fourth, according to emission inventories, the total c-NH_3_ emissions from transportation (1.3 Tg N yr^−1^)^23^, biomass burning (8.2 Tg N yr^−1^)^31^, and other combustion sources (6.3 Tg N yr^−1^)^10^ has reached up to 15.9 Tg N yr^−1^, which accounts for 30% of the global NH_3_ emission (54.3 Tg N yr^−1^). Additionally, isotopic evidence demonstrated that c-NH_3_ had reached 29–62% in NH_3_ of the ambient atmosphere^32–34^ and 45–90% of the NH_4_^+^ deposition in cities of China and the USA^35,36^. All the above evidence suggests that the relative importance and amount of regional c-NH_3_ emissions are still open questions and should be re-evaluated.

**Fig. 1:**
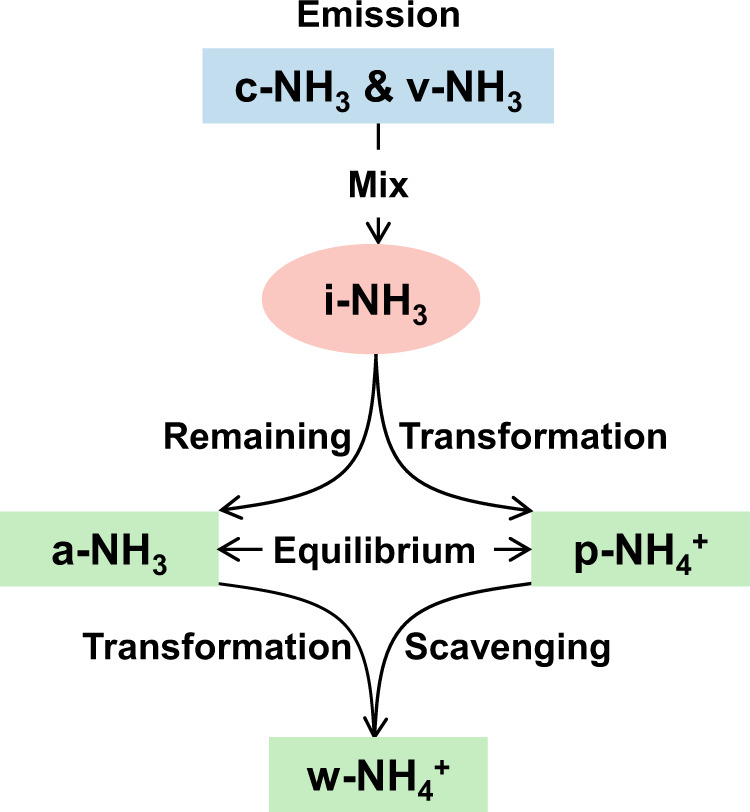
Conceptual framework of atmospheric NH_3_ and NH_4_^+^. It shows the relationships among NH_3_ emissions from combustion-related sources (c-NH_3_) and volatilization sources (v-NH_3_), the initial mixture of c-NH_3_ and v-NH_3_ (i-NH_3_), ambient NH_3_ (a-NH_3_), particulate NH_4_^+^ (p-NH_4_^+^), and precipitation NH_4_^+^ (w-NH_4_^+^). The a-NH_3_ potentially includes the NH_3_ that has not been converted to p-NH_4_^+^ in earlier stages and the fresh NH_3_ emissions in relatively later periods.

**Fig. 2:**
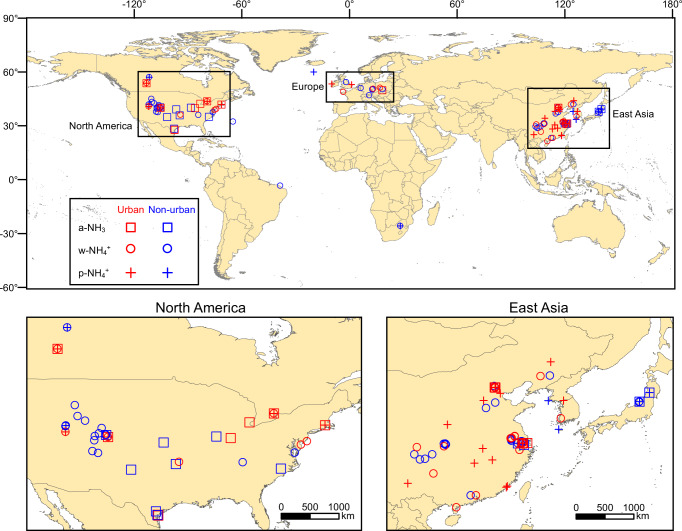
Global and regional maps with the observation sites for *δ*^15^N of ambient NH_3_ (a-NH_3_), particulate NH_4_^+^ (p-NH_4_^+^), and precipitation NH_4_^+^ (w-NH_4_^+^) in East Asia, North America, and Europe. Maps were created by using ArcGIS version 10.5 (Esri Inc., USA). The base map was download from https://hub.arcgis.com/datasets/esri::world-countries-generalized.

**Fig. 3:**
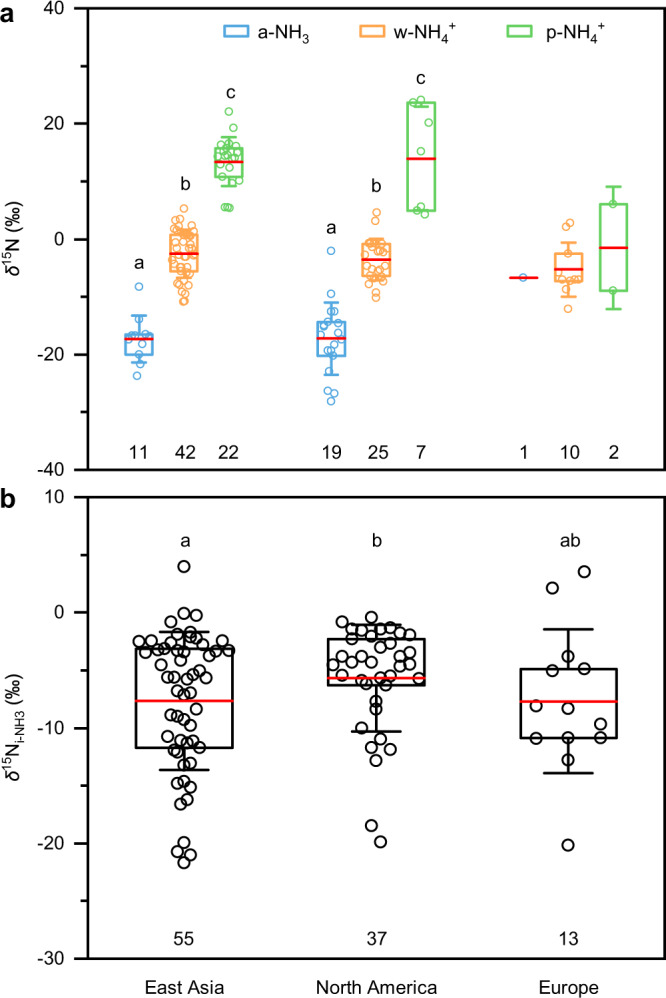
*δ*^15^N of ambient NH_3_ (a-NH_3_), particulate NH_4_^+^ (p-NH_4_^+^), and precipitation NH_4_^+^ (w-NH_4_^+^) (a) and the initial NH_3_ mixture from different sources (*δ*^15^N_i-NH3_) (b) in East Asia, North America, and Europe. Each data point represents the site-based mean *δ*^15^N. Each box encompasses the 25^th^−75^th^ percentiles, whiskers and red lines in boxes are the SD and mean values, respectively. The number below each box is that of observation sites. Note that the site with simultaneous a-NH_3_, w-NH_4_^+^, or p-NH_4_^+^ observation is counted as one site in sub-figure b. In sub-figure a, different letters (**a, b,** and **c**) above the boxes indicate the significant differences (*p* < 0.05) among species in the same region; in sub-figure b, different letters (**a** and **b**) above the boxes indicate the significant differences among the three regions. The *δ*^15^N_a-NH3_ based on the passive samplers have been calibrated by adding 15‰^41^. The *δ*^15^N_i-NH3_ was calculated according to Eqs. (11–13) (detailed in Methods).

**Fig. 4:**
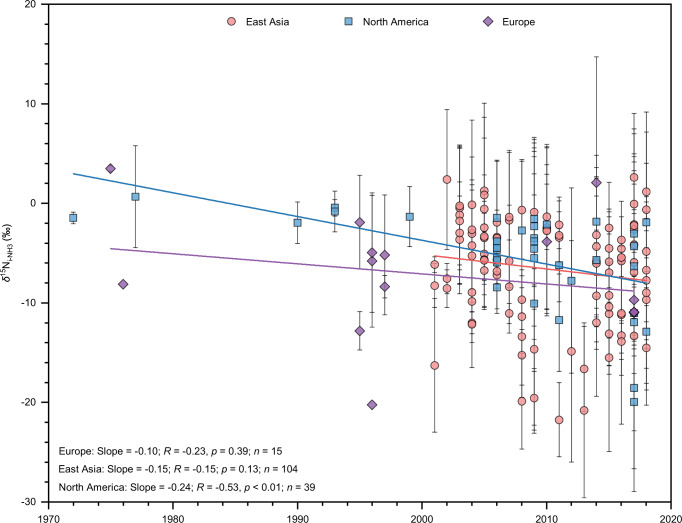
Temporal variations of *δ*^15^N of the initial NH_3_ mixture from different sources (*δ*^15^N_i-NH3_) in East Asia, North America, and Europe. The mean ± SD of replicate measurements at each site in each year is shown. We counted the same site with different years as different observations, given that *δ*^15^N observations at a few sites have been conducted in different sampling years.

**Fig. 5:**
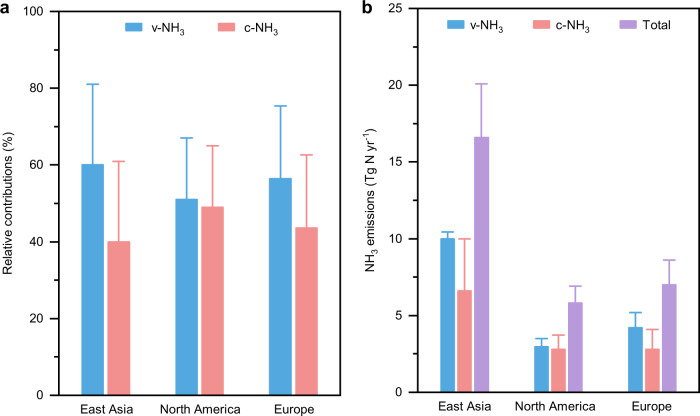
Relative contributions of volatilization NH_3_ (v-NH_3_) and combustion-related NH_3_ (c-NH_3_) sources (a) and their emission amounts (b). The ‘Total’ is the sum of v-NH_3_ and c-NH_3_. Mean±SD is shown.

Here we collated observation data of natural N isotopes (expressed as *δ*^15^N, *δ*^15^N = (^15^N*/*^14^N)_sample_/(^15^N*/*^14^N)_standard_ −1, where atmospheric N_2_ is used as the standard) of primary v-NH_3_ and c-NH_3_ emission sources (Supplementary Fig. 2), NH_3_ gas in the ambient atmosphere (a-NH_3_), NH_4_^+^ in atmospheric particulates (p-NH_4_^+^) and precipitation (w-NH_4_^+^) in East Asia, North America, and Europe (Fig. 1–3; Supplementary Figs. 3 & 4). In combination with the collected data of a-NH_3_ and p-NH_4_^+^ concentrations observed in the above regions, we evaluated *δ*^15^N differences of the initial NH_3_ mixture of v-NH_3_ and c-NH_3_ (i-NH_3_) to a-NH_3_, p-NH_4_^+^, or w-NH_4_^+^, respectively (Supplementary Figs. 5–9; detailed in Methods). Then, based on the source *δ*^15^N, *δ*^15^N difference, *δ*^15^N observation of a-NH_3_, p-NH_4_^+^, and w-NH_4_^+^, and the Stable Isotope Analysis in R model (i.e., the SIAR model; detailed in Methods), we established a set of isotopic methods to calculate relative contributions between v-NH_3_ and c-NH_3_ in above study regions (Fig. 4 & 5; Supplementary Figs. 10–12). Finally, using regional mean fraction values and emission amounts of v-NH_3_, we recalculated the amounts of c-NH_3_ and total NH_3_ emissions in each region (Fig. 5; Supplementary Figs. 13 & 14).

## Results and Discussion

### *δ*^15^N signatures of v-NH_3_ and c-NH_3_

*δ*^15^N for v-NH_3_ (*δ*^15^N_v-NH3_) average −18.9 ± 4.2‰, significantly lower than c-NH_3_ (*δ*^15^N_c-NH3_, averaging 8.0 ± 4.4‰) (Supplementary Fig. 2b, detailed in Methods). The volatilization of NH_3_ includes three main steps, i.e., the equilibrium of NH_4_^+^ ↔ NH_3_, the diffusion of NH_3_ to and away from volatilization sites^37^. The overall isotope effects range from −60‰ to −30‰ (depending on temperature, pH, and cation-exchange capacities of substrates^20,38–40^, causing the low *δ*^15^N_v-NH3_. Differently, *δ*^15^N_c-NH3_ would assemble or be slightly higher than *δ*^15^N of burning materials that are relatively ^15^N-enriched^41^. Distinct *δ*^15^N_v-NH3_ and *δ*^15^N_c-NH3_ provide a unique tool to differentiate the relative contributions between v-NH_3_ and c-NH_3_ in the SIAR isotope mass-balance model.

### *δ*^15^N differences between a-NH_3_, p-NH_4_^+^, or w-NH_4_^+^ and i-NH_3_

The *δ*^15^N of the initial NH_3_ mixture of v-NH_3_ and c-NH_3_ emissions (*δ*^15^N_i-NH3_) integrate their *δ*^15^N signatures and fractional contributions (*F*_v-NH3_ and *F*_c-NH3_, respectively) (Fig. 1, Eq. (1)).

1 $${\delta }^{15}{{{{{{\rm{N}}}}}}}_{{{{{{\rm{i}}}}}}-{{{{{\rm{NH}}}}}}3}={\delta }^{15}{{{{{{\rm{N}}}}}}}_{{{{{{\rm{v}}}}}}-{{{{{\rm{NH}}}}}}3}\times {F}_{{{{{{\rm{v}}}}}}-{{{{{\rm{NH}}}}}}3}+{\delta }^{15}{{{{{{\rm{N}}}}}}}_{{{{{{\rm{c}}}}}}-{{{{{\rm{NH}}}}}}3}\times {F}_{{{{{{\rm{c}}}}}}-{{{{{\rm{NH}}}}}}3}$$

where *F*_v-NH3_ + *F*_c-NH3_ = 1. However, because NH_3_ is very reactive and readily transformed into NH_4_^+^, it is difficult in reality, if not impossible, to directly measure*δ*^15^N_i-NH3_^42,43^.

Practically, site-based *δ*^15^N values of a-NH_3_, p-NH_4_^+^, and w-NH_4_^+^ have been widely measured (Fig. 2). However, their *δ*^15^N, namely *δ*^15^N_a-NH3_, *δ*^15^N_p-NH4+_, and *δ*^15^N_w-NH4+_, cannot be directly used as *δ*^15^N_i-NH3_ to calculate *F*_v-NH3_ and *F*_c-NH3_ (Eq. (1)). First, the i-NH_3_ mixture of v-NH_3_ and c-NH_3_ emissions will be partially converted to p-NH_4_^+^ and w-NH_4_^+^ (Fig. 1). The conversion of NH_3_ to p-NH_4_^+^ has significant isotope effects as a result of either kinetic isotope fractionations (*ε*_k_) during the unidirectional reaction of NH_3_ → p-NH_4_^+^ or equilibrium isotope fractionations (*ε*_eq_) during reversible reactions of NH_3_ ↔ p-NH_4_^+^ (Supplementary Table 3). Accordingly, *δ*^15^N_a-NH3_ and *δ*^15^N_p-NH4+_ differ from *δ*^15^N_i-NH3_ and thus cannot be directly used in Eq. (1)^34,35^. Second, precipitation scavenges both a-NH_3_ and p-NH_4_^+^ via the rainout and washout processes (Fig. 1), but the preferential wet scavenge between a-NH_3_ and p-NH_4_^+^ can potentially cause differences between *δ*^15^N_w-NH4+_ and *δ*^15^N_i-NH3_^44^. Consequently, *δ*^15^N_w-NH4+_ cannot be directly used to calculate *F*_v-NH3_ and *F*_c-NH3_ in Eq. (1) either. According to both simultaneous observations at the same sites (Supplementary Table 4) and non-synchronous observations in the same regions (Fig. 3a), *δ*^15^N in NH_4_^+^ (particularly p-NH_4_^+^) are generally higher than *δ*^15^N_a-NH3_, which is generally negative (Fig. 3a). The main reason is that large equilibrium isotope fractionations occur during the transformation of NH_3_ to NH_4_^+^ (Supplementary Table 3), leading to substantial differences (denoted as ^15^*∆*) of *δ*^15^N_a-NH3_, *δ*^15^N_p-NH4+_, or *δ*^15^N_w-NH4+_ to the corresponding *δ*^15^N_i-NH3_ (^15^*∆*_a-NH3_, ^15^*∆*_p-NH4+_, and ^15^*∆*_w-NH4+_, respectively). In this work, we developed a new set of methods to constrain ^15^*∆*_a-NH3_, ^15^*∆*_p-NH4+_, and ^15^*∆*_w-NH4+_, and then reconstruct *δ*^15^N_i-NH3_ (detailed in Methods).

Generally, based on simultaneous observation data of seasonal mean *C*_a-NH3_, *C*_p-NH4+_, *δ*^15^N_a-NH3_, *δ*^15^N_p-NH4+_, and *δ*^15^N_w-NH4+_ at six sites (Supplementary Table 5), we estimated corresponding *δ*^15^N_i-NH3_, ^15^*∆*_a-NH3_, ^15^*∆*_p-NH4+_, and ^15^*∆*_w-NH4+_ (Eqs. (7–10); detailed in Methods). Then, we established the relationships between ^15^*∆*_a-NH3_, ^15^*∆*_p-NH4+_, or ^15^*∆*_w-NH4+_ and atmospheric NH_3_ conversion ratios (expressed as *f*_p-NH4+_, i.e., *C*_p-NH4+_/(*C*_a-NH3_ + *C*_p-NH4+_)) (Supplementary Fig. 5). These relationships show that ^15^*∆*_a-NH3_, ^15^*∆*_p-NH4+_, and ^15^*∆*_w-NH4+_ decrease with an increase of *f*_p-NH4+_ (Supplementary Fig. 5). ^15^*∆*_a-NH3_ and ^15^*∆*_p-NH4+_ vary linearly with the reaction degree of an open system (i.e., *f*_p-NH4+_) (Supplementary Fig. 5a) consistent with the prediction of isotopic theory^45^. The ^15^*∆*_w-NH4+_ includes ^15^*∆*_a-NH3_ and ^15^*∆*_p-NH4+_ because precipitation scavenges a-NH_3_ and p-NH_4_^+^ via the rainout and washout processes (Supplementary Fig. 5b).

Meanwhile, we examined the impact of historical NO_x_ and sulfur dioxide (SO_2_) reductions on mean annual *f*_p-NH4+_ (Supplementary Fig. 6a–f). The *f*_p-NH4+_ generally decreased from 1990 to 2017 in Europe and 2004 to 2018 in North America, but did not vary clearly from 2000 to 2018 in East Asia (Supplementary Fig. 7). Then, based on the relationships between ^15^*∆*_a-NH3_, ^15^*∆*_p-NH4+_, or ^15^*∆*_w-NH4+_ and *f*_p-NH4+_ (Supplementary Fig. 5) and mean annual *f*_p-NH4+_ values (Supplementary Fig. 7), we calculated mean annual ^15^*∆*_a-NH3_, ^15^*∆*_p-NH4+_, and ^15^*∆*_w-NH4+_ in each region (Supplementary Fig. 8), which were further used to calculate the corresponding *δ*^15^N_i-NH3_ (Fig. 3b) of site-based *δ*^15^N_a-NH3_, *δ*^15^N_p-NH4+_, or *δ*^15^N_w-NH4+_ (Fig. 3a) (detailed in Methods).

### Spatial and temporal patterns of *δ*^15^N_i-NH3_ variations

Spatially, *δ*^15^N_i-NH3_ is higher in North America (−5.7 ± 4.6‰) than in Europe (−7.7 ± 6.3‰) and East Asia (−8.0 ± 6.0‰) (Fig. 3b). Because the *δ*^15^N_c-NH3_ is distinctly higher than the *δ*^15^N_v-NH3_ (Supplementary Fig. 2), the emission strength of c-NH_3_ relative to v-NH_3_ in North America is higher than that in East Asia and Europe. On the one hand, the emission inventories also show that the proportion of v-NH_3_ in North America is lower than that in East Asia and Europe (Supplementary Fig. 1b). On the other hand, because energy consumption is the primary source of c-NH_3_ and fertilizer consumption and animal manure are the source of v-NH_3_ (Supplementary Fig. 15a, b), the energy consumption ratio to fertilizer consumption and animal manure is higher in North America than in East Asia and Europe (Supplementary Fig. 15c).

*δ*^15^N_i-NH3_ decreased significantly between 1972 and 2018 in North America (*p* < 0.01) and decreased slightly from 2001 to 2018 in East Asia (*p* = 0.13) and 1974–2017 in Europe (*p* = 0.39) (Fig. 4). These results suggest that the emission strength of v-NH_3_ relative to c-NH_3_ generally increased during the past decades in our three study regions, especially in North America. This finding coincided with increasing fertilizer consumption and animal manure in East Asia and North America over the past decades (Supplementary Fig. 16).

### Relative contributions and amounts of v-NH_3_ and c-NH_3_ emissions

We considered ^15^*∆*_a-NH3_, ^15^*∆*_p-NH4+_, and ^15^*∆*_w-NH4+_ (detailed in Methods) in Eq. (1) to establish a set of new isotope mass-balance equations to calculate *F*_v-NH3_ and *F*_c-NH3_ by using *δ*^15^N_a-NH3_, *δ*^15^N_p-NH4+_, or *δ*^15^N_w-NH4+_ in each region (Eqs. (2–4), respectively).

2 $${\delta }^{15}{{{{{{\rm{N}}}}}}}_{{{{{{\rm{a}}}}}}-{{{{{\rm{NH}}}}}}3}={\delta }^{15}{{{{{{\rm{N}}}}}}}_{{{{{{\rm{v}}}}}}-{{{{{\rm{NH}}}}}}3}\times {F}_{{{{{{\rm{v}}}}}}-{{{{{\rm{NH}}}}}}3}+{\delta }^{15}{{{{{{\rm{N}}}}}}}_{{{{{{\rm{c}}}}}}-{{{{{\rm{NH}}}}}}3}\times {F}_{{{{{{\rm{c}}}}}}-{{{{{\rm{NH}}}}}}3}+{}^{15}\varDelta _{{{{{{\rm{a}}}}}}-{{{{{\rm{NH}}}}}}3}$$

3 $${\delta }^{15}{{{{{{\rm{N}}}}}}}_{{{{{{\rm{p}}}}}}-{{{{{\rm{NH4}}}}}}+}={\delta }^{15}{{{{{{\rm{N}}}}}}}_{{{{{{\rm{v}}}}}}-{{{{{\rm{NH}}}}}}3}\times {F}_{{{{{{\rm{v}}}}}}-{{{{{\rm{NH}}}}}}3}+{\delta }^{15}{{{{{{\rm{N}}}}}}}_{{{{{{\rm{c}}}}}}-{{{{{\rm{NH}}}}}}3}\times {F}_{{{{{{\rm{c}}}}}}-{{{{{\rm{NH}}}}}}3}+{}^{15}\varDelta _{{{{{{\rm{p}}}}}}-{{{{{\rm{NH}}}}}}4+}$$

4 $${\delta }^{15}{{{{{{\rm{N}}}}}}}_{{{{{{\rm{w}}}}}}-{{{{{\rm{NH4}}}}}}+}={\delta }^{15}{{{{{{\rm{N}}}}}}}_{{{{{{\rm{v}}}}}}-{{{{{\rm{NH}}}}}}3}\times {F}_{{{{{{\rm{v}}}}}}-{{{{{\rm{NH}}}}}}3}+{\delta }^{15}{{{{{{\rm{N}}}}}}}_{{{{{{\rm{c}}}}}}-{{{{{\rm{NH}}}}}}3}\times {F}_{{{{{{\rm{c}}}}}}-{{{{{\rm{NH}}}}}}3}+{}^{15}\varDelta _{{{{{{\rm{w}}}}}}-{{{{{\rm{NH}}}}}}4+}$$

*F*_v-NH3_ and *F*_c-NH3_ values were calculated by the SIAR model (detailed in Methods).

*F*_c-NH3_ averages 40 ± 21% in East Asia, 49 ± 16% in North America, and 44 ± 19% in Europe (Fig. 5a), which confirms higher emission strength of c-NH_3_ relative to v-NH_3_ in North America than in East Asia and Europe. These estimations based on isotope methods are generally higher than the fractions of c-NH_3_ emissions in corresponding regions (5–10%; Supplementary Fig. 1b) or the globe based on emission inventories (30%^10,23,31^). One possible explanation is that the study regions are hotspots of globally high c-NH_3_ emissions^24^. Supportively, the total consumption of fossil fuels in these regions accounts for more than 60% of the global energy consumption (Supplementary Fig. 15a), though their areas account for only 24% of the worldwide land area^46^. Further, the spatial pattern of *F*_c-NH3_ (North America > Europe > East Asia; Fig. 5a) assembles that of the energy consumption ratio to fertilizer consumption and animal manure (Supplementary Fig. 15c). Based on *F*_v-NH3_ in our study and explicit amounts of v-NH_3_ (*A*_v-NH3_) in emission inventories (Fig. 5), we further estimated the amounts of c-NH_3_ (*A*_c-NH3_) and total NH_3_ emissions (detailed in Methods), which average 6.6 ± 3.4 Tg N yr^−1^ and 16.6 ± 3.5 Tg N yr^−1^ in East Asia, 2.8 ± 0.9 Tg N yr^−1^ and 5.8 ± 1.1 Tg N yr^−1^ in North America, and 2.8 ± 1.3 Tg N yr^−1^ and 7.0 ± 1.6 Tg N yr^−1^ in Europe, respectively (Fig. 5b). The highest energy consumption in East Asia supports its highest *A*_c-NH3_ among the three study regions (Fig. 5b & Supplementary Fig. 15a).

Our new estimates of total NH_3_ emission in Europe are very close to its total NH_x_ deposition (Supplementary Fig. 13). In China and the United States, the NH_x_ deposition fluxes could be explained by our updated total NH_3_ emissions (Supplementary Fig. 13). Lower deposition than the emission was observed in China and the United States because part of the NH_3_ emissions was diffused or deposited out of these polluted areas^24,47^. Before this work, total NH_3_ emissions based on statistical inventories in China, the United States, and Europe were all distinctly lower than the corresponding NH_x_ deposition (Supplementary Fig. 13). Accordingly, our results provided new estimates on c-NH_3_ and total NH_3_ emissions. However, because the v-NH_3_ underestimation may still exist^48^, the contribution of the c-NH_3_ underestimation to the underestimation of total NH_3_ emissions and the mismatches between regional NH_3_ emissions and NH_x_ deposition (Supplementary Fig. 13) remains uncertain.

Temporally, *F*_c-NH3_ has decreased significantly (*p* < 0.01) over the past decades in North America (Supplementary Fig. 12a), leading to increasing ratios of *F*_v-NH3_ to *F*_c-NH3_ (*p* < 0.01) and also *A*_v-NH3_ to *A*_c-NH3_ (*p* < 0.05) generally from lower than 1.0 to higher than 1.0 (Supplementary Figs. 12b & 14b). *F*_c-NH3_ in East Asia and Europe has decreased slightly (*p* = 0.12 and *p* = 0.26, respectively) (Supplementary Fig. 12a), leading to slightly increasing ratios of *F*_v-NH3_ to *F*_c-NH3_ (*p* = 0.12 and *p* = 0.51, respectively) and also *A*_v-NH3_ to *A*_c-NH3_ over the past decades (*p* = 0.25 and *p* = 0.61, respectively) (Supplementary Figs. 12b & 14b). In East Asia and North America, the temporally increasing v-NH_3_ emissions relative to c-NH_3_ are supported by the increasing fertilizer consumption and animal manure production (Supplementary Fig. 16). Emission inventories also showed the rapid increase of the relative contribution of v-NH_3_ in North America (Supplementary Fig. 1b). These temporal variations in North America revealed that the more dominant NH_3_ emission has shifted from c-NH_3_ to v-NH_3_ sources over the past decades. Rapidly increasing v-NH_3_ emissions may be one of the reasons for the shift from nitrate-dominated to NH_4_^+^-dominated N deposition in the United States^7^.

In this study, the non-urban *F*_c-NH3_ does not change with the corresponding distance between the sampling site and the nearest urban area (Supplementary Fig. 11). Meanwhile, there are no significant differences in *F*_c-NH3_ between urban (73 sites) and non-urban (65 sites) or between agricultural (32 sites) and non-agricultural sites (33 sites) (Supplementary Fig. 11). Similar numbers of replicate sites between the above surface environments reduce the risks of over- or under-estimating c-NH_3_ or v-NH_3_ contributions. Accordingly, the *F*_c-NH3_ at urban or non-urban sites is not substantially influenced by local NH_3_ emissions but reflects the source diversity and transporting/mixing complexity of regional NH_3_ emissions. There has been much evidence from ground monitoring, satellite observations, and emission inventories to show the co-occurrence of c-NH_3_ and v-NH_3_ emissions and extensive NH_3_ or NH_4_^+^ transportation and mixing among landscapes. Firstly, v-NH_3_ sources (mainly from solid wastes and sewages) have comparable emission strengths with c-NH_3_ sources (mainly from fossil fuel combustion) in urban areas. Human excreta contributed 11.4% to the total NH_3_ emissions in Shanghai urban of eastern China^49^. The v-NH_3_ from urban green space contributed up to 60% to ambient NH_3_ in Qingdao in northern China^50^. The urban NH_3_ concentrations influenced by the v-NH_3_ from urban waste containers, sewage systems, humans, and open markets were 2.5 times higher than that of traffic-influenced urban areas in Spain^51^. Ambient NH_3_ concentrations in the Beijing urban peaked when fertilizer was intensively applied on the North China Plain^52^. Secondly, the c-NH_3_ emission (mainly wildfire, fossil fuel and crop residue combustion) is undoubtedly as significant as the v-NH_3_ (mainly fertilizer application and live stocks) in non-urban areas. For example, the c-NH_3_ from biomass burning control seasonal variations of surface NH_3_ concentrations in major disturbed regions of the Northern Hemisphere, which is even stronger in the Southern Hemisphere with frequent wildfires (Supplementary Table 2). Based on a data synthesis (Supplementary Table 2), ambient NH_3_ concentrations during wildfire smoke-impacted periods could be enhanced by a factor of 2–20 compared to periods with no wildfires. Besides, our results, as well as previous studies, show that v-NH_3_ contributed 58% (31–87%) for sites in Beijing urban^34,35,53^, while the c-NH_3_ accounted for 33% (25–69%) in the total NH_4_^+^ deposition of the cropland 370 km far from the Beijing urban^11^. Collectively, site-based δ^15^N_a-NH3_ and δ^15^N_NH4+_ represent mixing signatures and can be feasibly used to evaluate regional c-NH_3_ and v-NH_3_ emissions.

### Implications and uncertainties

This study demonstrates that c-NH_3_ emissions have been considerably underestimated in regions with significant anthropogenic sources. In the process, the new estimates of c-NH_3_ emissions have brought measured NH_x_ deposition data into closer agreement with emissions. Our conclusion about the underestimation of c-NH_3_ emission has important implications for control measures since the current efforts have been focused almost exclusively on agricultural sources. Although the marginal abatement cost of NH_3_ emissions is only 10% of the global NO_x_ emission, with 162 billion US dollars net benefit, the reduction can effectively improve air pollution and its negative impact^54,55^. For example, measures to reduce c-NH_3_ emissions should be considered in the methods for mitigating v-NH_3_ emissions by improved farm management practices with N use reductions, deep machine placement of fertilizer, enhanced-efficiency fertilizer use, and enhanced manure management in the agriculture sector^56,57^. The revelation of high c-NH_3_ emissions also implies that the potential, costs, and impacts of NH_3_ emissions reduction need to be re-assessed. Steps to reduce c-NH_3_ emissions may alleviate the pressure of reducing agricultural NH_3_ emissions and achieve ‘win-win’ outcomes for agricultural production and food supply, human and environmental health^2,56^.

The uncertainty of this study lies in the technical difficulty in measuring *δ*^15^N for all NH_3_ emission sources in each region. It is even more challenging to conduct simultaneous observations on *C*_a-NH3_, *C*_p-NH4+_, *δ*^15^N_a-NH3_, *δ*^15^N_p-NH4+_, and *δ*^15^N_w-NH4+_ among landscapes. As a result, a limited number of data is available in terms of observation sites and their spatial distribution in the three regions, and the corresponding data comparison among the three regions is preliminary in the current stage. Moreover, *δ*^15^N observations focus largely on the middle- and low-latitudes and low altitudes of the Northern Hemisphere but rare on the high mountains and high latitudes of the Northern Hemisphere and the Southern Hemisphere. More observations on the concentration and *δ*^15^N parameters of NH_3_ and NH_4_^+^ in both source emissions and deposition are necessary to improve the isotope source apportionment, especially in remote areas. Finally, despite the updated regional c-NH_3_ and total NH_3_ emissions, future in-depth cooperation among different observational and modeling methods should be encouraged.

## Methods

### *δ*^15^N of major NH_3_ emission sources

We collected the *δ*^15^N data of major NH_3_ emission sources (Supplementary Fig. 2a) from 17 relevant publications (by December 2020). For c-NH_3_, the proportional contributions of global NH_3_ emissions from vehicle exhausts (ve-NH_3_), coal combustion (cc-NH_3_), and biomass burning (bb-NH_3_) (1.3, 6.3, and 8.2 Tg N yr^−1^, respectively^10,23,31^) in total c-NH_3_ emissions (15.9 Tg N yr^−1^) are 8%, 40%, and 52%, respectively (Eq. (5)). Accordingly, we calculated the *δ*^15^N_c-NH3_ by a mass-balance method (Eq. (5)).

5 $${\delta }^{15}{{{{{{\rm{N}}}}}}}_{{{{{{\rm{c}}}}}}-{{{{{\rm{NH}}}}}}3}={\delta }^{15}{{{{{{\rm{N}}}}}}}_{{{{{{\rm{ve}}}}}}-{{{{{\rm{NH}}}}}}3}\times 8 \%+{\delta }^{15}{{{{{{\rm{N}}}}}}}_{{{{{{\rm{cc}}}}}}-{{{{{\rm{NH}}}}}}3}\times 40\%+{\delta }^{15}{{{{{{\rm{N}}}}}}}_{{{{{{\rm{bb}}}}}}-{{{{{\rm{NH}}}}}}3}\times 52\%$$

For v-NH_3_, global NH_3_ emissions from fertilizer application (fa-NH_3_) and waste materials (wm-NH_3_) account for 56% and 44% of the total v-NH_3_ emission, respectively^10^. Similarly, we calculated the *δ*^15^N_v-NH3_ by Eq. (6).

6 $${\delta }^{15}{{{{{{\rm{N}}}}}}}_{{{{{{\rm{v}}}}}}-{{{{{\rm{NH}}}}}}3}={\delta }^{15}{{{{{{\rm{N}}}}}}}_{{{{{{\rm{fa}}}}}}-{{{{{\rm{NH}}}}}}3}\times 56\%+{\delta }^{15}{{{{{{\rm{N}}}}}}}_{{{{{{\rm{wm}}}}}}-{{{{{\rm{NH}}}}}}3}\times 44\%$$

The standard deviation (SD) of *δ*^15^N_c-NH3_ and *δ*^15^N_v-NH3_ is propagated errors estimated using the Monte Carlo method (MCM). Briefly, we ran 10000 trials for the MCM in the software of Microsoft Excel-Add-In and calibrated the SD to match the corresponding true values.

### Atmospheric *δ*^15^N_a-NH3_, *δ*^15^N_p-NH4+_, and *δ*^15^N_w-NH4+_ observations

Keywords used for the search are ‘nitrogen isotope’, ‘ammonia/NH_3_’, ‘ammonium/NH_4_^+^’, ‘rainfall’, ‘rain’, ‘rain water’, ‘precipitation’, ‘aerosol’, and ‘particulate’. The databases include the Web of Science (http://isiknowledge.com), Google Scholar (http://scholar.google.com.hk), and Baidu Scholar (http://xueshu.baidu.com). By December 2020, there are 18 publications on *δ*^15^N_a-NH3_ (listed in Supplementary Text 1), 43 publications on *δ*^15^N_w-NH4+_ (listed in Supplementary Text 2), and 28 publications on *δ*^15^N_p-NH4+_ (listed in Supplementary Text 3). Data in the figures of these publications were extracted using the software of Web Plot Digitizer (Version 4.2, San Francisco, California, USA).

Spatial distributions of the sites with *δ*^15^N_a-NH3_, *δ*^15^N_p-NH4+_, and *δ*^15^N_w-NH4+_ observations are shown in Fig. 2. When counting the same site with observations in different years as one site only, there are 387 measurements of *δ*^15^N_a-NH3_ at 32 sites (including 12 sites in East Asia, 19 sites in North America, and one site in Europe), 857 measurements of *δ*^15^N_p-NH4+_ at 33 sites (including 22 sites in East Asia, seven sites in North America, two sites in Europe, one site in Africa, and one site in Atlantic), and 1540 measurements of *δ*^15^N_w-NH4+_ at 80 sites (including 42 sites in East Asia, 25 sites in North America, ten sites in Europe, one site in South America, one site in Africa, and one site in Atlantic) (Fig. 2 & Supplementary Fig. 3). The surface land types of observation sites were identified according to descriptions in original publications. There are 73 urban sites (mainly constructed lands) and 65 non-urban sites (mainly including 32 agricultural sites and 33 non-agricultural sites) with *δ*^15^N_a-NH3_, *δ*^15^N_p-NH4+_, or *δ*^15^N_w-NH4+_ observations in the study areas of East Asia, North America, and Europe (Supplementary Fig. 4a, b).

We only analyzed the data in the major areas of East Asia during 2001–2018, North America during 1972–2018, and Europe during 1974–2017 due to the sparsity of available data out of these areas (Fig. 2 & 4). It should be noted that analytical methods of ^15^N_a-NH3_, *δ*^15^N_p-NH4+_, and *δ*^15^N_w-NH4+_ differ among studies, including converting to 1) N_2_ as an end product using the Elemental Analyzer combustion method or 2) N_2_O as an end product using the bromate oxidation and azide or hydroxylamine reduction, or using the persulfate oxidation and denitrifier method^58^, but such difference would not change the spatiotemporal patterns of *δ*^15^N_i-NH3_ (Fig. 3 & 4) because the analytical accuracy is generally better than ±0.7‰. In addition, 9%, 16%, and 75% of the *δ*^15^N observations were conducted across cooler seasons, warmer seasons, and the whole year, respectively. The seasonal differences in NH_3_ emissions would not substantially influence the spatiotemporal patterns of *δ*^15^N_i-NH3_ (Fig. 3 & 4). Moreover, all observation sites in this study are more than 1 km away from obvious local emission sources, excluding the influence of a single source.

### Atmospheric *C*_a-NH3_ and *C*_p-NH4+_ observations

‘Atmospheric ammonia/NH_3_’, ‘particulate ammonium/NH_4_^+^’, and ‘aerosol ammonium/NH_4_^+^’ were used as keywords to search publications for the concentration of a-NH_3_ and p-NH_4_^+^ (*C*_a-NH3_ and *C*_p-NH4+_, respectively) in the same databases as described above. There are 107 publications published by July 2021 (listed in Supplementary Text 4) with simultaneous observations on *C*_a-NH3_ and *C*_p-NH4+_. To describe temporal variations of *C*_p-NH4+_/(*C*_a-NH3_ + *C*_p-NH4+_) values (i.e., *f*_p-NH4+_ values) in each region (Supplementary Fig. 7), we counted the same site with different years as different observations because few sites observed *C*_a-NH3_ and *C*_p-NH4+_ for many years. We only used data with an observation period exceeding six months to improve the estimation of annual *f*_p-NH4+_ values. According to this criterion, there are 262 sites in East Asia during 1993–2018, 459 sites in North America during 1986–2018, and 1018 sites in Europe during 1981–2017 (Supplementary Fig. 7).

### Differences of *δ*^15^N_a-NH3_, *δ*^15^N_p-NH4+_, or *δ*^15^N_w-NH4+_ from i-NH_3_

Based on simultaneous observations of seasonal *C*_a-NH3_, *C*_p-NH4+_, *δ*^15^N_a-NH3_, *δ*^15^N_p-NH4+_, and *δ*^15^N_w-NH4+_ values at the same sites (Supplementary Table 5), we calculated the *δ*^15^N_i-NH3_ by the following isotope mass-balance equation (Eq. (7)).

7 $${\delta }^{15}{{{{{{\rm{N}}}}}}}_{{{{{{\rm{i}}}}}}-{{{{{\rm{NH}}}}}}3}={\delta }^{15}{{{{{{\rm{N}}}}}}}_{{{{{{\rm{a}}}}}}-{{{{{\rm{NH}}}}}}3}\times {f}_{{{{{{\rm{a}}}}}}-{{{{{\rm{NH}}}}}}3}+{\delta }^{15}{{{{{{\rm{N}}}}}}}_{{{{{{\rm{p}}}}}}-{{{{{\rm{NH}}}}}}4+}\times {f}_{{{{{{\rm{p}}}}}}-{{{{{\rm{NH}}}}}}4+}$$

where *f*_a-NH3_ = *C*_a-NH3_ / (*C*_a-NH3_ + *C*_p-NH4+_) and *f*_p-NH4+_ = *C*_p-NH4+_ / (*C*_a-NH3_ + *C*_p-NH4+_).

Then, we calculated the differences between *δ*^15^N_i-NH3_ and *δ*^15^N_a-NH3_ (^15^*∆*_a-NH3_, Eq. (8)), between *δ*^15^N_i-NH3_ and *δ*^15^N_p-NH4+_ (^15^*∆*_p-NH4+_, Eq. (9)), between *δ*^15^N_i-NH3_ and *δ*^15^N_w-NH4+_ (^15^*∆*_w-NH4+_, Eq. (10)) for the same sites with simultaneous observations of seasonal *C*_a-NH3_, *C*_p-NH4+_, *δ*^15^N_a-NH3_, *δ*^15^N_p-NH4+_, and *δ*^15^N_w-NH4+_ values (Supplementary Table 5).

8 $${}^{15}\varDelta _{{{{{{\rm{a}}}}}}-{{{{{\rm{NH}}}}}}3}={\delta }^{15}{{{{{{\rm{N}}}}}}}_{{{{{{\rm{a}}}}}}-{{{{{\rm{NH}}}}}}3}-{\delta }^{15}{{{{{{\rm{N}}}}}}}_{{{{{{\rm{i}}}}}}-{{{{{\rm{NH}}}}}}3}$$

9 $${}^{15}\varDelta _{{{{{{\rm{p}}}}}}-{{{{{\rm{NH}}}}}}4+}={\delta }^{15}{{{{{{\rm{N}}}}}}}_{{{{{{\rm{p}}}}}}-{{{{{\rm{NH}}}}}}4+}-{\delta }^{15}{{{{{{\rm{N}}}}}}}_{{{{{{\rm{i}}}}}}-{{{{{\rm{NH}}}}}}3}$$

10 $${}^{15}\varDelta _{{{{{{\rm{w}}}}}}-{{{{{\rm{NH}}}}}}4+}={\delta }^{15}{{{{{{\rm{N}}}}}}}_{{{{{{\rm{w}}}}}}-{{{{{\rm{NH}}}}}}4+}-{\delta }^{15}{{{{{{\rm{N}}}}}}}_{{{{{{\rm{i}}}}}}-{{{{{\rm{NH}}}}}}3}$$

Based on the relationships in Supplementary Fig. 5 and mean annual *f*_p-NH4+_ values in Supplementary Fig. 7, we calculated the mean annual ^15^*∆*_a-NH3_, ^15^*∆*_p-NH4+_, and ^15^*∆*_w-NH4+_ in each region (Supplementary Fig. 8). Because no clear trends were observed in *f*_p-NH4+_ between 2001–2018 in East Asia and before implementing emission reduction measures in Europe (i.e., 1971–1989 in this study) and in North America (i.e., 1971–2004 in this study)^59–61^ (Supplementary Fig. 7), we calculated isotope effect values by using the mean *f*_p-NH4+_ during above-mentioned years in the corresponding region (Supplementary Fig. 8). In other words, the same values are assumed for ^15^*∆*_a-NH3_, ^15^*∆*_p-NH4+_, or ^15^*∆*_w-NH4+_ during these years in the region (Supplementary Fig. 8).

To examine the applicability of our method for estimating and calibrating ^15^*∆* values, we used the mean annual ^15^*∆* values and simultaneous *δ*^15^N_a-NH3_ and *δ*^15^N_p-NH4+_ observations, *δ*^15^N_a-NH3_ and *δ*^15^N_w-NH4+_ observations, or *δ*^15^N_p-NH4+_ and *δ*^15^N_w-NH4+_ observations at the same sites (Supplementary Table 4) to calculate their corresponding *δ*^15^N_i-NH3_ (denoted as *δ*^15^N_i-NH3(a-NH3)_, *δ*^15^N_i-NH3(p-NH4+)_, or *δ*^15^N_i-NH3(w-NH4+)_; Eq. (11 − 13), respectively).

11 $${\delta }^{15}{{{{{{\rm{N}}}}}}}_{{{{{{\rm{i}}}}}}-{{{{{\rm{NH}}}}}}3({{{{{\rm{a}}}}}}-{{{{{\rm{NH}}}}}}3)}={\delta }^{15}{{{{{{\rm{N}}}}}}}_{{{{{{\rm{a}}}}}}-{{{{{\rm{NH}}}}}}3}-{}^{15}\varDelta _{{{{{{\rm{a}}}}}}-{{{{{\rm{NH}}}}}}3}$$

12 $${\delta }^{15}{{{{{{\rm{N}}}}}}}_{{{{{{\rm{i}}}}}}-{{{{{\rm{NH}}}}}}3({{{{{\rm{p}}}}}}-{{{{{\rm{NH}}}}}}4+)}={\delta }^{15}{{{{{{\rm{N}}}}}}}_{{{{{{\rm{p}}}}}}-{{{{{\rm{NH}}}}}}4+}-{}^{15}\varDelta _{{{{{{\rm{p}}}}}}-{{{{{\rm{NH}}}}}}4+}$$

13 $${\delta }^{15}{{{{{{\rm{N}}}}}}}_{{{{{{\rm{i}}}}}}-{{{{{\rm{NH}}}}}}3({{{{{\rm{w}}}}}}-{{{{{\rm{NH}}}}}}4+)}={\delta }^{15}{{{{{{\rm{N}}}}}}}_{{{{{{\rm{w}}}}}}-{{{{{\rm{NH}}}}}}4+}-{}^{15}\varDelta _{{{{{{\rm{w}}}}}}-{{{{{\rm{NH}}}}}}4+}$$

We found that differences between *δ*^15^N_i-NH3(a-NH3)_ and *δ*^15^N_i-NH3(p-NH4+)_, *δ*^15^N_i-NH3(a-NH3)_ and *δ*^15^N_i-NH3(w-NH4+)_, *δ*^15^N_i-NH3(p-NH4+)_ and *δ*^15^N_i-NH3(w-NH4+)_ are negligible, averaging 0.4 ± 2.3‰, −0.2 ± 1.3‰, and −0.4 ± 2.5‰, respectively (Supplementary Fig. 9). This demonstrates that the mean annual ^15^*∆*_a-NH3_, ^15^*∆*_p-NH4+_, and ^15^*∆*_w-NH4+_ values (Supplementary Fig. 8) estimated by the mean annual *f*_p-NH4+_ values (Supplementary Fig. 7) can be used to calculate corresponding *δ*^15^N_i-NH3_ (Fig. 3b) of site-based *δ*^15^N_a-NH3_, *δ*^15^N_p-NH4+_, or *δ*^15^N_w-NH4+_ (Fig. 3a) and to estimate the source contributions using the SIAR model.

### Relative contributions and amounts of v-NH_3_ and c-NH_3_ emissions

Site-based *F*_v-NH3_ and *F*_c-NH3_ values of each region (Eqs. (2−4)) were calculated using the SIAR model^62^. Because each of our calculations has only two end members, the SIAR model can determine the *F*_v-NH3_ and *F*_c-NH3_ values. Moreover, this model allows us to incorporate isotope effects (by inputting mean annual ^15^*∆*_a-NH3_, ^15^*∆*_p-NH4+_, and ^15^*∆*_w-NH4+_ in our calculations; Supplementary Fig. 8), the variabilities in *δ*^15^N of both sources (by inputting mean ± SD of *δ*^15^N_v-NH3_ and *δ*^15^N_c-NH3_; Supplementary Fig. 2b) and the mixture (by inputting all replicate measurements of *δ*^15^N_a-NH3_, *δ*^15^N_p-NH4+_, or *δ*^15^N_w-NH4+_ at each site) into the source contributions. In each run, the percentage data (*n* = 10000) output from the SIAR model was used to calculate the mean ± SD values of corresponding *F*_v-NH3_ and *F*_c-NH3_ at each site, then site-based mean ± SD values of *F*_v-NH3_ and *F*_c-NH3_ (Supplementary Figs. 10 & 11) were used to calculate the mean ± SD values of each region (Fig. 5a). The mean annual *F*_v-NH3_ and *F*_c-NH3_ were calculated by inputting all replicate measurements of *δ*^15^N_a-NH3_, *δ*^15^N_p-NH4+_, or *δ*^15^N_w-NH4+_ in each year at each site (Fig. 4; Supplementary Fig. 12).

Based on the amount of the v-NH_3_ emission (*A*_v-NH3_), we calculated corresponding amounts of total NH_3_ emissions (*A*_total_, Eq. (14)) and the c-NH_3_ emission (*A*_c-NH3_, Eq. (15)).

14 $${A}_{{{{{{\rm{total}}}}}}}={A}_{{{{{{\rm{v}}}}}}-{{{{{\rm{NH}}}}}}3}/{F}_{{{{{{\rm{v}}}}}}-{{{{{\rm{NH}}}}}}3}$$

15 $${A}_{{{{{{\rm{c}}}}}}-{{{{{\rm{NH}}}}}}3}={A}_{{{{{{\rm{total}}}}}}}-{A}_{{{{{{\rm{v}}}}}}-{{{{{\rm{NH}}}}}}3}$$

Regional mean ± SD of *A*_v-NH3_ and *F*_v-NH3_ were used to calculate regional mean ± SD of *A*_total_ and *A*_c-NH3_ (Fig. 5). The annual *A*_c-NH3_ (Supplementary Fig. 14a) was calculated using the mean *A*_v-NH3_ (Supplementary Fig. 1a) and mean ± SD of site-based *F*_v-NH3_ values in each year (Supplementary Fig. 12). The SDs of *A*_total_ and *A*_c-NH3_ values were propagated errors estimated by using the same MCM described above.

### Statistical analyses

The SPSS 18.0 software package (SPSS Science, Chicago, USA) and Origin 2016 statistical package (OriginLab Corporation, USA) for Windows were used for data analyses in this study. The Tukey honest significant difference (Tukey HSD) and the least significant difference (LSD) tests of the one-way analysis of variance (ANOVA) were used to identify significant differences in *δ*^15^N among a-NH_3_, w-NH_4_^+^, and p-NH_4_^+^ (Fig. 3a), East Asia, North America, and Europe (Fig. 3b), major sources (Supplementary Fig. 2), and urban and non-urban sites or agricultural and non-agricultural sites (Supplementary Fig. 4), in *F*_c-NH3_ values among East Asia, North America, and Europe (Supplementary Fig. 10), and urban and non-urban sites or agricultural and non-agricultural sites (Supplementary Fig. 11a). Linear regressions were used to examine correlations between sampling years and *δ*^15^N_i-NH3_ (Fig. 4), ^15^*∆*_a-NH3_, ^15^*∆*_p-NH4+_, or ^15^*∆*_w-NH4+_ and *f*_p-NH4+_ values (Supplementary Fig. 5), *f*_p-NH4+_ values and NO_x_ and SO_2_ emissions (Supplementary Fig. 6), *F*_c-NH3_ values at non-urban sites with the corresponding distances from the edge of the nearest urban area (Supplementary Fig. 11b), sampling years and *F*_c-NH3_ or *F*_v-NH3_*/F*_c-NH3_ ratio (Supplementary Fig. 12), and sampling years and *A*_c-NH3_ or *A*_v-NH3_*/A*_c-NH3_ ratio (Supplementary Fig. 14). Statistically significant differences were set at *p* < 0.05 or as otherwise stated. The maps with the observation sites for δ^15^N (Fig. 2) were plotted with ArcGIS 10.5 software (Esri Inc., USA).

## Supplementary information

Supplementary Information

## Data Availability

The data underlying the findings of this study are provided in the Source Data file. Source data are provided with this paper.

## Source data

Source Data

## Code availability

The SPSS package can be downloaded from https://www.ibm.com/products/spss-statistics. The Origin 2016 statistical package can be downloaded from https://www.originlab.com. The ArcGIS package can be downloaded from https://www.esri.com/en-us/arcgis/about-arcgis/overview. The source code for SIAR used in this paper is openly available from https://rdrr.io/cran/siar.
