# Supplementary material for: Significant contributions of combustion-related sources to ammonia emissions
Source: Nat Commun. 2022 Dec 13;13:7710. doi: 10.1038/s41467-022-35381-4 (PMC9747788; doi:10.1038/s41467-022-35381-4)
Supplement: Supplementary file 1 — Supplementary Information [file 41467_2022_35381_MOESM1_ESM.pdf]

# Supplementary Information for

## Significant contributions of combustion-related sources to ammonia emissions

Zhi-Li Chen[1]†, Wei Song[1]†, Chao-Chen Hu[1], Xue-Jun Liu[2], Guan-Yi Chen[3], Wendell W. Walters[4], Greg Michalski[5], Cong-Qiang Liu[1], David Fowler[6], Xue-Yan Liu[1]*

[1]School of Earth System Science, Tianjin University, Tianjin, 300072, China.

[2]College of Resources and Environmental Sciences, China Agricultural University, Beijing, 100193, China.

[3]School of Environmental Science and Engineering, Tianjin University, Tianjin 300072, China.

[4]Institute at Brown for Environment and Society, Brown University, 85 Waterman St, Providence, RI, 02912, United States.

[5]Department of Earth, Atmospheric, and Planetary Sciences, Purdue University, 550 Stadium Mall Drive, West Lafayette, IN 47907, United States.

[6]Centre for Ecology and Hydrology, Bush Estate, Penicuik, Midlothian, EH26 0QB, United Kingdom.

†These authors contributed equally to this work.

*Author for correspondence (email: liuxueyan@tju.edu.cn).

**This PDF file includes:**

**(1) Supplementary Figures 1 to 16**

**(2) Supplementary Tables 1 to 5**

**(3) Supplementary Note 1:**

1) Supplementary references 84–101 in Supplementary Text 1 are publications with the $\delta^{15}N_{a\text{-}NH3}$ observations.
2) Supplementary references 102–144 in Supplementary Text 2 are publications with $\delta^{15}N_{w\text{-}NH4+}$ observations.
3) Supplementary references 145–172 in Supplementary Text 3 are publications with $\delta^{15}N_{p\text{-}NH4+}$ observations.
4) Supplementary references 173–279 in Supplementary Text 4 are publications with simultaneous $C_{a\text{-}NH3}$ and $C_{p\text{-}NH4+}$ observations.

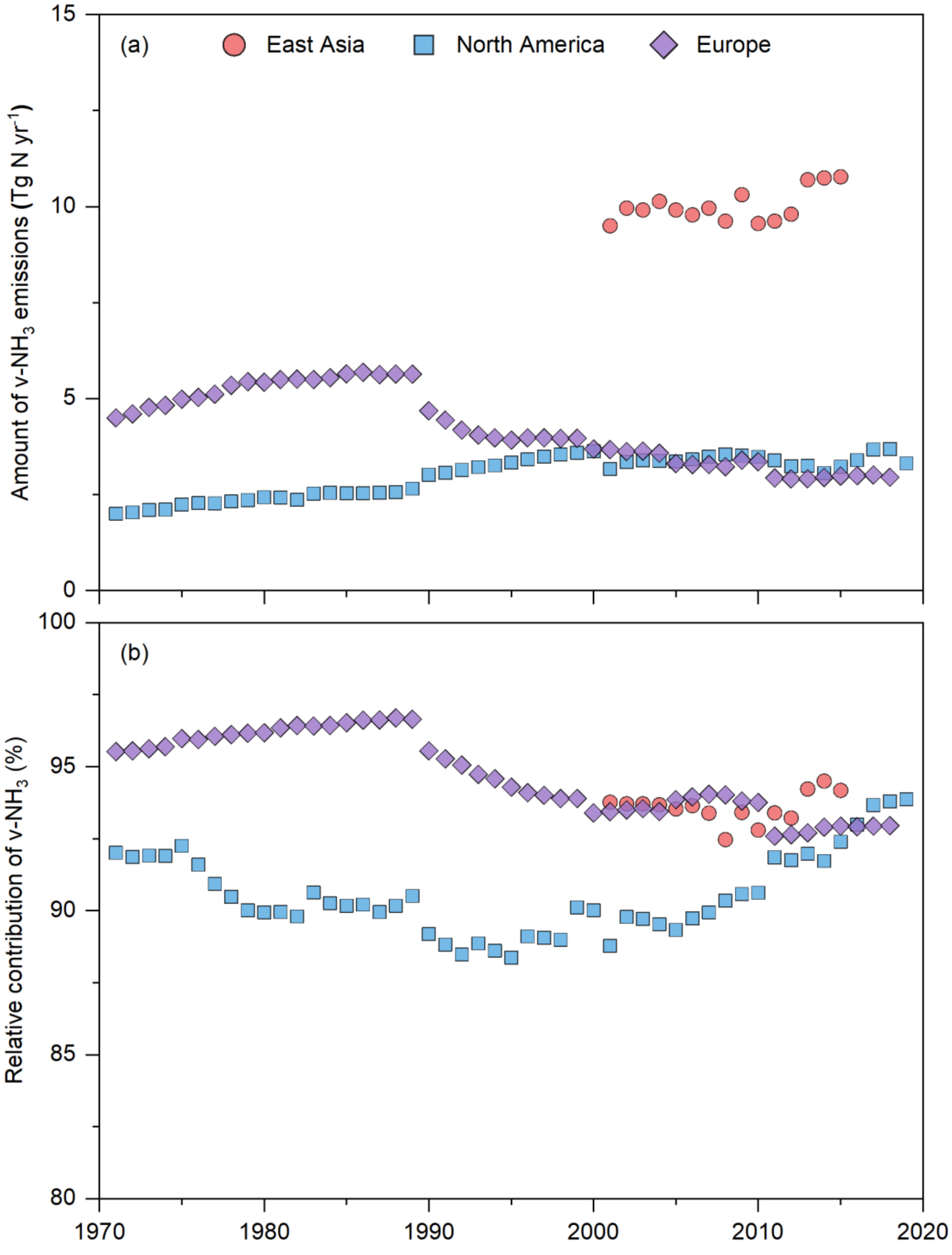


**Supplementary Fig. 1. Emission amounts (a) and relative contributions (b) of volatilization $NH_3$ (v-$NH_3$) in emission inventories of East Asia, North America, and Europe.** Mean annual values of available data in emission inventories are shown, and data sources are detailed in Supplementary Table 1.

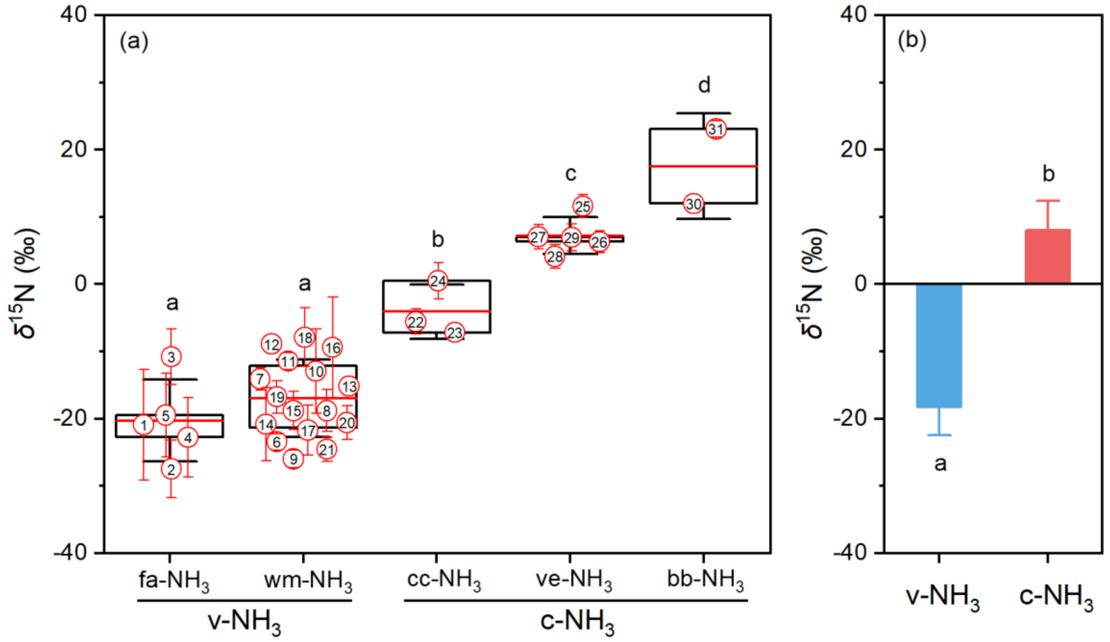


**Supplementary Fig. 2. $\delta^{15}$N of five major $NH_3$ sources (a) and emission inventories weighted v-$NH_3$ and c-$NH_3$ sources (b).** v-$NH_3$ represents volatilization $NH_3$; c-$NH_3$ represents combustion-related $NH_3$; fa-$NH_3$, wm-$NH_3$, cc-$NH_3$, ve-$NH_3$, and bb-$NH_3$ represent $NH_3$ emissions from fertilizer application, waste materials, coal combustion, vehicle exhausts, and biomass burning, respectively. In sub-figure a, each box encompasses the 25$^{th}$–75$^{th}$ percentiles, whiskers and the red line in each box are the SD and mean values, respectively. In sub-figure b, emission inventories weighted mean±SD values of v-$NH_3$ and c-$NH_3$ sources are shown. Calculations were detailed in the Methods. Values with different letters (a, b, c, and d) differ significantly at $p<0.05$. The $\delta^{15}$N values based on the passive samplers have been calibrated by adding 15‰[1–3]. The white-filled circles are mean $\delta^{15}$N values of $NH_3$ emissions from specific sources measured in different studies. The error bars and the numbers in the circles are SD values and data sources.

1: Fertilizer in the field, passive sampler (denoted as P)[4];
2: Urea-ammonia-nitrate fertilizer in the field, P[5];
3: Urea in the field, active sampler (denoted as A)[6];
4: Urea in the lab, P[7];
5: Fertilizer in the green land, A[8];
6: Human excreta, P[9];
7: Pig wastes, P[9];
8: Solid wastes, P[9];
9: Wastewater, P[9];
10: Cow wastes, P[5];
11: Cow wastes, A[10];
12: Chicken wastes, A[11];
13: Sheep wastes, A[11];
14: Dairy cow wastes, A[12];
15: Cow wastes, P[13];
16: Cow wastes, A[14];
17: Human excreta, A[8];
18: Cow wastes, A[8];
19: Chicken wastes, A[8];

20: Human excreta, A[15];
21: Pig farm and dairy farm, A[6];
22: Brown coal combustion, A[10];
23: Hard coal combustion, A[10];
24: Household coal combustion, A[16];
25: Vehicle exhaust sampled in a tunnel, P[5];
26: Vehicle exhaust sampled in a tunnel, A[17];
27: Vehicle exhaust sampled at a roadside, A[3];
28: Vehicle exhaust sampled in a tunnel, A[3];
29: Vehicle exhaust sampled on the road, A[3];
30: Biomass burning estimated by aerosol $NH_4^+$ in winter at Yurihonjo, Japan[18];
31: Biomass burning estimated by aerosol $NH_4^+$ in autumn at Nanchang, China[19].

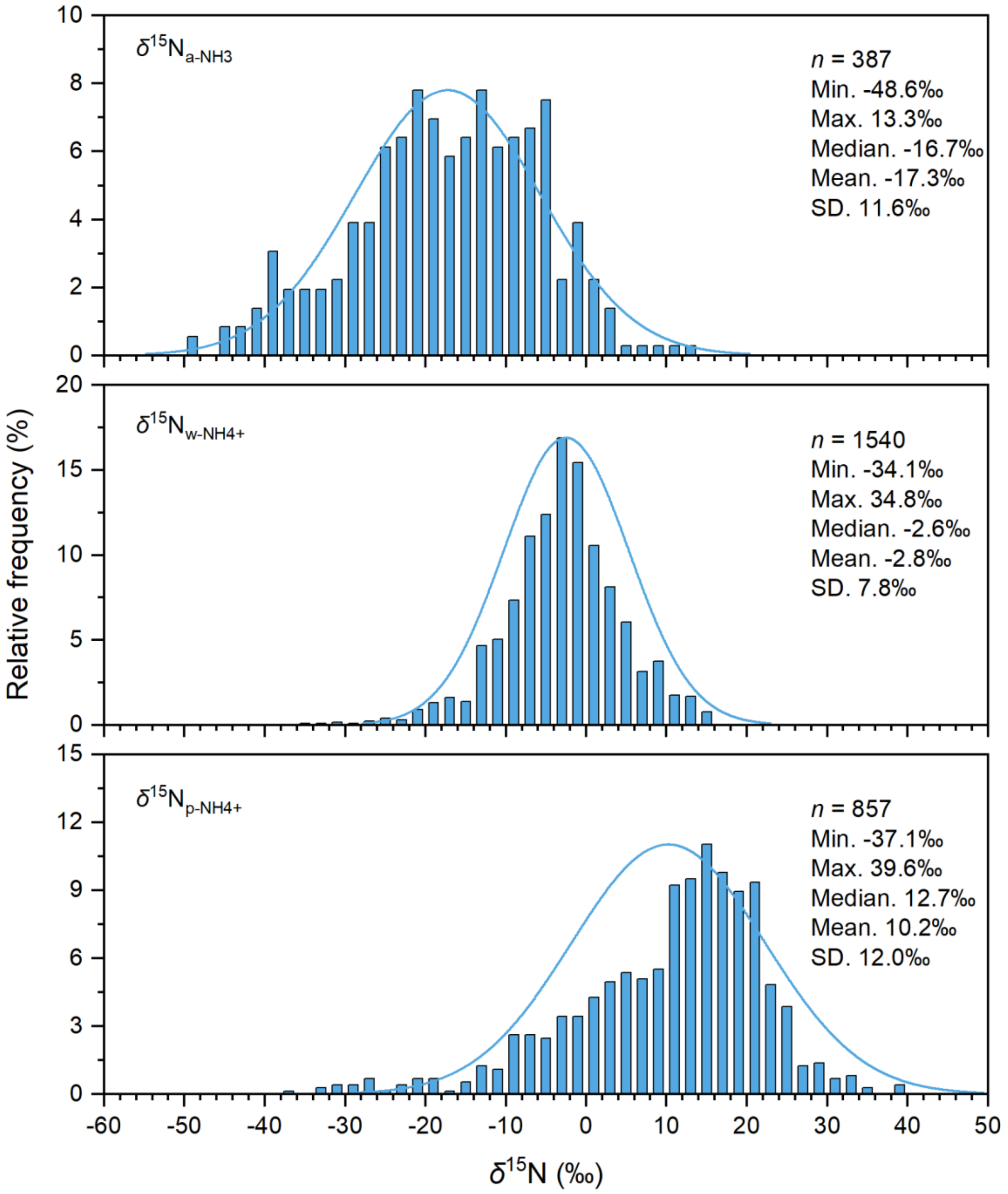


**Supplementary Fig. 3. Relative frequency histograms of $\delta^{15}N_{a\text{-}NH3}$, $\delta^{15}N_{w\text{-}NH4+}$, and $\delta^{15}N_{p\text{-}NH4+}$.** Replicate measurements (*n*) at all sites (Fig. 2) were used. The $\delta^{15}N_{a\text{-}NH3}$ data sources are listed in Supplementary Text 1. The $\delta^{15}N_{w\text{-}NH4+}$ data sources are listed in Supplementary Text 2. The $\delta^{15}N_{p\text{-}NH4+}$ data sources are listed in Supplementary Text 3.

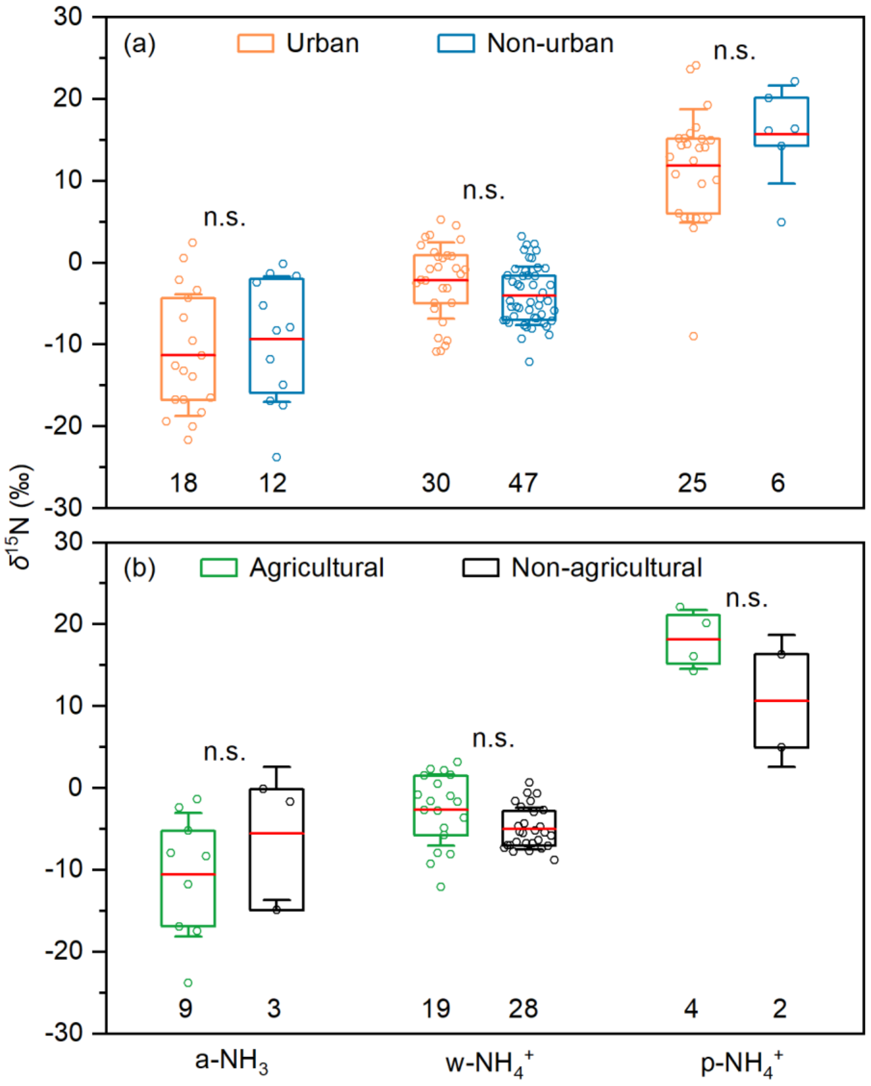


**Supplementary Fig. 4. $\delta^{15}$N of a-$NH_3$, w-$NH_4^+$, and p-$NH_4^+$ at urban and non-urban sites (a) and agricultural and non-agricultural sites (b).** Circles around each box show mean values of replicate measurements at each site (6–36 and 2–20 for a-$NH_3$ at urban and non-urban sites, respectively; 1–61 and 1–156 for w-$NH_4^+$ at urban and non-urban sites, respectively; 1–169 and 1–84 for p-$NH_4^+$ at urban and non-urban sites, respectively). In sub-figure b, non-urban sites are further divided into agricultural and non-agricultural sites (2–20 and 7–12 for a-$NH_3$ at agricultural and non-agricultural sites, respectively; 1–156 and 1–137 for w-$NH_4^+$ at agricultural and non-agricultural sites, respectively; 1–77 and 5–84 for p-$NH_4^+$ at agricultural and non-agricultural sites, respectively). $\delta^{15}N_{a\text{-}NH3}$ based on the passive samplers has been calibrated by adding 15‰[1–3]. Each box encompasses the 25$^{th}$–75$^{th}$ percentiles, whiskers and the red line in each box are the SD and mean values, respectively. The numbers below the boxes are those of observation sites. n.s. indicates no significant differences in $\delta^{15}$N between urban and non-urban or agricultural and non-agricultural sites at $p$<0.05.

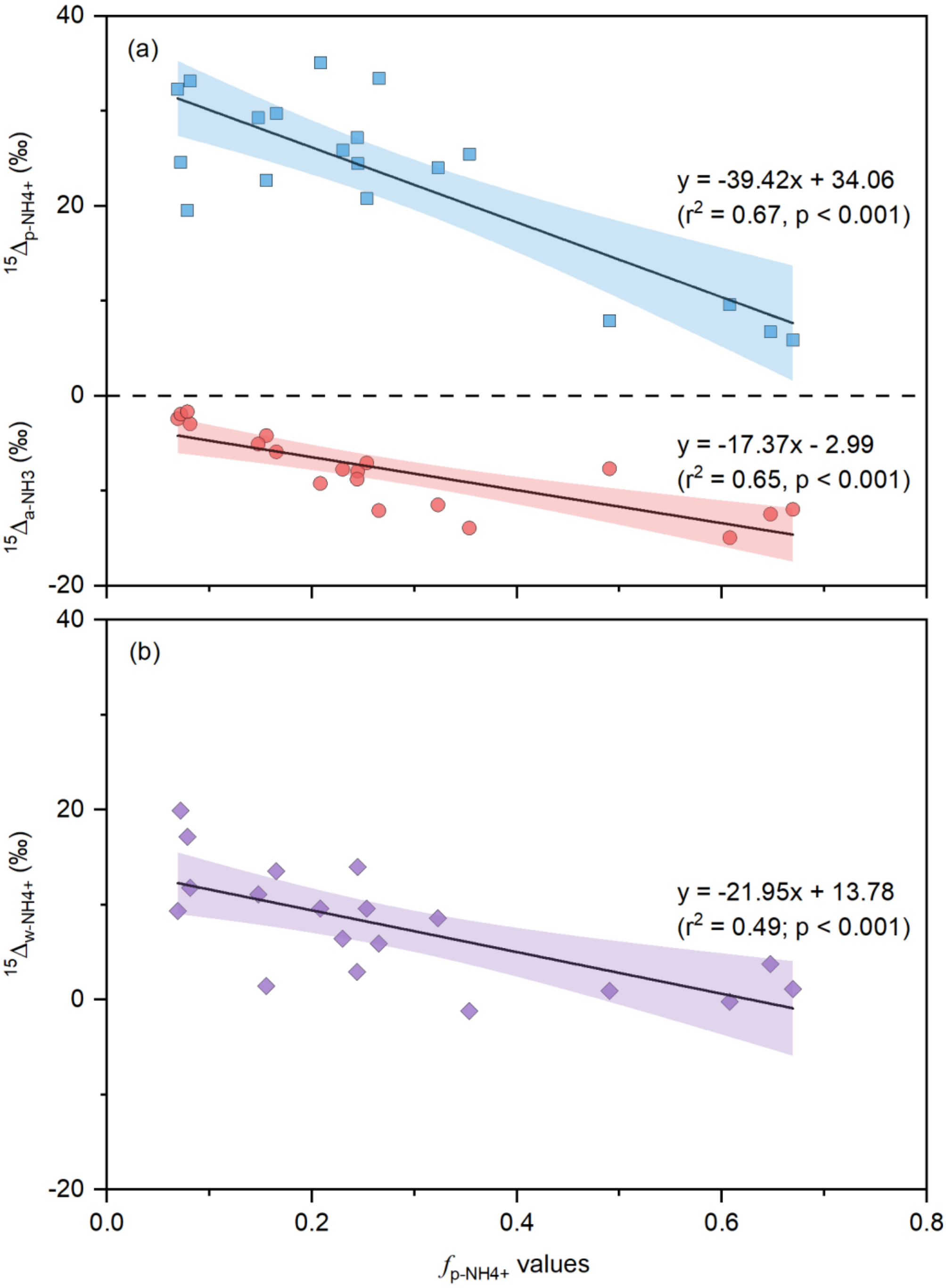


**Supplementary Fig. 5. Correlation of $^{15}\Delta_{a\text{-}NH3}$, $^{15}\Delta_{p\text{-}NH4+}$, and $^{15}\Delta_{w\text{-}NH4+}$ with $f_{p\text{-}NH4+}$ (i.e., $C_{p\text{-}NH4+}/(C_{a\text{-}NH3}+C_{p\text{-}NH4+})$) in the atmosphere.** Simultaneous observation data of seasonal mean $C_{a\text{-}NH3}$, $C_{p\text{-}NH4+}$, and corresponding $^{15}\Delta_{a\text{-}NH3}$, $^{15}\Delta_{p\text{-}NH4+}$, and $^{15}\Delta_{w\text{-}NH4+}$ ($n$=19 for each) at six sites (Supplementary Table 5) are used. The shade is the 95% confidence interval. Calculations of $^{15}\Delta_{a\text{-}NH3}$, $^{15}\Delta_{p\text{-}NH4+}$, and $^{15}\Delta_{w\text{-}NH4+}$ are detailed in Methods.

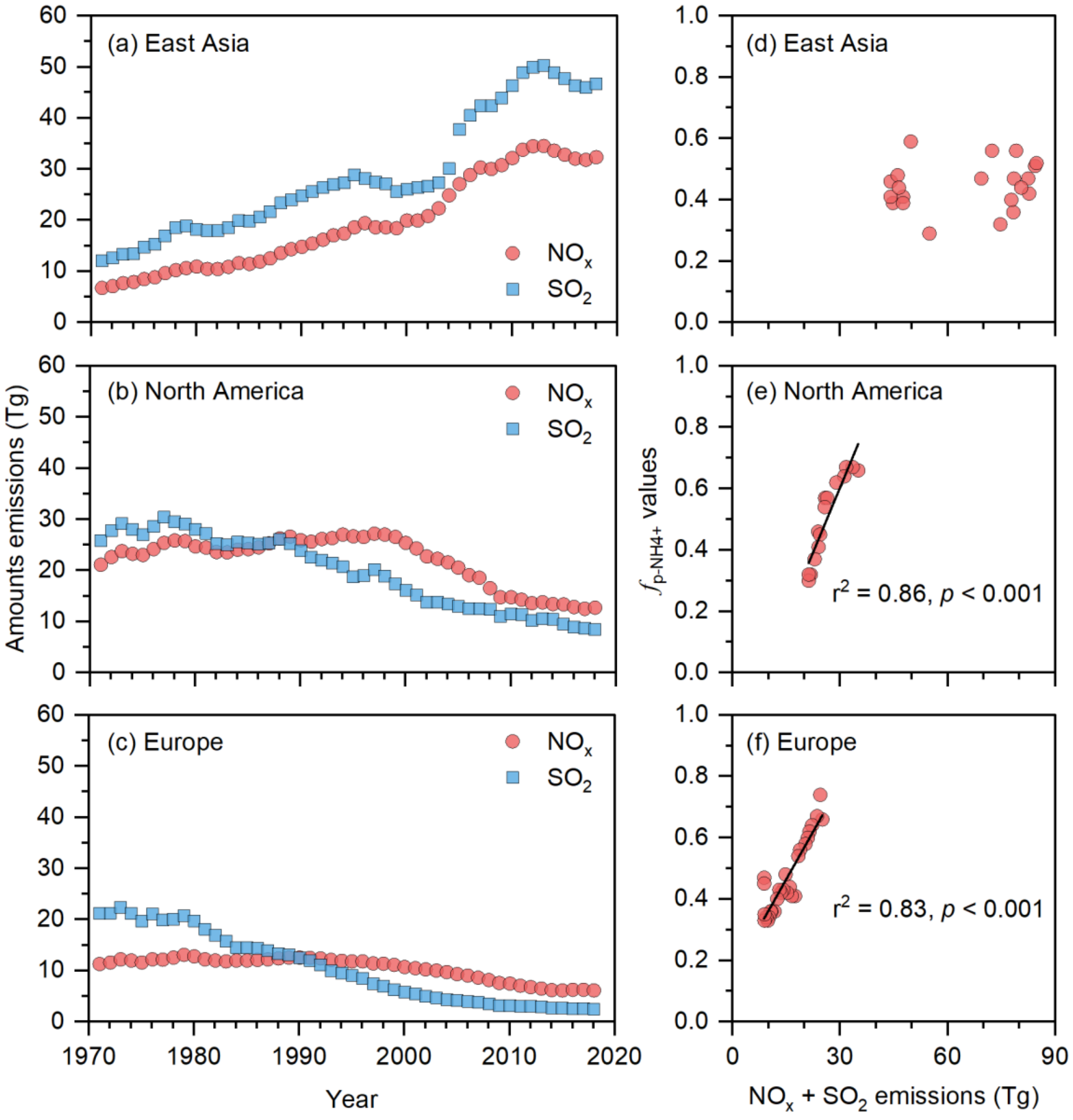


**Supplementary Fig. 6. Emission amounts of $NO_x$ and $SO_2$ (a, b, and c) and correlation between $f_{p\text{-}NH4+}$ and the emission of $NO_x$ and $SO_2$ in East Asia (d), North America (e), and Europe (f).** Data on $NO_x$ and $SO_2$ emissions were downloaded from[4]. The annual mean $f_{p\text{-}NH4+}$ values in East Asia during 1993–2018, North America during 2004–2018, and Europe during 1990–2017 (detailed in Supplementary Fig. 7) were used. East Asia includes China, Japan, and Korea. North America includes both USA and Canada. Europe includes Austria, Belgium, Denmark, Faroe Islands, Finland, France, Germany, Gibraltar, Greece, Greenland, Iceland, Italy, Ireland, Luxembourg, Norway, Netherlands, Portugal, Spain, Sweden, Switzerland, and United Kingdom.

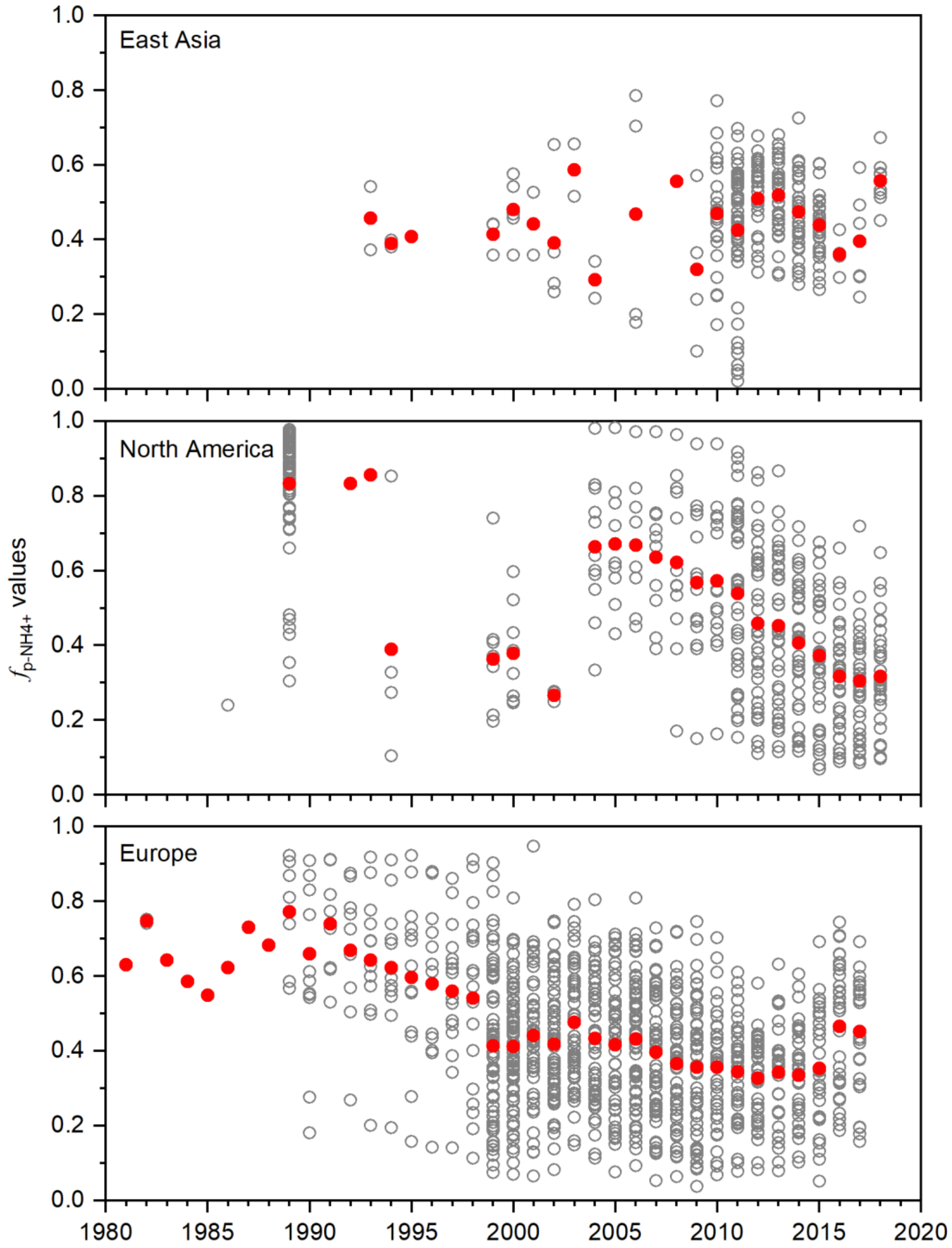


**Supplementary Fig. 7. Time series of $f_{p\text{-}NH4+}$ (i.e., $C_{p\text{-}NH4+}/(C_{a\text{-}NH3}+C_{p\text{-}NH4+})$) in the atmosphere.** Grey circles are site-based mean values of replicate measurements ($n$=1–55 in East Asia, $n$=1–59 in North America, $n$=1–68 in Europe) in each region. The red-filled circle is the mean value of site-based observation values (Gray circles) available for the corresponding year. The data sources (references) in the figure are listed in Supplementary Text 4.

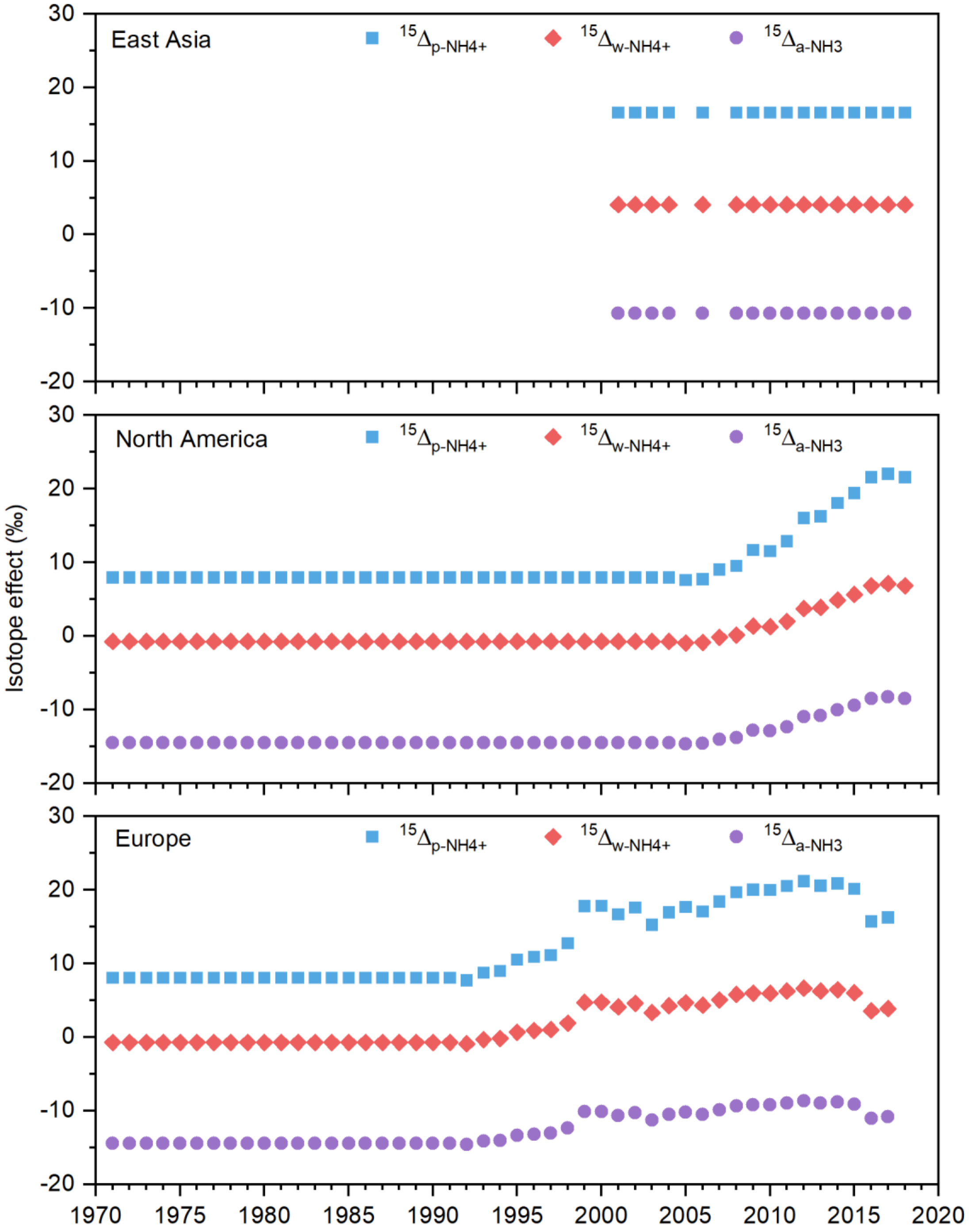


**Supplementary Fig. 8. Annual mean $^{15}\Delta_{a\text{-}NH3}$, $^{15}\Delta_{w\text{-}NH4+}$, and $^{15}\Delta_{p\text{-}NH4+}$ in each region.** Calculations are detailed in Methods.

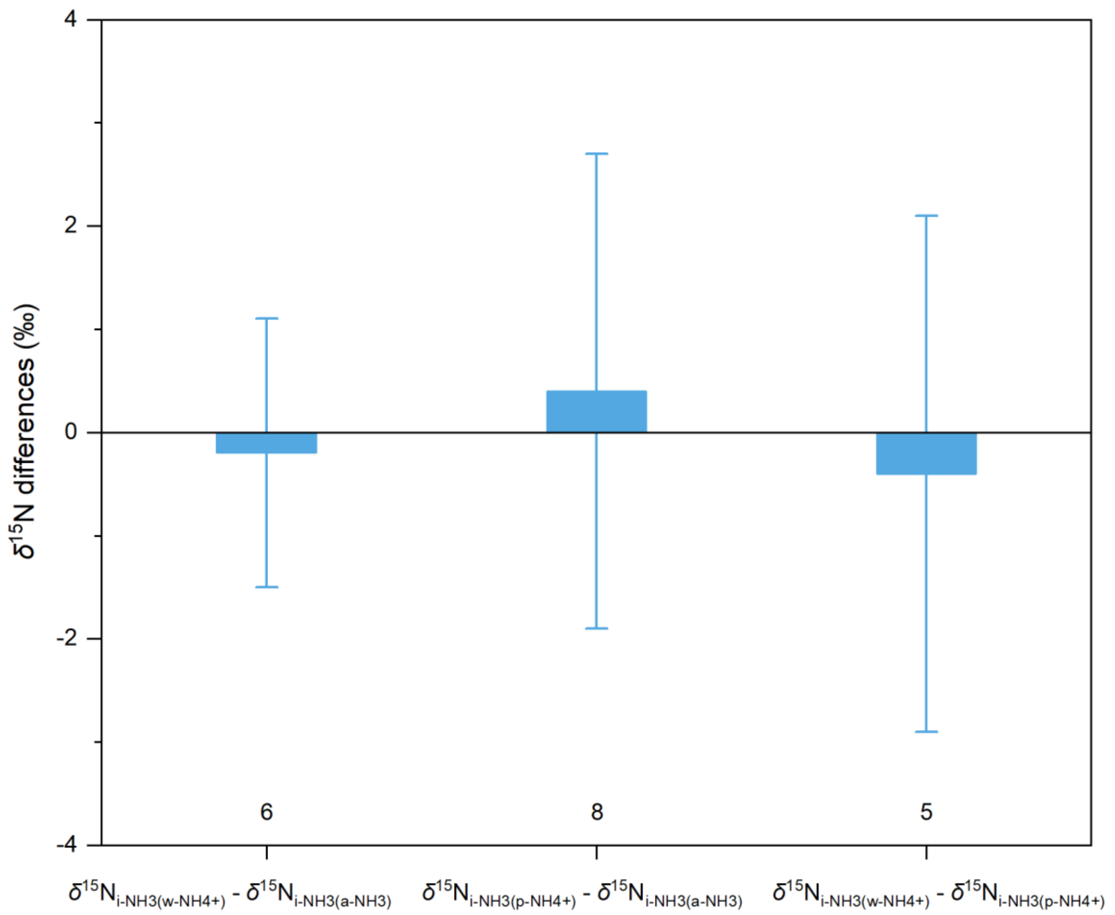


**Supplementary Fig. 9. Differences between the paired $\delta^{15}N_{i\text{-}NH3(a\text{-}NH3)}$ and $\delta^{15}N_{i\text{-}NH3(w\text{-}NH4+)}$ ($\delta^{15}N_{i\text{-}NH3(w\text{-}NH4+)}$ - $\delta^{15}N_{i\text{-}NH3(a\text{-}NH3)}$), $\delta^{15}N_{i\text{-}NH3(a\text{-}NH3)}$ and $\delta^{15}N_{i\text{-}NH3(p\text{-}NH4+)}$ ($\delta^{15}N_{i\text{-}NH3(p\text{-}NH4+)}$ - $\delta^{15}N_{i\text{-}NH3(a\text{-}NH3)}$), $\delta^{15}N_{i\text{-}NH3(p\text{-}NH4+)}$ and $\delta^{15}N_{i\text{-}NH3(w\text{-}NH4+)}$ ($\delta^{15}N_{i\text{-}NH3(w\text{-}NH4+)}$ - $\delta^{15}N_{i\text{-}NH3(p\text{-}NH4+)}$).** Error bars represent standard error. Numbers below the bars are observation sites (detailed in Supplementary Table 4). Calculations were based on simultaneous observations on either $\delta^{15}N_{a\text{-}NH3}$ and $\delta^{15}N_{p\text{-}NH4+}$, $\delta^{15}N_{a\text{-}NH3}$ and $\delta^{15}N_{w\text{-}NH4+}$, $\delta^{15}N_{p\text{-}NH4+}$ and $\delta^{15}N_{w\text{-}NH4+}$ (detailed in Methods).

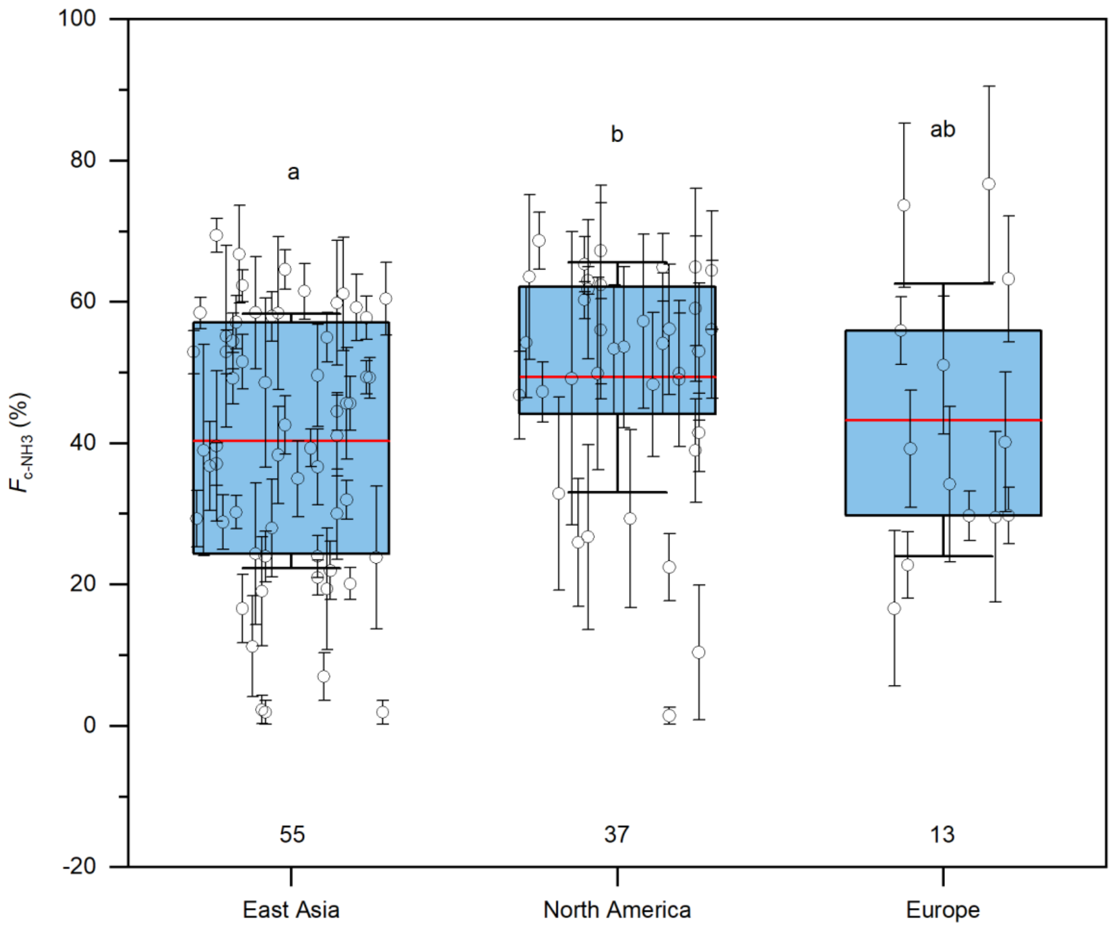


**Supplementary Fig. 10. $F_{\text{c-NH3}}$ values in East Asia, North America, and Europe.** Circles and error bars around each box show mean±SD of $F_{\text{c-NH3}}$ at each site. Each box encompasses the 25$^{\text{th}}$–75$^{\text{th}}$ percentiles, whiskers and the red line in each box are SD and mean values, respectively. The numbers below the boxes represent the numbers of observation sites in each region. Different letters (a and b) above the boxes indicate a significant difference ($p$<0.05) among the three regions. Calculations were detailed in Methods.

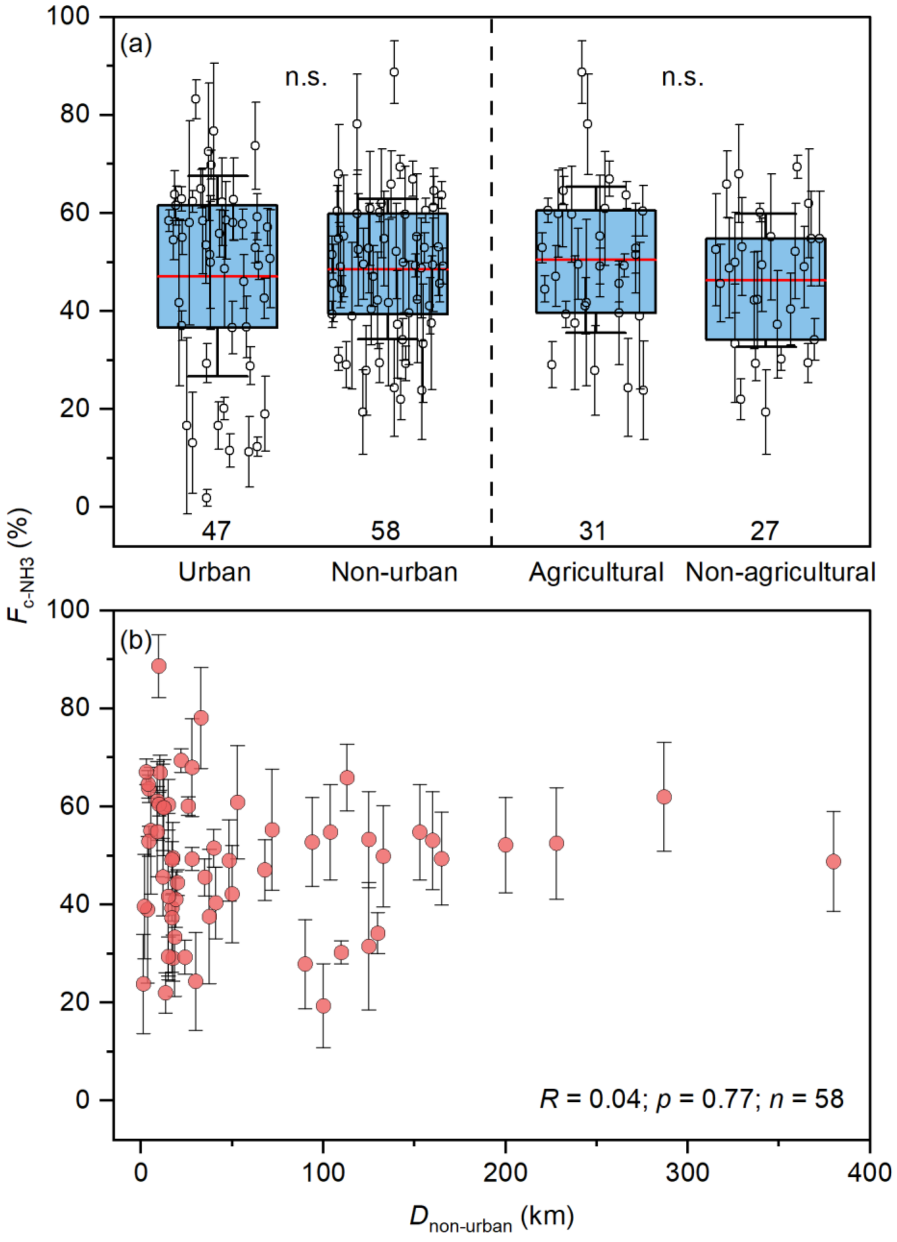


**Supplementary Fig. 11. (a) Mean $F_{c\text{-}NH3}$ of urban, non-urban, agricultural, and non-agricultural sites, and (b) variations of $F_{c\text{-}NH3}$ at non-urban sites with the corresponding distances from the edge of the nearest urban area ($D_{non\text{-}urban}$).** Error bars represent SD. In sub-figure a, each box encompasses the 25th–75th percentiles, whiskers and the red line in each box are SD and mean values, respectively. Numbers below the boxes are the numbers of observation sites. n.s.: no significance. In sub-figure b, the $D_{non\text{-}urban}$ values were measured by using the coordinate information and Google Earth (https://earth.google.com).

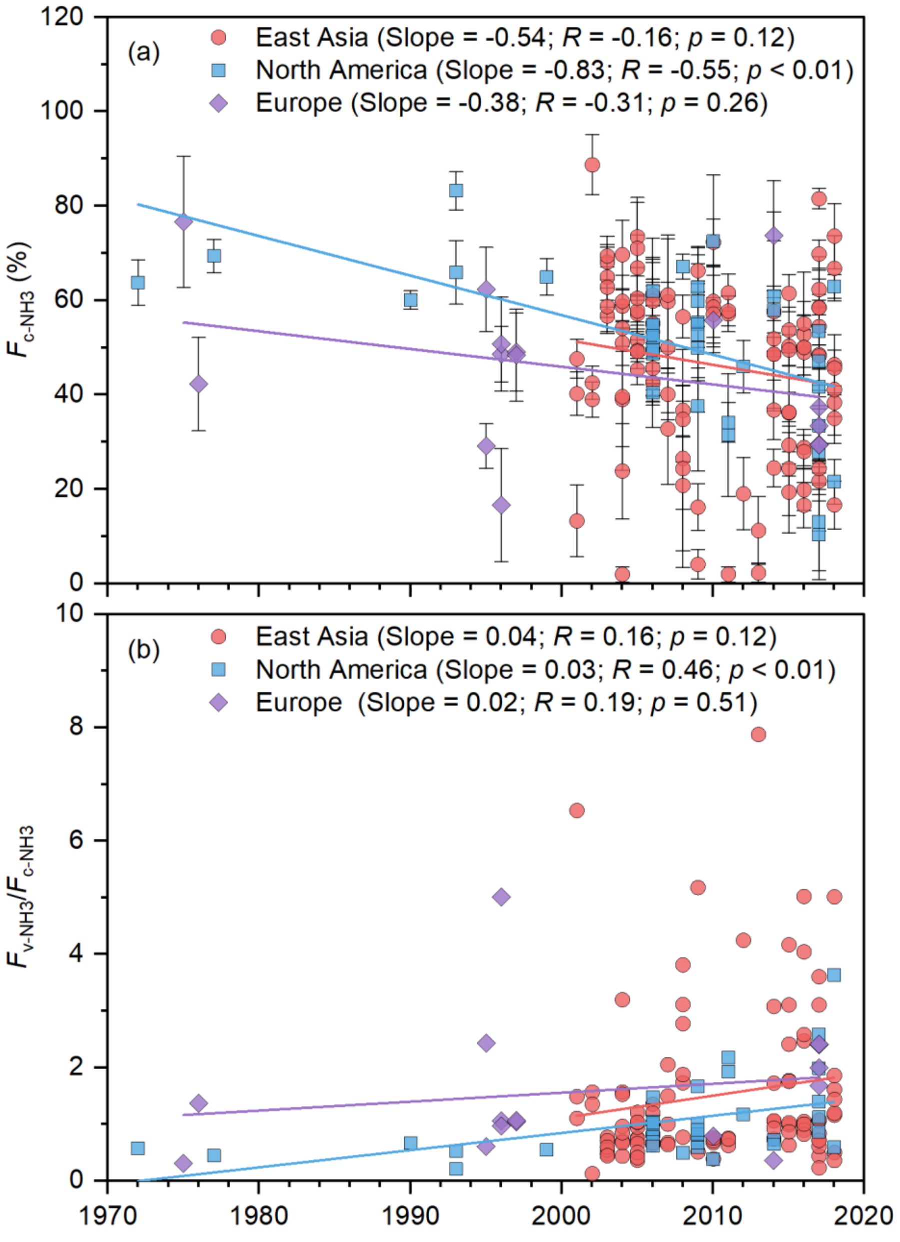


**Supplementary Fig. 12. Relative contributions of c-$NH_3$ (a) and the ratio of $F_{v\text{-}NH3}$ to $F_{c\text{-}NH3}$ (b) in East Asia, North America, and Europe.** Error bars represent SD. Calculations were detailed in the Methods.

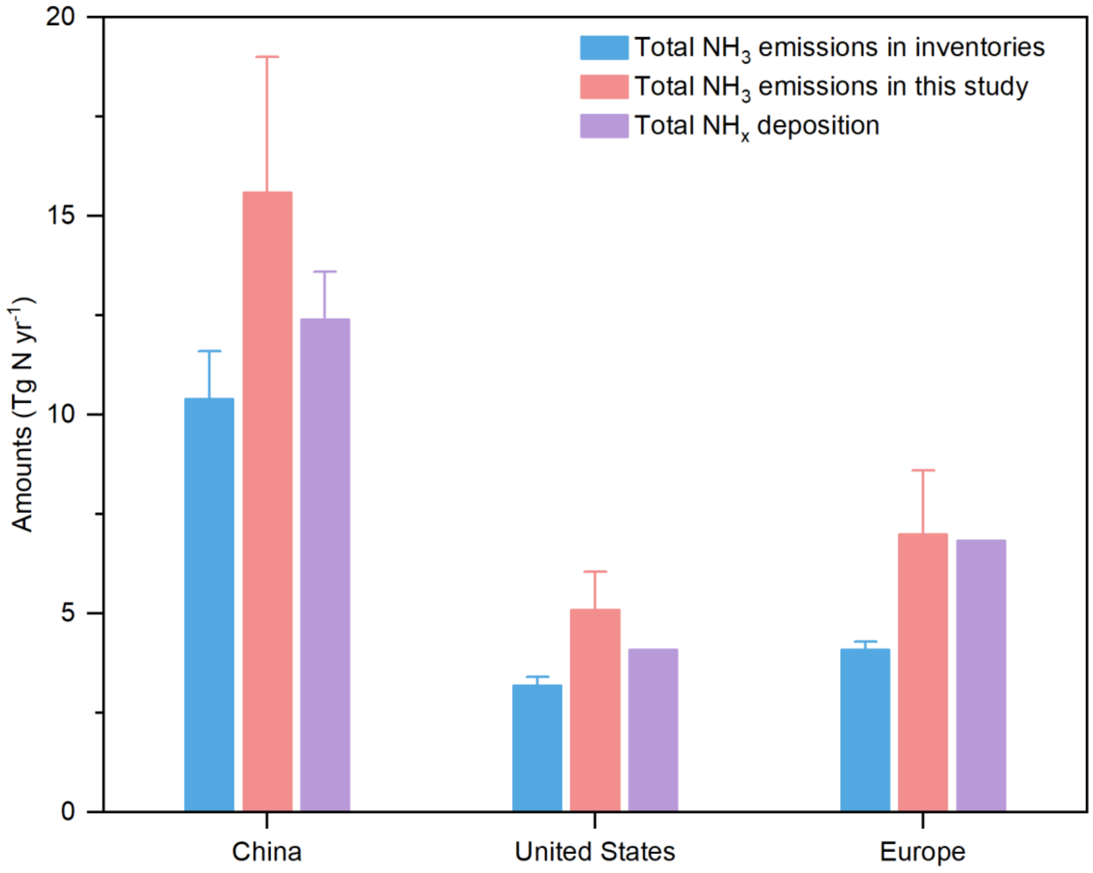


**Supplementary Fig. 13. Total amounts of $NH_3$ emissions and $NH_x$ deposition in China, the United States, and Europe.** Error bars represent SD. Data on total $NH_3$ emissions in inventories were cited from Supplementary Table 1. Calculations of total $NH_3$ emission amounts were detailed in the Methods. Data of the $NH_x$ deposition (i.e., the sum of a-$NH_3$, p-$NH_4^+$, and w-$NH_4^+$ deposition) were cited from refs. 21–26.

170 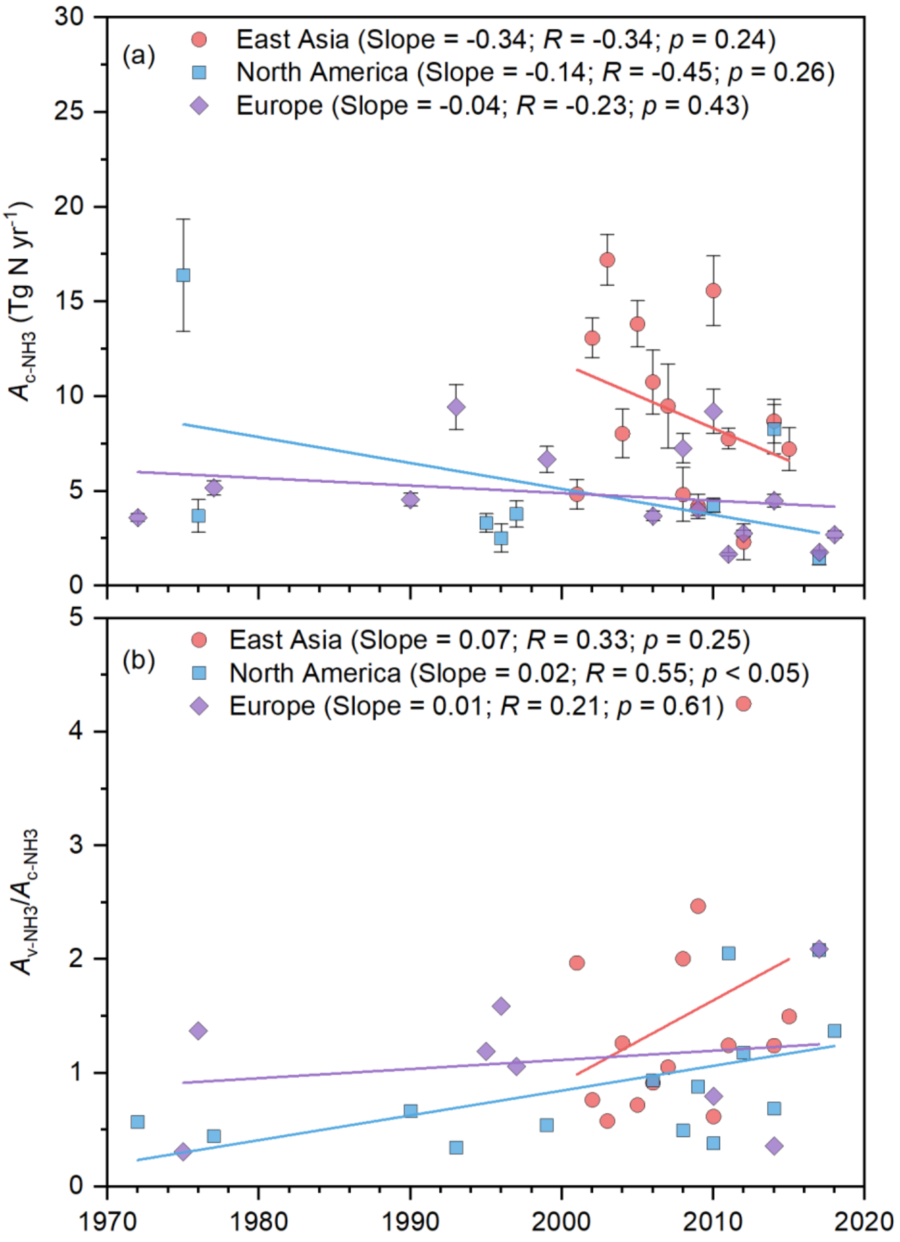


171 **Supplementary Fig. 14. Amounts of c-$NH_3$ emission (a) and the ratio of $A_{v\text{-}NH3}$ to**
172 **$A_{c\text{-}NH3}$ (b) in East Asia, North America, and Europe.** Error bars represent SD. The
173 calculations and data sources were detailed in the Methods.

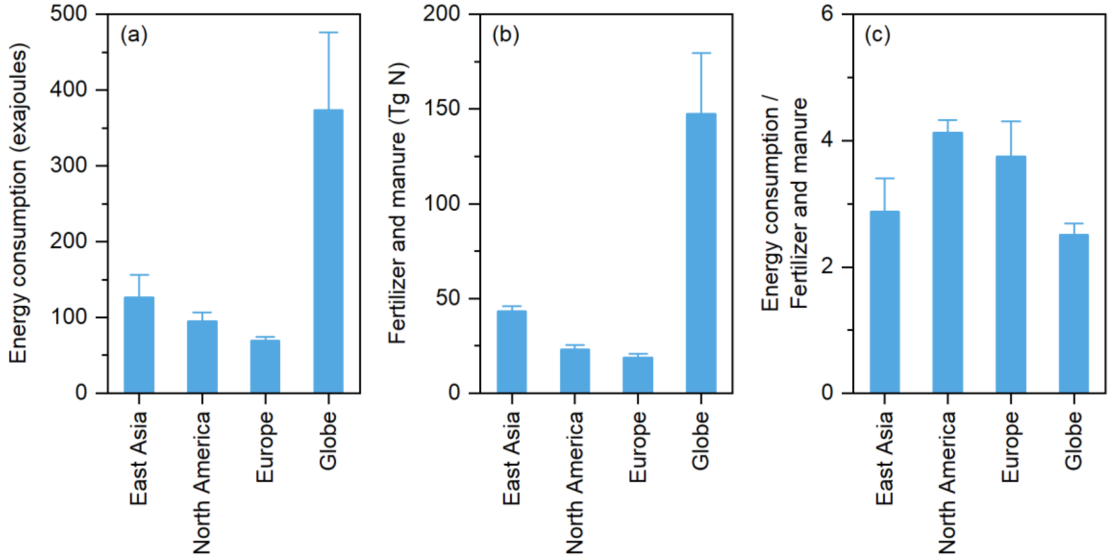


**Supplementary Fig. 15. The spatial difference in amounts of energy consumption (a), fertilizer consumption and animal manure (b), and the ratio of energy consumption to fertilizer consumption and animal manure (c) in East Asia during 2001–2017, North America, Europe, and the Globe during 1971–2017.** Error bars represent SD. East Asia includes China, Japan, and Korea. North America includes both USA and Canada. Europe includes Austria, Belgium, Bulgaria, Croatia, Cyprus, Czechia, Denmark, Estonia, Finland, France, Germany, Greece, Hungary, Ireland, Italy, Latvia, Lithuania, and Luxembourg. Data on energy consumption were downloaded from[27]. Data on fertilizer consumption and animal manure were downloaded from[28].

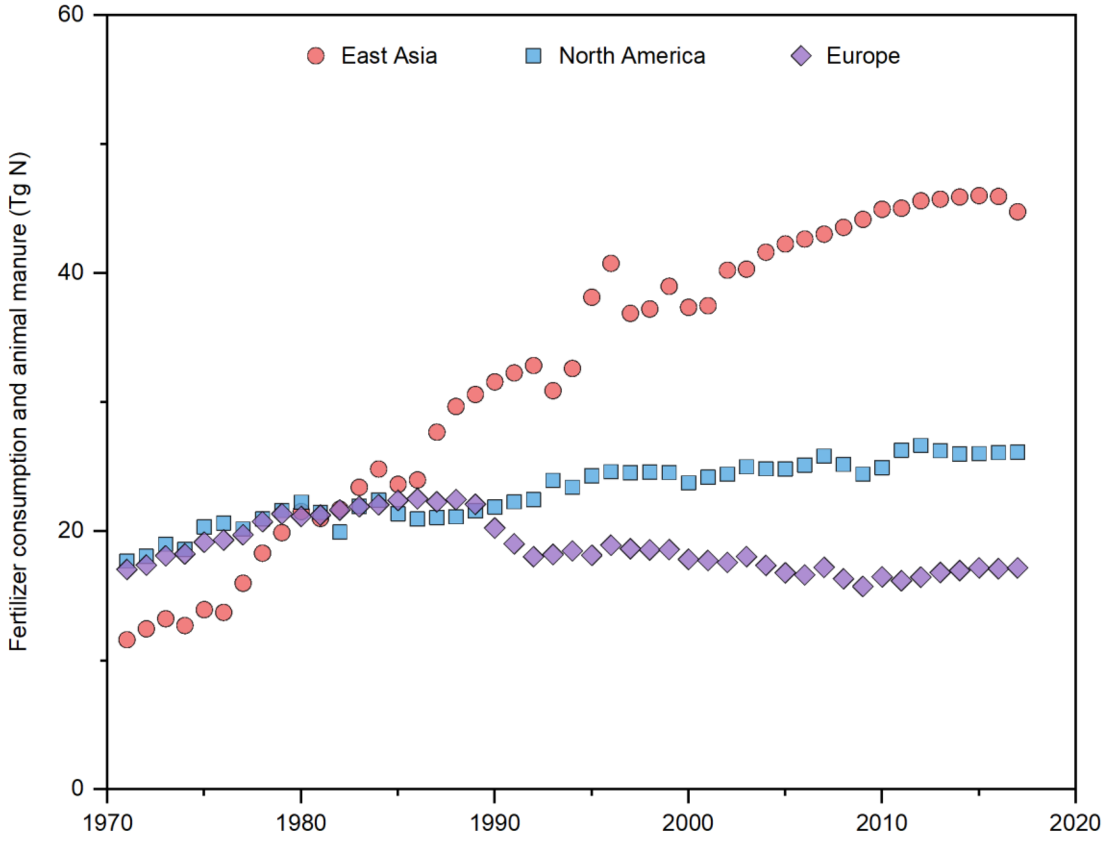


**Supplementary Fig. 16. Time series of fertilizer consumption and animal manure in East Asia, North America, and Europe.** East Asia includes China, Japan, and Korea. North America includes both USA and Canada. Europe includes Austria, Belgium, Bulgaria, Croatia, Cyprus, Czechia, Denmark, Estonia, Finland, France, Germany, Greece, Hungary, Ireland, Italy, Latvia, Lithuania, Luxembourg, Malta, Netherlands, Poland, Portugal, Romania, Slovakia, Slovenia, Spain, Sweden, and United Kingdom. Data were downloaded from[28,29]. The amount excreted in the manure of Europe excludes Malta, Netherlands, Poland, Portugal, Romania, Slovakia, Slovenia, Spain, Sweden, and the United Kingdom due to the lack of statistical data.

**Supplementary Table 1. Sources of volatilization $NH_3$ (v-$NH_3$) and combustion-related $NH_3$ (c-$NH_3$) in emission inventories.**

| Regions / Countries | v-$NH_3$ sources | c-$NH_3$ sources | Refs. |
|---|---|---|---|
| East Asia, North America, Europe | Manure management, manure in pasture or range or paddock, direct soil emissions, other indirect soil emissions, wastewater handling, and other waste handling | Public electricity and heat production, manufacturing industries and construction, other energy industries, road transportation, rail transportation, inland navigation, other transportation, residential and other sectors, fugitive emissions from solid fuels, production of other minerals, production of chemicals, agricultural waste burning, waste incineration, and other energy industries | 20 |
| East Asia | Non-combustion sources | Primary coal, secondary coal, natural gas, other gas fuels, light oil fuels, diesel oil, heavy oil fuels, biofuel, other fuels, and cement kilns (only for Japan) combustion | 30 |
| East Asia | Agriculture | Power, industry, transportation, residential | 31 |
| East Asia | Fertilizer application, manure management, human perspiration and respiration, and latrines | Combustion | 32 |
| Europe | Manure management and agriculture other | Public power, industry combustion, other station combustion, waste, public electricity and heat production, industry combustion, other stationary combustion, road transport, waste combustion, manure management | 33 |
| Europe | Agriculture | Energy supply, extractive industry, manufacturing and extractive industry, residential, commercial and institutional, transport, waste, and other | 34 |
| Europe | Manure, fertilizer, and oceanic sources | Biofuel, Transportation, industry, energy, and open-fire combustion | 35 |
| USA | Miscellaneous | Fuel combustion electric generating utility, fuel combustion industrial and other, chemical and allied product mfg, metals processing, petroleum & related industries, other industrial processes, solvent utilization, storage and transport, waste disposal and recycling, highway vehicles, and off-highway vehicles | 36 |

| **Regions / Countries** | **v-$NH_3$ sources** | **c-$NH_3$ sources** | **Refs.** |
|---|---|---|---|
| Canada | Animal production and crop production for Canada | Ore and mineral industries, oil and gas industry, electric power generation utilities, manufacturing, transportation and mobile equipment, agriculture-fuel use, commercial or residential or institutional, incineration and waste, paints and solvents, dust and fires | 37 |
| China | Synthetic fertilizer, agricultural soil, N-fixing crop, compost, livestock, human excrement, and waste disposal | Biomass burning, chemical industry, traffic, and $NH_3$ escape | 38 |
| China | Synthetic fertilizer, agricultural soil, N-fixing crop, compost, livestock, human excrement, and waste disposal | Biomass burning, chemical industry, traffic, and $NH_3$ escape | 39 |
| China | Cropland, livestock, grassland, aquaculture, waste disposal, humans, urban green land, and pets | Biomass burning, fuel combustion, chemical industry, and traffic sources | 40 |
| China | Agriculture | Power, industry, residential, transportation, solvent use | 41 |
| China | Fertilizer application and livestock wastes | Human and others | 42 |

Note: East Asia includes China, Japan, and Korea. North America includes USA and Canada. Europe includes Austria, Belgium, Bulgaria, Croatia, Cyprus, Czechia, Denmark, Estonia, Finland, France, Germany, Greece, Hungary, Ireland, Italy, Latvia, Lithuania, Luxembourg, Malta, Netherlands, Poland, Portugal, Romania, Slovakia, Slovenia, Spain, Sweden, and United Kingdom.

**Supplementary Table 2. Direct measurements on c-$NH_3$ and their influences on ambient $NH_3$.** n.a. refers to 'not available'.

| **Methods** | **Scales** | **Sources** | **Data** | **Notes** | **Refs.** |
|---|---|---|---|---|---|
| Laboratory simulations | Laboratory | Biomass burning | 19.3% | Fraction of $NH_3$ in total reactive N emitted during biomass burning. $NH_3$ is the most important active nitrogen emission after nitric oxide during biomass burning. | 43 |
| Laboratory simulations | Laboratory | Vehicle exhausts | 10 and 21 mg $kg^{-1}$ | Emission factors of diesel light-duty vehicles equipped with selective catalytic reduction at 23℃ and -7℃, respectively, which were as high as those of gasoline light-duty vehicles and have long been overlooked by emission inventories. Diesel vehicles accounted for 52% of the total vehicle in Europe in 2015. | 44, 45 |
| Ground observations | Site (Colorado, USA) | Biomass burning | 20 times | Multiple $NH_3$ concentrations during wildfire smoke-impacted periods relative to the non-fire period | 46 |
| Ground observations | Site (South Korea) | Fossil-fuel combustion | 0.21–0.99 ppm | $NH_3$ concentrations emissions from bituminous coal power plants. $NH_3$ emission factor for bituminous coal power plants is 0.0029 kg $NH_3$ $ton^{-1}$, which is ten times that of US EPA (0.00028 kg $NH_3$ $ton^{-1}$) | 47 |
| Ground observations | Site (Shanghai, China) | Vehicle exhausts | 3 times | Multiple $NH_3$ concentrations in the tunnel relative to that at a nearby urban site, indicate strong vehicle $NH_3$ emissions in the tunnel | 48 |
| Ground observations | Site (California, USA) | Vehicle exhausts | 10 times | Multiple $NH_3$ concentrations in the tunnel exit relative to that in the tunnel entrance | 49 |
| Ground observations | Site (Shanghai, China) | Vehicle exhausts | 5 and 11 times | Multiple $NH_3$ concentrations in the tunnel exit relative to that in the tunnel entrance and the ambient air, respectively | 50 |

| Methods | Scales | Sources | Data | Notes | Refs. |
|---|---|---|---|---|---|
| Ground observations | Site (Eureka & Toronto, Canada) | Biomass burning | 2 times | The observed $NH_3$ column concentration at Eureka doubled during the period of fire-affected from the 2014 Northwest Territories fires | 51 |
| Ground observations | Site (Rome, Italy) | Vehicle exhausts | 5 times | Multiple $NH_3$ concentration at the traffic sites relative to that at urban background site, there is a strong correlation between $NH_3$ and CO concentration | 52 |
| Ground observations | Site (Shanghai, China) | c-$NH_3$ | 2 and 4 times | Multiple hourlies averaged $NH_3$ concentrations at the industrial site (19.6±8.2 ppb) in Shanghai relative to that at nearby rural (10.4±5.0 ppb) and urban (5.4±3.3 ppb) sites, respectively, during the same period. Remarkable high-frequency $NH_3$ variations were measured at the industrial site, with a concentration peak of 279.3 ppb and a highest hourly average of 84.9 ppb, indicating instantaneous nearby industrial emission peaks. | 53 |
| Ground observations | Site (China) | Miscellaneous | 2−3 times | Multiple $NH_3$ concentrations at urban sites relative to that at mountainous/forest/grassland/waterbody sites | 54 |
| Ground observations | Site (Worldwide) | Miscellaneous | 2 times | Multiple $NH_4^+$ deposition in urban areas relative to that in nearby rural areas | 55 |
| Ground observations | Site (New Jersey and California, USA) | Vehicle exhausts | 0.029±0.005 ppbv/ppbv | The mean values of the on-road $NH_3$:CO emission ratio, which was substantially higher than that in the National Emission Inventory (0.008-0.018 ppbv/ppbv) | 56 |
| Ground observations | City (Zurich, Tartu, Tallinn, Europe) | Vehicle exhausts | 37%–94% | The enhancement proportion of $NH_3$ concentration in the traffic areas in Zurich, Tartu, and Tallinn, three Europe cities, over the average background concentrations. | 57 |

| **Methods** | **Scales** | **Sources** | **Data** | **Notes** | **Refs.** |
|---|---|---|---|---|---|
| Aircraft observations | Regional (USA) | Biomass burning | 66% | Fraction of $NH_3$ in total reactive N emitted from wildfires. $NH_x$ emission factors in temperate forest fires in the US were about 2.5 times higher than the best estimated temperate forest emission factor used in models | 58 |
| Satellite observation | Global | Point sources | 4.6 Tg N $yr^{-1}$ | Total $NH_3$ emission, which is about ~2.5 times more than the current amount in the Hemispheric Transport Atmospheric Pollution version 2 (HTAPv2) emission inventory (2.1 Tg $yr^{-1}$) | 59 |
| Satellite observations & oversampling techniques | Global | c-$NH_3$ | 158 hotspots | A high-resolution map of atmospheric $NH_3$ showed the hotspots of c-$NH_3$ emissions, including burning coal mines and coal-related industries: coal mining, thermal power plants, coke production, and other chemical coal industries | 60 |
| Satellite observation & wind-adjusted super-resolution technique | Global | c-$NH_3$ | >500 | Amount of $NH_3$ point sources, including 266 industrial $NH_3$ hotspots and 13 urban $NH_3$ hotspots in African megacities | 61 |
| Satellite observation | Global | Biomass burning | n.a. | Biomass burning controls the seasonal surface $NH_3$ concentrations in the Southern Hemisphere with frequent fires, such as in Africa north of the Equator, Africa south of the Equator, and central South America, and also affect the temporal variation of surface $NH_3$ concentrations in high $NH_3$ concentration regions, such as China, USA, and Europe | 62 |

| **Methods** | **Scales** | **Sources** | **Data** | **Notes** | **Refs.** |
|---|---|---|---|---|---|
| Satellite observation | Regional (Alberta, Canada) | Biomass burning | 30 Gg | The satellite-measured $NH_3$ emissions during the Horse River fire, accounted for 20% of total anthropogenic emissions in Alberta, Canada | 63 |
| Satellite observation | Global | Biomass burning | n.a. | Some high-latitude regions during peak forest fire activity often have $NH_3$ concentrations approaching those in agricultural hotspots | 25 |
| Emission inventory | City (Shanghai, China) | Vehicle exhausts | 12% | Fraction of vehicle $NH_3$ emissions in total $NH_3$ emissions in Shanghai, China | 50 |
| Emission inventory | Regional (Pearl River Delta, China) | Vehicle exhausts | 8.1%–19% | Fraction of on-road vehicle $NH_3$ emissions in total $NH_3$ emissions in the Pearl River Delta of China, which increased from 8.1% in 2006 to 18.8% in 2012 due to the increase in vehicle population | 64 |
| Emission inventory | National (UK) | Vehicle exhausts | 17 and 2.6 | Factors of underestimation vehicle $NH_3$ emissions in urban and national scales compared with the 2018 UK National Atmospheric Emissions Inventory, respectively | 65 |
| Emission inventory | National (USA) | Vehicle exhausts | 7% | Fraction of vehicle $NH_3$ emissions in total $NH_3$ emissions in the USA using the error-weighted average emission ratios of $NH_3$:$CO_2$ | 66 |
| Emission inventory | Global | Transportation | 1.3 Tg N | $NH_3$ emissions from transportation in 2010 using updated emission factors, which was 3.2 times that of EDGAR | 67 |
| Emission inventory | National (China) | Household coal and biomass combustion | 0.5 Tg N | $NH_3$ emission from household coal and biomass combustion in China in 2006, using the average $NH_3$ emission factors for burning coal (1.01 mg $g^{-1}$), biomass briquette (0.95 mg $g^{-1}$), and biomass (0.96 mg $g^{-1}$) in a traditional heating stove as well as the consumption of residential coal and biomass | 68 |

| Methods | Scales | Sources | Data | Notes | Refs. |
|---|---|---|---|---|---|
| Emission inventory | Global | Biomass burning | 5.9 Tg N $yr^{-1}$ | $NH_3$ emissions from biomass burning, which account for 11% of global total $NH_3$ emissions | 69 |
| Emission inventory | Global | Biomass burning | 8.2 Tg N $yr^{-1}$ | $NH_3$ emissions from biomass burning based on the updated compilation of emission factors for 121 biomass species and published global activity data | 70 |
| Coupled human-environment N cycle model | National (China) | c-$NH_3$ | >100 kg N $ha^{-1}$ $yr^{-1}$ | The emission intensities based on coupled human-environment N cycle model are higher than that of v-$NH_3$ sources (0–80 kg N $ha^{-1}$ $yr^{-1}$) | 71 |

**Supplementary Table 3. Values of kinetic and equilibrium isotope effects ($\varepsilon_k$ and $\varepsilon_{eq}$, respectively) in the gaseous $NH_3$ conversion to particulate $NH_4^+$ (p-$NH_4^+$) in the atmosphere.** n.a. refers to 'not available'.

| **Isotope effects** | **Mean±SD values (‰)** | **Methods** | **Refs.** |
|---|---|---|---|
| Kinetic isotope fractionation ($\varepsilon_k$) | -28.0±n.a. | Calculations based on constant diffusion rates of $^{15}NH_3$ relative to $^{14}NH_3$ in the nonturbulent environment | 72 |
| | -17.7±n.a. | Calculations based on different diffusion rates of $^{15}NH_3$ relative to $^{14}NH_3$ in the turbulent environment | 1,3 |
| | -15.4±3.5 | Determined by field $\delta^{15}N$ differences between actively and passively sampled $NH_3$ in the summer | 1 |
| | -15.5±1.0 | Determined by field $\delta^{15}N$ differences between actively and passively sampled $NH_3$ in the winter | 3 |
| | -20.0±n.a. | Determined by $\delta^{15}N$ differences between $NH_3$ and p-$NH_4^+$ in controlling experiments | 73 |
| Equilibrium isotope fractionation ($\varepsilon_{eq}$) | 31.0±4.0 | Theoretical calculations at 20℃ | 74 |
| | 35.0±n.a. | Theoretical calculations at 25℃ | 75 |
| | 33.0±n.a. | Controlling experiments in a closed chamber | 73 |
| | 31.6±2.0 | Controlling experiments in a dynamic chamber with a turnover rate of 0.9 times per day | 76 |
| | 24.0±3.0 | Controlling experiments in a dynamic chamber with a turnover rate of 6.8 times per day | 76 |

**Supplementary Table 4. Simultaneously site-based $\delta^{15}N_{a\text{-}NH3}$, $\delta^{15}N_{p\text{-}NH4+}$, and $\delta^{15}N_{w\text{-}NH4+}$ used for calculating differences between the paired $\delta^{15}N_{i\text{-}NH3(a\text{-}NH3)}$, $\delta^{15}N_{i\text{-}NH3(p\text{-}NH4+)}$, or $\delta^{15}N_{i\text{-}NH3(w\text{-}NH4+)}$.** n represents replicate measurements at each site. n.a. refers to 'not available'. [a] the $\delta^{15}N_{a\text{-}NH3}$ values based on the passive samplers have been corrected by adding 15‰[1–3].

| Site, Country | Longitude | Latitude | $\delta^{15}N_{a\text{-}NH3}$ (‰) | $\delta^{15}N_{p\text{-}NH4+}$ (‰) | $\delta^{15}N_{w\text{-}NH4+}$ (‰) | Refs. |
|---|---|---|---|---|---|---|
| Suzhou, China | 120°35′ E | 31°17′ N | -16.7±3.3 (*n*=12) | 15.8±3.8 (*n*=12) | 0.5±2.8 (*n*=12) | 6 |
| Changshu, China | 120°42′ E | 31°21′ N | -18.3±6.0 (*n*=12) | 15.1±7.3 (*n*=12) | -4.9±3.1 (*n*=12) | 6 |
| Yixing, China | 119°54′ E | 31°17′ N | -17.5±6.0 (*n*=12) | 14.3±9.7 (*n*=12) | 0.5±2.8 (*n*=12) | 6 |
| Beijing, China | 116°22′ E | 39°58′ N | -15.9±5.8[a] (*n*=16) | 5.5±5.2 (*n*=26) | 0.7±4.2 (*n*=28) | 77–79 |
| Colorado, USA | 105°16′ W | 39°58′ N | -10.0±2.6 (*n*=6) | 5.6±5.5 (*n*=13) | -1.4±3.5 (*n*=11) | 80,81 |
| Providence, USA | 71°24′ W | 41°49′ N | -16.9±3.8 (*n*=12) | 4.3±4.7 (*n*=14) | n.a. | 74 |
| Yurihonjo, Japan | 140°24′ E | 39°12′ N | -16.9±8.8 (*n*=20) | 16.1±6.6 (*n*=77) | n.a. | 13 |
| Niigata, Japan | 138°51' E | 37°48' N | -8.3±3.6 (*n*=19) | 22.1±8.3 (*n*=19) | n.a. | 82 |

**Supplementary Table 5. Simultaneously seasonal observed $C_{\text{a-NH3}}$, $C_{\text{p-NH4+}}$, $\delta^{15}N_{\text{a-NH3}}$, $\delta^{15}N_{\text{p-NH4+}}$, and $\delta^{15}N_{\text{w-NH4+}}$ used for calculating $\delta^{15}N_{\text{i-NH3}}$, $^{15}\Delta_{\text{a-NH3}}$, $^{15}\Delta_{\text{p-NH4+}}$, and $^{15}\Delta_{\text{w-NH4+}}$ values (Eqs. (7–11)).** *n* represents replicate measurements at each site. n.a. refers to 'not available'. [a] the $\delta^{15}N_{\text{a-NH3}}$ based on the passive samplers has been corrected by adding 15‰[1–3].

| **Site, Country** | **Longitude** | **Latitude** | **Season** | **$\delta^{15}N_{\text{a-NH3}}$ (‰)** | **$\delta^{15}N_{\text{p-NH4+}}$ (‰)** | **$\delta^{15}N_{\text{w-NH4+}}$ (‰)** | **$C_{\text{a-NH3}}$ (μg N m$^{-3}$)** | **$C_{\text{p-NH4+}}$ (μg N m$^{-3}$)** | **Refs.** |
|---|---|---|---|---|---|---|---|---|---|
| Suzhou, China | 120°35′ E | 31°17′ N | Spring | -16.3±9.3 (*n*=3) | 29.2±4.5 (*n*=3) | 1.6±0.6 (*n*=3) | 11.5±5.5 (*n*=3) | 4.2±0.7 (*n*=3) | 6 |
| Suzhou, China | 120°35′ E | 31°17′ N | Summer | -22.7±0.4 (*n*=3) | 9.7±4.8 (*n*=3) | -0.8±1.9 (*n*=3) | 18.0±4.1 (*n*=3) | 5.8±0.8 (*n*=3) | 6 |
| Suzhou, China | 120°35′ E | 31°17′ N | Autumn | -18.3±4.7 (*n*=3) | 16.1±11.2 (*n*=3) | -2.2±2.6 (*n*=3) | 11.6±5.7 (*n*=3) | 2.0±1.4 (*n*=3) | 6 |
| Suzhou, China | 120°35′ E | 31°17′ N | Winter | -9.6±4.1 (*n*=3) | 8.2±9.9 (*n*=3) | 3.4±2.4 (*n*=3) | 4.5±1.6 (*n*=3) | 9.0±2.5 (*n*=3) | 6 |
| Changshu, China | 120°42′ E | 31°21′ N | Spring | -15.6±8.4 (*n*=3) | 23.8±4.3 (*n*=3) | -2.9±1.6 (*n*=3) | 11.4±6.1 (*n*=3) | 6.2±1.3 (*n*=3) | 6 |
| Changshu, China | 120°42′ E | 31°21′ N | Summer | -21.6±0.9 (*n*=3) | 12.1±2.5 (*n*=3) | -7.4±0.6 (*n*=3) | 19.8±4.5 (*n*=3) | 5.9±2.2 (*n*=3) | 6 |
| Changshu, China | 120°42′ E | 31°21′ N | Autumn | -19.6±3.2 (*n*=3) | 16.3±4.3 (*n*=3) | -8.0±1.4 (*n*=3) | 14.3±3.7 (*n*=3) | 4.6±2.6 (*n*=3) | 61 |
| Changshu, China | 120°42′ E | 31°21′ N | Winter | -16.2±8.8 (*n*=3) | 8.3±7.4 (*n*=3) | -1.5±0.5 (*n*=3) | 5.8±2.7 (*n*=3) | 8.9±9.2 (*n*=3) | 6 |
| Yixing, China | 119°54′ E | 31°17′ N | Spring | -17.2±7.3 (*n*=3) | 27.1±4.1 (*n*=3) | 1.6±0.6 (*n*=3) | 13.0±7.1 (*n*=3) | 3.4±1.0 (*n*=3) | 6 |
| Yixing, China | 119°54′ E | 31°17′ N | Summer | -20.9±3.0 (*n*=3) | 14.6±7.9 (*n*=3) | -0.9±1.9 (*n*=3) | 18.0±4.2 (*n*=3) | 8.6±5.9 (*n*=3) | 6 |

| **Site, Country** | **Longitude** | **Latitude** | **Season** | **$\delta^{15}N_{a\text{-}NH3}$ (‰)** | **$\delta^{15}N_{p\text{-}NH4+}$ (‰)** | **$\delta^{15}N_{w\text{-}NH4+}$ (‰)** | **$C_{a\text{-}NH3}$ (μg N $m^{-3}$)** | **$C_{p\text{-}NH4+}$ (μg N $m^{-3}$)** | **Refs.** |
|---|---|---|---|---|---|---|---|---|---|
| Yixing, China | 119°54′ E | 31°17′ N | Autumn | -18.8±2.8 (*n*=3) | 9.0±7.3 (*n*=3) | -2.2±2.5 (*n*=3) | 13.6±4.2 (*n*=3) | 4.6±4.1 (*n*=3) | 6 |
| Yixing, China | 119°54′ E | 31°17′ N | Winter | -12.9±8.6 (*n*=3) | 6.4±2.6 (*n*=3) | 3.3±2.3 (*n*=3) | 5.5±0.7 (*n*=3) | 10.1±7.3 (*n*=3) | 6 |
| Beijing, China | 116°22′ E | 39°58′ N | Summer | -13.7±4.2[a] (*n*=12) | 6.0±5.5 (*n*=17) | 1.3±4.0 (*n*=21) | 15.5±n.a. (*n*=1) | 1.2±1.1 (*n*=17) | 77–79 |
| Beijing, China | 116°22′ E | 39°58′ N | Autumn | -20.6±7.0[a] (*n*=8) | 4.6±4.7 (*n*=9) | 2.2±n.a. (*n*=1) | 10.6±n.a. (*n*=1) | 0.9±0.8 (*n*=9) | 77–79 |
| Yurihonjo, Japan | 140°24′ E | 39°12′ N | Spring | -20.8±8.4 (*n*=5) | 15.3±5.1 (*n*=9) | -6.1±2.5 (*n*=4) | 4.4±2.5 (*n*=5) | 0.4±0.3 (*n*=9) | 13,83 |
| Yurihonjo, Japan | 140°24′ E | 39°12′ N | Summer | -16.7±6.5 (*n*=5) | 18.0±7.0 (*n*=9) | -5.0±1.8 (*n*=6) | 4.5±3.3 (*n*=5) | 0.3±0.3 (*n*=9) | 13,83 |
| Yurihonjo, Japan | 140°24′ E | 39°12′ N | Autumn | -8.1±4.5 (*n*=5) | 18.8±5.3 (*n*=7) | -2.5±0.7 (*n*=6) | 1.2±0.4 (*n*=5) | 0.2±0.2 (*n*=7) | 13,83 |
| Yurihonjo, Japan | 140°24′ E | 39°12′ N | Winter | -23.9±6.7 (*n*=5) | 11.7±7.0 (*n*=8) | -4.5±0.9 (*n*=3) | 1.5±1.2 (*n*=5) | 0.3±0.2 (*n*=8) | 13,83 |
| Colorado, USA | 105°16′ W | 39°58′ N | Summer | -10.0±2.6 (*n*=6) | 5.6±5.5 (*n*=13) | -1.4±3.5 (*n*=11) | 0.2±n.a. (*n*=1) | 0.2±0.2 (*n*=5) | 80,81 |

## Supplementary References


1. Pan, Y. P. et al. Systematic low bias of passive samplers in characterizing nitrogen isotopic composition of atmospheric ammonia. *Atmos. Res.* **243**, 105018 (2020).
2. Kawashima, H., Ogata, R. & Gunji, T. Laboratory-based validation of a passive sampler for determination of the nitrogen stable isotope ratio of ammonia gas. *Atmos. Environ.* **245**, 118009 (2021).
3. Walters, W. W. et al. Characterizing the spatiotemporal nitrogen stable isotopic composition of ammonia in vehicle plumes. *Atmos. Chem. Phys.* **20**, 11551–11567 (2020).
4. Felix, J. D. et al. Examining the transport of ammonia emissions across landscapes using nitrogen isotope ratios. *Atmos. Environ.* **95**, 563–570 (2014).
5. Felix, J. D., Elliott, E. M., Gish, T. J., McConnell, L. L. & Shaw, S. L. Characterizing the isotopic composition of atmospheric ammonia emission sources using passive samplers and a combined oxidation-bacterial denitrifier approach. *Rapid Commun. Mass Sp.* **27**, 2239–2246 (2013).
6. Ti, C. P. et al. Isotopic characterization of $NH_x$-N in deposition and major emission sources. *Biogeochemistry* **138**, 85–102 (2018).
7. Ti, C. P. et al. Changes of $\delta^{15}N$ values during the volatilization process after applying urea on soil. *Environ. Pollut.* **270**, 116204 (2020).
8. Meng, D. Y. Ammonia emission and ammonia nitrogen isotope characteristics of typical agricultural and urban volatile sources. Master Thesis, Nanjing University of Information Science and Technology. (2021). (in Chinese with English abstract).
9. Chang, Y. H., Liu, X. J., Deng, C. R., Dore, A. J. & Zhuang, G. S. Source apportionment of atmospheric ammonia before, during, and after the 2014 APEC summit in Beijing using stable nitrogen isotope signatures. *Atmos. Chem. Phys.* **16**, 11635–11647 (2016).
10. Freyer, H. D. Seasonal trends of $NH_4^+$ and $NO_3^-$ nitrogen isotope composition in rain collected at Jülich, Germany. *Tellus B.* **30**, 83–92 (1978).
11. Heaton, T. H. E. $^{15}N/^{14}N$ ratios of nitrate and ammonium in rain at Pretoria, South Africa. *Atmos. Environ.* **21**, 843–852 (1987).
12. Hristov, A. N. et al. Nitrogen losses from dairy manure estimated through nitrogen mass balance and chemical markers. *J. Environ. Qual.* **38**, 2438–2448 (2009).
13. Kawashima, H. Seasonal trends of the stable nitrogen isotope ratio in particulate nitrogen compounds and their gaseous precursors in Akita, Japan. *Tellus B.* **71**, 1627846 (2019).
14. Lee, C., Hristov, A. N., Cassidy, T. & Heyler, K. Nitrogen isotope fractionation and origin of ammonia nitrogen volatilized from cattle manure in simulated storage. *Atmosphere* **2**, 256–270 (2011).
15. Shao, S. C. et al. Online characterization of a large but overlooked human excreta source of ammonia in China's urban atmosphere. *Atmos. Environ.* **230**, 117459

(2020).

16. Lv, X. M. Reactive nitrogen composition from typical sources and sources apportionment of urban atmospheric ammonia using nitrogen isotope. Master Thesis, Shandong University. (2021). (in Chinese with English abstract).

17. Song, L. L. et al. $^{15}$N natural abundance of vehicular exhaust ammonia, quantified by active sampling techniques. *Atmos. Environ.* **255**, 118430 (2021).

18. Kawashima, H. & Kurahashi, T. Inorganic ion and nitrogen isotopic compositions of atmospheric aerosols at Yurihonjo, Japan: Implications for nitrogen sources. *Atmos. Environ.* **45**, 6309–6316 (2011).

19. Xiao, H. W. et al. Enhanced biomass burning as a source of aerosol ammonium over cities in Central China in autumn. *Environ. Pollut.* **266**, 115278 (2020).

20. Emissions Database for Global Atmospheric Research (EDGAR). Data were downloaded from https://edgar.jrc.ec.europa.eu/overview.php?v=431 (accessed on 16 October 2020) (2016).

21. Holland, E. A., Braswell, B. H., Sulzman, J. & Lamarque, J. F. Nitrogen deposition onto the United States and western Europe: Synthesis of observations and models. *Ecol. Appl.* **15**, 38–57 (2005).

22. Xu, W. et al. Quantifying atmospheric nitrogen deposition through a nationwide monitoring network across China. *Atmos. Chem. Phys.* **15**, 12345–12360 (2015).

23. Li, Y. et al. Increasing importance of deposition of reduced nitrogen in the United States. *Proc. Natl Acad. Sci. USA* **113**, 5874–5879 (2016).

24. Yu, G. R. et al. Stabilization of atmospheric nitrogen deposition in China over the past decade. *Nat. Geosci.* **12**, 424–429 (2019).

25. Shephard, M. W. et al. Ammonia measurements from space with the Cross-track Infrared Sounder: characteristics and applications. *Atmos. Chem. Phys.* **20**, 2277–2302 (2020).

26. Wen, Z. et al. Changes of nitrogen deposition in China from 1980 to 2018. *Environ. Int.* **144**, 106022 (2020).

27. Statistical Review of World Energy. Data were downloaded from Https://www.bp.com/en/global/corporate/energy-economics/statistical-review-of-world-energy/downloads.html (accessed on 28 June 2020) (2020).

28. Food and Agriculture Data. Data were downloaded from Http://www.fao.org/faostat/en/#data (accessed on 26 November 2019) (2019).

29. Organisation for Economic Co-operation and Development. Data were downloaded from Https://stats.oecd.org/Index.aspx?datasetcode (accessed on 12 June 2020) (2019).

30. Regional Emission inventory in ASia version 3.2 (REAS). Data were downloaded from http://www.nies.go.jp/REAS (accessed on 31 October 2020) (2020).

31. Li, M. et al. MIX: a mosaic Asian anthropogenic emission inventory under the international collaboration framework of the MICS-Asia and HTAP. *Atmos. Chem.*

*Phys.* **17**, 935–963 (2017).

32. Kurokawa, J. et al. Emissions of air pollutants and greenhouse gases over Asian regions during 2000–2008: Regional Emission inventory in ASia (REAS) version 2. *Atmos. Chem. Phys.* **13**, 11019–11058 (2013).

33. Fagerli H. EMEP-Status Report 1/2020-Transboundary particulate matter, photo-oxidants, acidifying and eutrophying components, Norwegian Meteorological Institute (2020).

34. National Emission Ceilings Directive (NECD) emissions data viewer 1990–2018. Data were downloaded from https://www.eea.europa.eu/data-and-maps/dashboards/necd-directive-data-viewer-3 (accessed on 12 June 2020) (2020).

35. Paulot, F. et al. Ammonia emissions in the United States, European Union, and China derived by high-resolution inversion of ammonium wet deposition data: Interpretation with a new agricultural emissions inventory (MASAGE_NH3). *J. Geophy. Res. Atmos.* **119**, 4343–4364 (2014).

36. Air Pollutant Emissions Trends Data (APETD). Data were downloaded from https://www.epa.gov/air-emissions-inventories/air-pollutant-emissions-trends-data (accessed on 12 June 2020) (2020).

37. Air Pollutants Emissions Inventory (APEI) online search. Data were downloaded from https://pollution-waste.canada.ca/air-emission-inventory (accessed on 12 June 2020) (2020).

38. Kang, Y. N. et al. High-resolution ammonia emissions inventories in China from 1980 to 2012. *Atmos. Chem. Phys.* **16**, 2043–2058 (2016).

39. Huang, X. et al. A high-resolution ammonia emission inventory in China. *Global Biogeochem. Cy.* **26**, GB1030 (2012).

40. Zhang, X. M. et al. Ammonia emissions may be substantially underestimated in China. *Environ. Sci. Technol.* **51**, 12089–12096 (2017).

41. Zheng, B. et al. Trends in China's anthropogenic emissions since 2010 as the consequence of clean air actions. *Atmos. Chem. Phys.* **18**, 14095–14111 (2018).

42. Zhang, L. et al. Agricultural ammonia emissions in China: reconciling bottom-up and top-down estimates. *Atmos. Chem. Phys.* **18**, 339–355 (2018).

43. Roberts, J. M. et al. The nitrogen budget of laboratory-simulated western US wildfires during the FIREX 2016 Fire Lab study. *Atmos. Chem. Phys.* **20**, 8807–8826 (2020).

44. Suarez-Bertoa, R. & Astorga, C. Isocyanic acid and ammonia in vehicle emissions. *Transport. Res. Part D-Tr. E.* **49**, 259–270 (2016).

45. World Vehicles in Use. Data were downloaded from Https://www.statista.com (accessed on 10 November 2021) (2019).

46. Benedict, K. B. et al. Enhanced concentrations of reactive nitrogen species in wildfire smoke. *Atmos. Environ.* **148**, 8–15 (2017).

47. Kang, S., Kim, S. D. & Jeon, E. C. Emission characteristics of ammonia at

bituminous coal power plant. *Energies* **13**, 1534 (2020).

48. Wang, R. et al. Characteristics of atmospheric ammonia and its relationship with vehicle emissions in a megacity in China. *Atmos. Environ.* **182**, 97–104 (2018).

49. Kean, A. J. & Harley, R. A. On-road measurement of ammonia and other motor vehicle exhaust emissions. *Environ. Sci. Technol.* **34**, 3535–3539 (2000).

50. Chang, Y. H. et al. The importance of vehicle emissions as a source of atmospheric ammonia in the megacity of Shanghai. *Atmos. Chem. Phys.* **16**, 3577–3594 (2016).

51. Lutsch, E., Dammers, E., Conway, S. & Strong, K. Long-range transport of $NH_3$, CO, HCN, and $C_2H_6$ from the 2014 Canadian Wildfires. *Geophys. Res. Lett.* **43**, 8286–8297 (2016).

52. Perrino, C., Catrambone, M., Di Menno Di Bucchianico, A. & Allegrini, I. Gaseous ammonia in the urban area of Rome, Italy and its relationship with traffic emissions. *Atmos. Environ.* **36**, 5385–5394 (2002).

53. Wang, S. S. et al. Atmospheric ammonia and its impacts on regional air quality over the megacity of Shanghai, China. *Sci. Rep.* **5**, 15842 (2015).

54. Pan, Y. P. et al. Identifying ammonia hotspots in China using a national observation network. *Environ. Sci. Technol.* **52**, 3926–3934 (2018).

55. Decina, S. M., Hutyra, L. R. & Templer, P. H. Hotspots of nitrogen deposition in the world's urban areas: a global data synthesis. *Front. Ecol. Environ.* **18**, 92–100 (2020).

56. Sun, K., Tao, L., Miller, D. J., Khan, M. A. & Zondlo, M. A. On-road ammonia emissions characterized by mobile, open-path measurements. *Environ. Sci. Technol.* **48**, 3943–3950 (2014).

57. Elser, M. et al. High contributions of vehicular emissions to ammonia in three European cities derived from mobile measurements. *Atmos. Environ.* **175**, 210–220 (2018).

58. Lindaas, J. et al. Emissions of reactive nitrogen from western U.S. wildfires during summer 2018. *J. Geophys. Res. Atmos.* **126**, e2020JD032657 (2020).

59. Dammers, E. et al. $NH_3$ emissions from large point sources derived from CrIS and IASI satellite observations. *Atmos. Chem. Phys.* **19**, 12261–12293 (2019).

60. Van Damme, M. et al. Industrial and agricultural ammonia point sources exposed. *Nature* **564**, 99–103 (2018).

61. Clarisse, L., Van Damme, M., Clerbaux, C. & Coheur, P. F. Tracking down global $NH_3$ point sources with wind-adjusted superresolution. *Atmos. Meas. Tech.* **12**, 5457–5473 (2019).

62. Liu, L. et al. Estimating global surface ammonia concentrations inferred from satellite retrievals. *Atmos. Chem. Phys.* **19**, 12051–12066 (2019).

63. Adams, C. et al. Satellite-derived emissions of carbon monoxide, ammonia, and nitrogen dioxide from the 2016 Horse River wildfire in the Fort McMurray area. *Atmos. Chem. Phys.* **19**, 2577–2599 (2019).

64. Liu, T. Y. et al. Emission factor of ammonia ($NH_3$) from on-road vehicles in China: tunnel tests in urban Guangzhou. *Environ. Res. Lett.* **9**, 064027 (2014).

65. Farren, N. J., Davison, J., Rose, R. A., Wagner, R. L. & Carslaw, D. C. Underestimated ammonia emissions from road vehicles. *Environ. Sci. Technol.* **54**, 15689–15697 (2020).

66. Sun, K. et al. Vehicle emissions as an important urban ammonia source in the United States and China. *Environ. Sci. Technol.* **51**, 2472–2481 (2017).

67. Meng, W. J. et al. Improvement of a global high-resolution ammonia emission inventory for combustion and industrial sources with new data from the residential and transportation sectors. *Environ. Sci. Technol.* **51**, 2821–2829 (2017).

68. Li, Q. et al. Gaseous ammonia emissions from coal and biomass combustion in household stoves with different combustion efficiencies. *Environ. Sci. Technol. Lett.* **3**, 98–103 (2016).

69. Bouwman, A. F. et al. A global high-resolution emission inventory for ammonia. *Global Biogeochem. Cy.* **11**, 561–587 (1997).

70. Andreae, M. O. Emission of trace gases and aerosols from biomass burning – an updated assessment. *Atmos. Chem. Phys.* **19**, 8523–8546 (2019).

71. Fu, H., Luo, Z. & Hu, S. A temporal-spatial analysis and future trends of ammonia emissions in China. *Sci. Total Environ.* **731**, 138897 (2020).

72. Pan, Y. P. et al. Fossil fuel combustion-related emissions dominate atmospheric ammonia sources during severe haze episodes: Evidence from $^{15}N$-stable isotope in size-resolved aerosol ammonium. *Environ. Sci. Technol.* **50**, 8049–8056 (2016).

73. Heaton, T. H. E., Spiro, B. & Robertson, S. M. C. Potential canopy influences on the isotopic composition of nitrogen and sulphur in atmospheric deposition. *Oecologia* **109**, 600–607 (1997).

74. Walters, W. W., Blum, D. E. & Hastings, M. G. Selective collection of particulate ammonium for nitrogen isotopic characterization using a denuder-filter pack sampling device. *Anal. Chem.* **91**, 7586–7594 (2019).

75. Urey, H. C. The thermodynamic properties of isotopic substances. *J. Chem. Soc.* 562–581 (1947).

76. Kawashima, H. & Ono, S. Nitrogen isotope fractionation from ammonia gas to ammonium in particulate ammonium chloride. *Environ. Sci. Technol.* **53**, 10629–10635 (2019).

77. Zhang, Y., Liu, X. J., Fangmeier, A., Goulding, K. T. W. & Zhang, F. S. Nitrogen inputs and isotopes in precipitation in the North China Plain. *Atmos. Environ.* **42**, 1436–1448 (2008).

78. Wu, L. B. et al. Aerosol ammonium in the urban boundary layer in Beijing: Insights from nitrogen isotope ratios and simulations in summer 2015. *Environ. Sci. Technol. Lett.* **6**, 389–395 (2019).

79. Zhang, Y. Y. et al. Persistent nonagricultural and periodic agricultural emissions dominate sources of ammonia in urban Beijing: Evidence from $^{15}N$ stable isotope in vertical profiles. *Environ. Sci. Technol.* **54**, 102–109 (2020).

80. Moore, H. Isotopic measurement of atmospheric nitrogen compounds. *Tellus B.* **26**, 169–174 (1974).

81. Moore, H. The isotopic composition of ammonia, nitrogen dioxide and nitrate in the atmosphere. *Atmos. Environ.* **11**, 1239–1243 (1977).

82. Hayasaka, H., Fukuzaki, N., Kondo, S., Ishizuka, T. & Totsuka, T. Nitrogen isotopic ratios of gaseous ammonia and ammonium aerosols in the atmosphere. *J. Jap. Soc. Atmos. Environ.* **39**, 272–279 (2004) (in Japanese with English abstract).

83. Fukuzaki, N. & Hayasaka, H. Seasonal variations of nitrogen isotopic ratios of ammonium and nitrate in precipitations collected in the Yahiko–Kakuda Mountains Area in Niigata Prefecture, Japan. *Water Air Soil Poll.* **203**, 391–397 (2009).

**Supplementary Text 1. Publications with the $\delta^{15}N_{a\text{-}NH3}$ observations.**

84. Berner, A. H. & Felix, J. D. Investigating ammonia emissions in a coastal urban airshed using stable isotope techniques. *Sci. Total Environ.* **707**, 134952 (2020).

85. Bhattarai, N. et al. Sources of gaseous $NH_3$ in urban Beijing from parallel sampling of $NH_3$ and $NH_4^+$, their nitrogen isotope measurement and modeling. *Sci. Total Environ.* **747**, 141361 (2020).

86. Buzek, F. et al. Isotope composition of $NH_3$, $NO_x$ and $SO_2$ air pollution in the Moravia-Silesian region, Czech Republic. *Atmos. Pollut. Res.* **8**, 221–232 (2017).

87. Chang, Y. H. et al. Assessing contributions of agricultural and nonagricultural emissions to atmospheric ammonia in a Chinese megacity. *Environ. Sci. Technol.* **53**, 1822–1833 (2019).

88. Chang, Y. H., Liu, X. J., Deng, C. R., Dore, A. J. & Zhuang, G. S. Source apportionment of atmospheric ammonia before, during, and after the 2014 APEC summit in Beijing using stable nitrogen isotope signatures. *Atmos. Chem. Phys.* **16**, 11635–11647 (2016).

89. Felix, J. D., Elliott, E. M. & Gay, D. A. Spatial and temporal patterns of nitrogen isotopic composition of ammonia at U.S. ammonia monitoring network sites. *Atmos. Environ.* **150**, 434–442 (2017).

90. Hayasaka, H., Fukuzaki, N., Kondo, S., Ishizuka, T. & Totsuka, T. Nitrogen isotopic ratios of gaseous ammonia and ammonium aerosols in the atmosphere. *J. Jpn. Soc. Atmos. Environ.* **39**, 272–279 (2004). (in Japanese with English abstract)

91. Kawashima, H. & Ono, S. Nitrogen isotope fractionation from ammonia gas to ammonium in particulate ammonium chloride. *Environ. Sci. Technol.* **53**, 10629–10635 (2019).

92. Moore, H. The isotopic composition of ammonia, nitrogen dioxide and nitrate in the atmosphere. *Atmos. Environ.* **11**, 1239–1243 (1977).

93. Pan, Y. P. et al. Systematic low bias of passive samplers in characterizing nitrogen isotopic composition of atmospheric ammonia. *Atmos. Res.* **243**, 105018 (2020).

94. Savard, M. M., Cole, A., Smirnoff, A. & Vet, R. $\delta^{15}N$ values of atmospheric N species simultaneously collected using sector-based samplers distant from sources–Isotopic inheritance and fractionation. *Atmos. Environ.* **162**, 11–22 (2017).

95. Smirnoff, A., Savard, M. M., Vet, R. & Simard, M. C. Nitrogen and triple oxygen isotopes in near-road air samples using chemical conversion and thermal decomposition. *Rapid Commun. Mass Sp.* **26**, 2791–2804 (2012).

96. Stratton, J. J., Ham, J., Collett Jr, J. L., Benedict, K. & Borch, T. Assessing the efficacy of nitrogen isotopes to distinguish Colorado Front Range ammonia sources affecting Rocky Mountain National Park. *Atmos. Environ.* **215**, 116881 (2019).

97. Ti, C. P. et al. Isotopic characterization of $NH_x$-N in deposition and major emission sources. *Biogeochemistry* **138**, 85–102 (2018).

98. Walters, W. W. & Hastings, M. G. Collection of ammonia for high time-resolved nitrogen isotopic characterization utilizing an acid-coated honeycomb denuder. *Anal. Chem.* **90**, 8051–8057 (2018).

99. Walters, W. W., Blum, D. E. & Hastings, M. G. Selective collection of particulate ammonium for nitrogen isotopic characterization using a denuder-filter pack sampling device. *Anal. Chem.* **91**, 7586–7594 (2019).

100. Zhang, Y. Y. et al. Atmospheric ammonia in Beijing during the COVID-19 outbreak: Concentrations, sources, and implications. *Environ. Sci. Technol. Lett.* 32–38 (2020).

101. Zhang, Y. Y. et al. Persistent nonagricultural and periodic agricultural emissions dominate sources of ammonia in urban Beijing: Evidence from $^{15}N$ stable isotope in vertical profiles. *Environ. Sci. Technol.* **54**, 102–109 (2020).

**Supplementary Text 2. Publications with $\delta^{15}N_{w\text{-}NH4+}$ observations.**


102. Altieri, K. E., Hastings, M. G., Peters, A. J., Oleynik, S. & Sigman, D. M. Isotopic evidence for a marine ammonium source in rainwater at Bermuda. *Global Biogeochem. Cy.* **28**, 1066–1080 (2014).

103. Buzek F., Černý J. & Pačes, T. The behavior of nitrogen isotopes in acidified forest soils in the Czech Republic. *Water Air Soil Poll.* **105**, 155–164 (1998).

104. Buzek, F. et al. $^{15}N$ study of the reactivity of atmospheric nitrogen in four mountain forest catchments (Czech Republic, central Europe). *Appl. Geochem.* **116**, 104567 (2020).

105. Chen, F. J. et al. Monthly variations of the nitrogen isotope of ammonium in wet deposition in a tropical city of South China. *Aerosol Air Qual. Res.* **20**, 1062–1069 (2020).

106. Chen, Z. L. et al. Atmospheric nitrogen deposition in Yangtze River Delta: insights gained from the nitrogen content and isotopic composition of the moss Haplocladium microphyllum. *Atmos. Ocean. Sci. Lett.* **13**, 202–209 (2019).

107. Ciężka, M., Modelska, M., Górka, M., Trojanowska-Olichwer, A. & Widory, D. Chemical and isotopic interpretation of major ion compositions from precipitation: a one-year temporal monitoring study in Wrocław, SW Poland. *J. Atmos. Chem.* **73**, 61–80 (2016).

108. Cui, J. et al. A comparison of various approaches used in source apportionments for precipitation nitrogen in a mountain region of southwest China. *Environ. Pollut.* **241**, 810–820 (2018).

109. Cui, J. et al. Seasonal fluxes and sources apportionment of dissolved inorganic nitrogen wet deposition at different land-use sites in the Three Gorges reservoir area. *Ecotox. Environ. Safe.* **193**, 110344 (2020).

110. Du, F. Inorganic sulfur and nitrogen isotope variation in atmospheric precipitation at Chengdu, China. Master Thesis, Chengdu University of Technology. (2012) (in Chinese with English abstract).

111. Freyer, H. D. Seasonal trends of $NH_4^+$ and $NO_3^-$ nitrogen isotope composition in rain collected at Jülich, Germany. *Tellus B.* **30**, 83–92 (1978).

112. Fukuzaki, N. & Hayasaka, H. Seasonal variations of nitrogen isotopic ratios of ammonium and nitrate in precipitations collected in the Yahiko-Kakuda Mountains Area in Niigata Prefecture, Japan. *Water Air Soil Poll.* **203**, 391–397 (2009).

113. Gao, Y. Atmospheric nitrogen deposition to Barnegat Bay. *Atmos. Environ.* **36**, 5783–5794 (2002).

114. Garten, C. T. Nitrogen isotope composition of ammonium and nitrate in bulk precipitation and forest throughfall. *Intern. J. Environ. Anal. Chem.* **47**, 33–45 (1992).

115. Hall, S. J. et al. Convergence in nitrogen deposition and cryptic isotopic variation across urban and agricultural valleys in northern Utah. *J. Geophys. Res. Biogeo.* **121**,

2340–2355 (2016).

116.Heaton, T. H. E. $^{15}N/^{14}N$ ratios of nitrate and ammonium in rain at Pretoria, South Africa. *Atmos. Environ.* **21**, 843–852 (1987).

117.Heaton, T. H. E., Spiro, B. & Robertson, S. M. C. Potential canopy influences on the isotopic composition of nitrogen and sulphur in atmospheric deposition. *Oecologia* **109**, 600–607 (1997).

118.Hoering, T. The isotopic composition of the ammonia and the nitrate ion in rain. *Geochim. Cosmochim. Acta* **12**, 97–102 (1957).

119.Huang, S. N. et al. Seasonal pattern of ammonium $^{15}N$ natural abundance in precipitation at a rural forested site and implications for $NH_3$ source partitioning. *Environ. Pollut.* **247**, 541–549 (2019).

120.Jia, G. D. & Chen, F. J. Monthly variations in nitrogen isotopes of ammonium and nitrate in wet deposition at Guangzhou, south China. *Atmos. Environ.* **44**, 2309–2315 (2010).

121.Knapp, A. N., Hastings, M. G., Sigman, D. M., Lipschultz, F. & Galloway, J. N. The flux and isotopic composition of reduced and total nitrogen in Bermuda rain. *Mar. Chem.* **120**, 83–89 (2010).

122.Koba, K. et al. The $^{15}N$ natural abundance of the N lost from an N-saturated subtropical forest in southern China. *J. Geophys. Res. Biogeo.* **117**, G02015 (2012).

123.Lee, K. S. et al. Nitrogen isotope ratios of dissolved organic nitrogen in wet precipitation in a metropolis surrounded by agricultural areas in southern Korea. *Agr. Ecosyst. Environ.* **159**, 161–169 (2012).

124.Leng, Q. M. et al. Wet-only deposition of atmospheric inorganic nitrogen and associated isotopic characteristics in a typical mountain area, southwestern China. *Sci. Total Environ.* **616–617**, 55–63 (2018).

125.Li, X. D., Masuda, H., Koba, K. & Zeng, H. A. Nitrogen isotope study on nitrate-contaminated groundwater in the Sichuan Basin, China. *Water Air Soil Poll.* **178**, 145–156 (2007).

126.Li, Z. J. Stable isotope characteristics of nitrogen deposition and historical trajectory under conditions of different human activity. Ph.D. Thesis, University of Chinese Academy of Sciences. (2019) (in Chinese with English abstract).

127.Liu, D. W., Fang, Y. T., Tu, Y. & Pan, Y. P. Chemical method for nitrogen isotopic analysis of ammonium at natural abundance. *Anal. Chem.* **86**, 3787–3792 (2014).

128.Liu, X. Y. et al. Stable isotope analyses of precipitation nitrogen sources in Guiyang, southwestern China. *Environ. Pollut.* **230**, 486–494 (2017).

129.Moore, H. Isotopic measurement of atmospheric nitrogen compounds. *Tellus B.* **26**, 169–174 (1974).

130.Moore, H. The isotopic composition of ammonia, nitrogen dioxide and nitrate in the atmosphere. *Atmos. Environ.* **11**, 1239–1243 (1977).

131.Nanus, L., Campbell, D. H., Lehmann, C. M. B. & Mast, M. A. Spatial and temporal

variation in sources of atmospheric nitrogen deposition in the Rocky Mountains using nitrogen isotopes. *Atmos. Environ.* **176**, 110–119 (2018).

132. Proemse, B. C., Mayer, B., Fenn, M. E. & Ross, C. S. A multi-isotope approach for estimating industrial contributions to atmospheric nitrogen deposition in the Athabasca oil sands region in Alberta, Canada. *Environ. Pollut.* **182**, 80–91 (2013).

133. Russell, K. M., Galloway, J. N., Macko, S. A., Moody, J. L. & Scudlark, J. R. Sources of nitrogen in wet deposition to the Chesapeake Bay Region. *Atmos. Environ.* **32**, 2453–2465 (1998).

134. Savard, M. M., Cole, A., Smirnoff, A. & Vet, R. $\delta^{15}N$ values of atmospheric N species simultaneously collected using sector-based samplers distant from sources– Isotopic inheritance and fractionation. *Atmos. Environ.* **162**, 11–22 (2017).

135. Smirnoff, A., Savard, M. M., Vet, R. & Simard, M. C. Nitrogen and triple oxygen isotopes in near-road air samples using chemical conversion and thermal decomposition. Rapid Commun. *Mass Sp.* **26**, 2791–2804 (2012).

136. Stratton, J. J., Ham, J., Collett Jr, J. L., Benedict, K. & Borch, T. Assessing the efficacy of nitrogen isotopes to distinguish Colorado Front Range ammonia sources affecting Rocky Mountain National Park. *Atmos. Environ.* **215**, 116881 (2019).

137. Sun L. Y. Atmospheric nitrogen and phosphorus deposition in Nanjing and effects of simulated nitrogen deposition on gaseous emissions of soils. Ph.D. Thesis, Nanjing Agricultural University. (2014) (in Chinese with English abstract).

138. Ti, C. P. et al. Isotopic characterization of $NH_x$-N in deposition and major emission sources. *Biogeochemistry* **138**, 85–102 (2018).

139. Xiao, H. W., Xiao, H. Y., Long, A. M. & Liu, C. Q. $\delta^{15}N$-$NH_4^+$ variations of rainwater: Application of the Rayleigh model. *Atmos. Res.* **157**, 49–55 (2015).

140. Xiao, H. W., Xiao, H. Y., Long, A. M., Wang, Y. L. Who controls the monthly variations of $NH_4^+$ nitrogen isotope composition in precipitation? *Atmos. Environ.* **54**, 201–206 (2012).

141. Xiao, H. Y. & Liu, C. Q. Sources of nitrogen and sulfur in wet deposition at Guiyang, southwest China. *Atmos. Environ.* **36**, 5121–5130 (2002).

142. Xie, Y. X. et al. Source of nitrogen in wet deposition to a rice agroecosystem at Tai lake region. *Atmos. Environ.* **42**, 5182–5192 (2008).

143. Zhang, Y., Liu, X. J., Fangmeier, A., Goulding, K. T. W. & Zhang, F. S. Nitrogen inputs and isotopes in precipitation in the North China Plain. *Atmos. Environ.* **42**, 1436–1448 (2008).

144. Zhao, X. et al. Spatial and temporal variation of inorganic nitrogen wet deposition to the Yangtze River Delta Region, China. *Water Air Soil Poll.* **203**, 277–289 (2009).

**Supplementary Text 3. Publications with $\delta^{15}N_{p-NH4+}$ observations.**

145. Bhattarai, N. et al. Sources of gaseous $NH_3$ in urban Beijing from parallel sampling of $NH_3$ and $NH_4^+$, their nitrogen isotope measurement and modeling. *Sci. Total Environ.* **747**, 141361 (2020).

146. Hall, S. J. et al. Convergence in nitrogen deposition and cryptic isotopic variation across urban and agricultural valleys in northern Utah. *J. Geophys. Res. Biogeo.* **121**, 2340–2355 (2016).

147. Hayasaka, H., Fukuzaki, N., Kondo, S., Ishizuka, T. & Totsuka, T. Nitrogen isotopic ratios of gaseous ammonia and ammonium aerosols in the atmosphere. *J. Jpn. Soc. Atmos. Environ.* **39**, 272–279 (2004). (in Japanese with English abstract)

148. Heaton, T. H. E. $^{15}N/^{14}N$ ratios of nitrate and ammonium in rain at Pretoria, South Africa. *Atmos. Environ.* **21**, 843–852 (1987).

149. Jickells, T. D. et al. Isotopic evidence for a marine ammonia source. *Geophys. Res. Lett.* **30**, 1374 (2003).

150. Kawashima, H. & Kurahashi, T. Inorganic ion and nitrogen isotopic compositions of atmospheric aerosols at Yurihonjo, Japan: Implications for nitrogen sources. *Atmos. Environ.* **45**, 6309–6316 (2011).

151. Kundu, S., Kawamura, K. & Lee, M. Seasonal variation of the concentrations of nitrogenous species and their nitrogen isotopic ratios in aerosols at Gosan, Jeju Island: Implications for atmospheric processing and source changes of aerosols. *J. Geophys. Res. Atmos.* **115**, D20305 (2010).

152. Lim, S. et al. Fossil-driven secondary inorganic $PM_{2.5}$ enhancement in the North China Plain: Evidence from carbon and nitrogen isotopes. *Environ. Pollut.* **266**, 115163 (2020).

153. Lin, C. T., Jickells, T. D., Baker, A. R., Marca, A. & Johnson, M. T. Aerosol isotopic ammonium signatures over the remote Atlantic Ocean. *Atmos. Environ.* **133**, 165–169 (2016).

154. Moore, H. The isotopic composition of ammonia, nitrogen dioxide and nitrate in the atmosphere. *Atmos. Environ.* **11**, 1239–1243 (1977).

155. Pan, Y. P. et al. Fossil fuel combustion-related emissions dominate atmospheric ammonia sources during severe haze episodes: Evidence from $^{15}N$-stable isotope in size-resolved aerosol ammonium. *Environ. Sci. Technol.* **50**, 8049–8056 (2016).

156. Pan, Y. P. et al. Isotopic evidence for enhanced fossil fuel sources of aerosol ammonium in the urban atmosphere. *Environ. Pollut.* **238**, 942–947 (2018).

157. Pan, Y. P. et al. Source apportionment of aerosol ammonium in an ammonia-rich atmosphere: An isotopic study of summer clean and hazy days in urban Beijing. *J. Geophys. Res. Atmos.* **123**, 5681–5689 (2018).

158. Park, Y. M. et al. Characterizing isotopic compositions of TC-C, $NO_3^-$-N, and $NH_4^+$-N in $PM_{2.5}$ in South Korea: Impact of China's winter heating. *Environ. Pollut.* **233**, 735–744 (2018).

159. Proemse, B. C., Mayer, B., Chow, J. C. & Watson, J. G. Isotopic characterization of nitrate, ammonium and sulfate in stack $PM_{2.5}$ emissions in the Athabasca Oil Sands Region, Alberta, Canada. *Atmos. Environ.* **60**, 555–563 (2012).

160. Savard, M. M., Cole, A., Smirnoff, A. & Vet, R. $\delta^{15}N$ values of atmospheric N species simultaneously collected using sector-based samplers distant from sources– Isotopic inheritance and fractionation. *Atmos. Environ.* **162**, 11–22 (2017).

161. Smirnoff, A., Savard, M. M., Vet, R. & Simard, M. C. Nitrogen and triple oxygen isotopes in near-road air samples using chemical conversion and thermal decomposition. *Rapid Commun. Mass Sp.* **26**, 2791–2804 (2012).

162. Ti, C. P. et al. Isotopic characterization of $NH_x$-N in deposition and major emission sources. *Biogeochemistry* **138**, 85–102 (2018).

163. Walters, W. W., Blum, D. E. & Hastings, M. G. Selective collection of particulate ammonium for nitrogen isotopic characterization using a denuder-filter pack sampling device. *Anal. Chem.* **91**, 7586–7594 (2019).

164. Wu, C. et al. Non-agricultural sources dominate the atmospheric $NH_3$ in Xi'an, a megacity in the semi-arid region of China. *Sci. Total Environ*. **722**, 137756 (2020).

165. Wu, L. B. et al. Aerosol ammonium in the urban boundary layer in Beijing: Insights from nitrogen isotope ratios and simulations in summer 2015. *Environ. Sci. Technol. Lett.* **6**, 389–395 (2019).

166. Wu, S. P. et al. Nitrogen isotope composition of ammonium in $PM_{2.5}$ in the Xiamen, China: Impact of non-agricultural ammonia. *Environ. Sci. Pollut. Res. Int.* **26**, 25596–25608 (2019).

167. Xiang, Y. K., Cao, F., Yang, X. Y., Zhao, X. Y. & Zhang, Y. L. Hypobromite oxidation combined with hydroxylamine hydrochloride reduction method for analyzing ammonium nitrogen isotope in atmospheric samples. *Chin. J. Appl. Ecol.* **30**, 1847–1853 (2019) (in Chinese with English abstract).

168. Xiao, H. W. et al. Enhanced biomass burning as a source of aerosol ammonium over cities in Central China in autumn. *Environ. Pollut.* **266**, 115278 (2020).

169. Yeatman, S. G., Spokes, L. J., Dennis, P. F. & Jickells, T. D. Can the study of nitrogen isotopic composition in size-segregated aerosol nitrate and ammonium be used to investigate atmospheric processing mechanisms? *Atmos. Environ.* **35**, 1337–1345 (2001).

170. Yeatman, S. G., Spokes, L. J., Dennis, P. F., Jickells, T. D. Comparisons of aerosol nitrogen isotopic composition at two polluted coastal sites. *Atmos. Environ.* **35**, 1307–1320 (2001).

171. Zhang, Z. Y. et al. Fossil fuel-related emissions were the major source of $NH_3$ pollution in urban cities of northern China in the autumn of 2017. *Environ. Pollut.* **256**, 113428 (2020).

172. Zhou, Y. H. et al. Biomass burning related ammonia emissions promoted a self-amplifying loop in the urban environment in Kunming (SW China). *Atmos. Environ.* **253**, 118138 (2020).

## Supplementary Text 4. Publications with simultaneous $C_{a\text{-}NH3}$ and $C_{p\text{-}NH4+}$ observations.

### East Asia


173. Adams, P. J., Seinfeld, J. H. & Koch, D. M. Global concentrations of tropospheric sulfate, nitrate, and ammonium aerosol simulated in a general circulation model. *J. Geophys. Res. Atmos.* **104**, 13791–13823 (1999).

174. Aikawa, M., Hiraki, T. & Tamaki, M. Characteristics in concentration of chemical species in ambient air based on three-year monitoring by filter pack method. *Water Air Soil Poll.* **161**, 335–352 (2005).

175. Aikawa, M., Hiraki, T., Mukai, H. & Murano, K. Characteristic variation of concentration and chemical form in sulfur, nitrate, ammonium, and chloride species observed at urban and rural sites of Japan. *Water Air Soil Poll.* **190**, 287–297 (2008).

176. Chang, L. T. C., Tsai, J. H., Lin, J. M., Huang, Y. S. & Chiang, H. L. Particulate matter and gaseous pollutants during a tropical storm and air pollution episode in Southern Taiwan. *Atmos. Res.* **99**, 67–79 (2011).

177. Cheng, M. T., Horng, C. L. & Lin, Y. C. Characteristics of atmospheric aerosol and acidic gases from urban and forest sites in central Taiwan. *Bull. Environ. Contam. Toxicol.* **79**, 674–677 (2007).

178. Chiwa, M., Uemura, T., Otsuki, K. & Sakugawa, H. Characteristics of nitrogenous air pollutants at urban and suburban forested sites, Western Japan. *Water Air Soil Poll.* **223**, 5473–5481 (2012).

179. Cui, J., Zhou, J. & Yang, H. Atmospheric inorganic nitrogen in dry deposition to a typical red soil agro-ecosystem in southeastern China. *J. Environ. Monitor.* **12**, 1287–1294 (2010).

180. Duong Huu, H., Le Tu, T., To Thi, H., Noro, K. & Takenaka, N. Characteristics of ammonia gas and fine particulate ammonium from two distinct urban areas: Osaka, Japan, and Ho Chi Minh City, Vietnam. *Environ. Sci. Poll. Res.* **24**, 8147–8163 (2017).

181. Fu, Y. D. et al. Atmospheric dry and bulk nitrogen deposition to forest environment in the North China Plain. *Atmos. Pollut. Res.* **10**, 1636–1642 (2019).

182. Gu, M. N. et al. Concurrent collection of ammonia gas and aerosol ammonium in urban Beijing during national celebration days utilizing an acid-coated honeycomb denuder in combination with a filter system. *Environ. Sci.* **42**, 1–8 (2021) (in Chinese with English abstract).

183. Hayashi, K., Matsuda, K., Ono, K., Tokida, T. & Hasegawa, T. Amelioration of the reactive nitrogen flux calculation by a day/night separation in weekly mean air concentration measurements. *Atmos. Environ.* **79**, 462–471 (2013).

184. Kawashima H. Seasonal trends of the stable nitrogen isotope ratio in particulate nitrogen compounds and their gaseous precursors in Akita, Japan. *Tellus B*. **71**, 1627846 (2019).

185. Kim, J. S., Bais, A. L., Kang, S. H., Lee, J. & Park, K. A semi-continuous measurement of gaseous ammonia and particulate ammonium concentrations in $PM_{2.5}$ in the ambient atmosphere. *J. Atmos. Chem.* **68**, 251–263 (2011).

186. Li, K. H. et al. Atmospheric reactive nitrogen concentrations at ten sites with contrasting land use in an arid region of central Asia. *Biogeosciences* **9**, 4013–4021 (2012).

187. Liang, T. et al. High nitrogen deposition in an agricultural ecosystem of Shaanxi, China. *Environ. Sci. Pollut. Res.* **23**, 13210–13221 (2016).

188. Lin, N. H. et al. A preliminary analysis of chemical characteristics of atmospheric pollutants and their deposition budget on the Fu-Shan forest in Taiwan. *Terr. Atmos. Ocean. Sci.* **11**, 481–500 (2000).

189. Luo, X. S. et al. An evaluation of atmospheric N-r pollution and deposition in North China after the Beijing Olympics. *Atmos. Environ.* **74**, 209–216 (2013).

190. Luo, X. S. et al. Chinese coastal seas are facing heavy atmospheric nitrogen deposition. *Environ. Res. Lett.* **9**, 095007 (2014).

191. Matsumoto, K. et al. Organic and inorganic nitrogen deposition on the red pine forests at the northern foot of Mt. Fuji, Japan. *Atmos. Environ.* **237** (2020).

192. Matsumoto, M. & Okita, T. Long term measurements of atmospheric gaseous and aerosol species using an annular denuder system in Nara, Japan. *Atmos. Environ.* **32**, 1419–1425 (1998).

193. Matsumoto, R., Umezawa, N., Karaushi, M., Yonemochi, S. I. & Sakamoto, K. Comparison of ammonium deposition flux at roadside and at an agricultural area for long-term monitoring: Emission of ammonia from vehicles. *Water Air Soil Poll.* **173**, 355–371 (2006).

194. Meng, Z. Y. et al. Seasonal Variation of Ammonia and Ammonium Aerosol at a Background Station in the Yangtze River Delta Region, China. *Aerosol Air Qual. Res.* **14**, 756–766 (2014).

195. Meng, Z. Y., Lin, W. L., Zhang, R. J., Han, Z. W. & Jia, X. F. Summertime ambient ammonia and its effects on ammonium aerosol in urban Beijing, China. *Sci. Total Environ.* **579**, 1521–1530 (2017).

196. Murano, K. et al. Wet deposition of ammonium and atmospheric distribution of ammonia and particulate ammonium in Japan. *Environ. Pollut.* **102**, 321–326 (1998).

197. Pathak, R. K. & Chan, C. K. Inter-particle and gas-particle interactions in sampling artifacts of $PM_{2.5}$ in filter-based samplers. *Atmos. Environ.* **39**, 1597–1607 (2005).

198. Poor, N. et al. Nature and magnitude of atmospheric fluxes of total inorganic nitrogen and other inorganic species to the Tampa Bay watershed, FL, USA. *Water Air Soil Poll.* **170**, 267–283 (2006).

199. Quan, J. & Zhang, X. Assessing the role of ammonia in sulfur transformation and deposition in China. *Atmos. Res.* **88**, 78–88 (2008).

200. Sakurai, T., Fujita, S., Hayami, H. & Furuhashi, N. A study of atmospheric ammonia

by means of modeling analysis in the Kanto region of Japan. *Atmos. Environ.* **39**, 203–210 (2005).

201. Shen, J. L. et al. Atmospheric dry and wet nitrogen deposition on three contrasting land use types of an agricultural catchment in subtropical central China. *Atmos. Environ.* **67**, 415–424 (2013).

202. Shen, J. L. et al. Impacts of pollution controls on air quality in Beijing during the 2008 Olympic Games. *J. Environ. Qual.* **40**, 37–45 (2011).

203. Shimadera, H. et al. Sensitivity analyses of factors influencing CMAQ performance for fine particulate nitrate. *J. Air Waste Manage. Assoc.* **64**, 374–387 (2014).

204. Shon, Z. H. et al. Relationship between water-soluble ions in $PM_{2.5}$ and their precursor gases in Seoul megacity. *Atmos. Environ.* **59**, 540–550 (2012).

205. Tsai, J. H., Chang, L. P. & Chiang, H. L. Airborne pollutant characteristics in an urban, industrial and agricultural complex metroplex with high emission loading and ammonia concentration. *Sci. Total Environ.* **494**, 74–83 (2014).

206. Tsai, J. H., Chang, L. P. & Chiang, H. L. Size mass distribution of water-soluble ionic species and gas conversion to sulfate and nitrate in particulate matter in southern Taiwan. *Environ. Sci. Pollut. Res.* **20**, 4587–4602 (2013).

207. Wang, H. B. et al. Ambient concentration and dry deposition of major inorganic nitrogen species at two urban sites in Sichuan Basin, China. *Environ. Pollut.* **219**, 235–244 (2016).

208. Wang, J. F. et al. Atmospheric ammonia/ammonium-nitrogen concentrations and wet and dry deposition rates in a double rice region in subtropical China. *Environ. Sci.* **38**, 2264–2272 (2017) (in Chinese with English abstract).

209. Wang, S. B. et al. Effect of ammonia on fine-particle pH in agricultural regions of China: comparison between urban and rural sites. *Atmos. Chem. Phys.* **20**, 2719–2734 (2020).

210. Wang, W. X. et al. Gas-phase ammonia and $PM_{2.5}$ ammonium in a busy traffic area of Nanjing, China. *Environ. Sci. Pollut. Res.* **23**, 1691–1702 (2016).

211. Wei, L. F. et al. Gas-to-particle conversion of atmospheric ammonia and sampling artifacts of ammonium in spring of Beijing. *Sci. China Earth Sci.* **58**, 345–355 (2015).

212. Wen, Z. et al. Effects of reactive nitrogen gases on the aerosol formation in Beijing from late autumn to early spring. *Environ. Res. Lett.* **16**, 025005 (2021).

213. Wu, S. P. et al. Atmospheric ammonia measurements along the coastal lines of Southeastern China: Implications for inorganic nitrogen deposition to coastal waters. *Atmos. Environ.* **177**, 1–11 (2018).

214. Wu, S. P. et al. Nitrogen isotope composition of ammonium in $PM_{2.5}$ in the Xiamen, China: Impact of non-agricultural ammonia. *Environ. Sci. Pollut. Res.* **26**, 25596–25608 (2019).

215. Xu, J. et al. Importance of gas-particle partitioning of ammonia in haze formation in

the rural agricultural environment. *Atmos. Chem. Phys.* **20**, 7259–7269 (2020).

216. Xu, W., Zhang, L. & Liu, X. A database of atmospheric nitrogen concentration and deposition from the nationwide monitoring network in China. *Sci. Data* **6**, 51 (2019).

217. Yan, F. H. et al. Stabilization for the secondary species contribution to $PM_{2.5}$ in the Pearl River Delta (PRD) over the past decade, China: A meta-analysis. *Atmos. Environ.* **242**, 117817 (2020).

218. Zhang, X. et al. Pollution sources of atmospheric fine particles and secondary aerosol characteristics in Beijing. *J. Environ. Sci.* **95**, 91–98 (2020).

219. Zhang, Y. B. et al. Atmospheric deposition of inorganic nitrogen in a semi-arid grassland of Inner Mongolia, China. *J. Arid Land* **9**, 810–822 (2017).

**North America**

220. Adams, P. J., Seinfeld, J. H. & Koch, D. M. Global concentrations of tropospheric sulfate, nitrate, and ammonium aerosol simulated in a general circulation model. *J. Geophys. Res. Atmos.* **104**, 13791–13823 (1999).

221. Ammonia Monitoring Network (AMoN). Data were downloaded from https://nadp.slh.wisc.edu/networks/ammonia-monitoring-network (accessed on 12 June 2020) (2020).

222. Benedict, K. B. et al. A seasonal nitrogen deposition budget for Rocky Mountain National Park. *Ecol. Appl.* **23**, 1156–1169 (2013).

223. Bytnerowicz, A. & Fenn, M. E. Nitrogen deposition in California forests: a review. *Environ. Pollut.* **92**, 127–146 (1996).

224. Bytnerowicz, A. et al. Summer-time distribution of air pollutants in Sequoia National Park, California. *Environ. Pollut.* **118**, 187–203 (2002).

225. Clean Air Status and Trends Network (CASTNET). Data were downloaded from https://www.epa.gov/castnet (accessed on 12 June 2020) (2020).

226. Chen, L. W. A., Doddridge, B. G., Dickerson, R. R., Chow, J. C. & Henry, R. C. Origins of fine aerosol mass in the Baltimore-Washington corridor: Implications from observation, factor analysis, and ensemble air parcel back trajectories. *Atmos. Environ.* **36**, 4541–4554 (2002).

227. Cheng, B. & Wang-Li, L. Responses of secondary inorganic $PM_{2.5}$ to precursor gases in an ammonia abundant area in North Carolina. *Aerosol Air Qual. Res.* **19**, 1126–1138 (2019).

228. Cheng, B., Wang-Li, L., Classen, J., Meskhidze, N. & Bloomfield, P. Spatial and temporal variations of atmospheric chemical condition in the Southeastern US. *Atmos. Res.* **248**, 105190 (2021).

229. Edgerton, E. S., Hsu, Y. M., White, E. M., Fenn, M. E. & Landis, M. S. Ambient concentrations and total deposition of inorganic sulfur, inorganic nitrogen and base cations in the Athabasca Oil Sands Region. *Sci. Total Environ.* **706**, 134864 (2020).

230. Fahey, T. J. et al. Nitrogen deposition in and around an intensive agricultural district in central New York. *J. Environ. Qual.* **28**, 1585–1600 (1999).

231. Fenn, M. E., Geiser, L., Bachman, R., Blubaugh, T. J. & Bytnerowicz, A. Atmospheric deposition inputs and effects on lichen chemistry and indicator species in the Columbia River Gorge, USA. *Environ. Pollut.* **146**, 77–91 (2007).

232. Guo, H. et al. Fine-particle water and pH in the southeastern United States. *Atmos. Chem. Phys.* **15**, 5211–5228 (2015).

233. Herner, J. D., Aw, J., Gao, O., Chang, D. P. & Kleeman, M. J. Size and composition distribution of airborne particulate matter in northern California: I-particulate mass, carbon, and water-soluble ions. *J. Air Waste Manage. Assoc.* **55**, 30–51 (2005).

234. Langford, A. O., Fehsenfeld, F. C., Zachariassen, J. & Schimel, D. S. Gaseous ammonia fluxes and background concentrations in terrestrial ecosystems of the United States. *Global Biogeochem. Cy.* **6**, 459–483 (1992).

235. Li, Y. et al. Observations of ammonia, nitric acid, and fine particles in a rural gas production region. *Atmos. Environ.* **83**, 80–89 (2014).

236. McCurdy, T., Zelenka, M. P., Lawrence, P. M., Houston, R. M. & Burton, R. Acid aerosols in the Pittsburgh Metropolitan area. *Atmos. Environ.* **33**, 5133–5145 (1999).

237. Robarge, W. P., Walker, J. T., McCulloch, R. B. & Murray, G. Atmospheric concentrations of ammonia and ammonium at an agricultural site in the southeast United States. *Atmos. Environ.* **36**, 1661–1674 (2002).

238. Saylor, R. D., Edgerton, E. S., Hartsell, B. E., Baumann, K. & Hansen, D. A. Continuous gaseous and total ammonia measurements from the southeastern aerosol research and characterization (SEARCH) study. *Atmos. Environ.* **44**, 4994–5004 (2010).

239. Saylor, R., Myles, L., Sibble, D., Caldwell, J. & Xing, J. Recent trends in gas-phase ammonia and PM2.5 ammonium in the Southeast United States. *J. Air Waste Manage. Assoc.* **65**, 347–357 (2015).

240. Walker, J. T., Robarge, W. P., Shendrikar, A. & Kimball, H. Inorganic $PM_{2.5}$ at a US agricultural site. *Environ. Pollut.* **139**, 258–271 (2006).

241. Walker, J. T., Whitall, D. R., Robarge, W. & Paerl, H. W. Ambient ammonia and ammonium aerosol across a region of variable ammonia emission density. *Atmos. Environ.* **38**, 1235–1246 (2004).

242. Walters, W. W., Blum, D. E. & Hastings, M. G. Selective collection of particulate ammonium for nitrogen isotopic characterization using a denuder-filter pack sampling device. *Anal. Chem.* **91**, 7586–7594 (2019).

243. Weber, R. J., Guo, H., Russell, A. G. & Nenes, A. High aerosol acidity despite declining atmospheric sulfate concentrations over the past 15 years. *Nat. Geosci.* **9**, 282–285 (2016).

**Europe**

244. Adams, P. J., Seinfeld, J. H. & Koch, D. M. Global concentrations of tropospheric sulfate, nitrate, and ammonium aerosol simulated in a general circulation model. *J. Geophys. Res. Atmos.* **104**, 13791–13823 (1999).

245. Alexa, L. & Mikuska, P. Simultaneous determination of gaseous ammonia and particulate ammonium in ambient air using a cylindrical wet effluent diffusion denuder and a continuous aerosol sampler. *Anal. Chem.* **92**, 15827–15836 (2020).

246. Anatolaki, C. & Tsitouridou, R. Atmospheric deposition of nitrogen, sulfur and chloride in Thessaloniki, Greece. *Atmos. Res.* **85**, 413–428 (2007).

247. Bencs, L. et al. Mass and ionic composition of atmospheric fine particles over Belgium and their relation with gaseous air pollutants. *J. Environ. Monitor.* **10**, 1148–1157 (2008).

248. Bytnerowicz, A. et al. Chemical composition of air, soil and vegetation in forests of the Silesian Beskid Moutains, Poland. *Water Air Soil Poll.* **116**, 141–150 (1999).

249. Dammgen, U. Atmospheric nitrogen dynamics in Hesse, Germany: Creating the data base - 2. Atmospheric concentrations of ammonia, its reaction partners and products at Linden. *Landbauforschung Volkenrode* **57**, 157–170 (2007).

250. Dammgen, U. Fine particles and their constituents in Germany - Results of denuder filter measurements. *Landbauforsch. Volkenrode* **235**, 181–188 (2002).

251. Danalatos, D. & Glavas, S. Gas phase nitric acid, ammonia and related particulate matter at a Mediterranean coastal site, Patras, Greece. *Atmos. Environ.* **33**, 3417–3425 (1999).

252. Ehrnsperger, L. & Klemm, O. Source apportionment of urban ammonia and its contribution to secondary particle formation in a mid-size European city. *Aerosol Air Qual. Res.* **21**, 200404 (2021).

253. Erduran, M. S. & Tuncel, S. G. Sampling and analysis of gaseous pollutants and related particulate matter in a Mediterranean site: Antalya-Turkey. *J. Environ. Monitor.* **3**, 661–665 (2001).

254. Ferm, M. & Hellsten, S. Trends in atmospheric ammonia and particulate ammonium concentrations in Sweden and its causes. *Atmos. Environ.* **61**, 30–39 (2012).

255. Held, A., Wrzesinsky, T., Mangold, A., Gerchau, J. & Klemm, O. Atmospheric phase distribution of oxidized and reduced nitrogen at a forest ecosystem research site. *Chemosphere* **48**, 697–706 (2002).

256. Hesterberg, R. et al. Deposition of nitrogen-containing compounds to an extensively managed grassland in central Switzerland. *Environ. Pollut.* **91**, 21–34 (1996).

257. Horvath, L. & Sutton, M. A. Long-term record of ammonia and ammonium concentrations at K-puszta, Hungary. *Atmos. Environ.* **32**, 339–344 (1998).

258. Kirkitsos, P. D. & Sikiotis, D. Nitric acid, ammonia and particulate nitrates, sulphates and ammonium in the atmosphere of Athens. *Ekistics* **58**, 156–163 (1991).

259. Lee, D. S., Dollard, G. J., Derwent, R. G. & Pepler, S. Observations on gaseous and aerosols components of the atmosphere and their relationships. *Water Air Soil Poll.*

**113**, 175–202 (1999).

260.Lewandowska, A., Falkowska, L. & Beldowska, M. Ammonia and ammonium over the southern Baltic Sea. Part 2. The origin of ammonia and ammonium over two coastal stations: Gdynia and Hel. *Oceanologia* **46**, 185–200 (2004).

261.Makkonen, U. et al. Semi-continuous gas and inorganic aerosol measurements at a boreal forest site: seasonal and diurnal cycles of $NH_3$, HONO and $HNO_3$. *Boreal Environ. Res.* **19**, 311–328 (2014).

262.Marner, B. B. & Harrison, R. M. A spatially refined monitoring based study of atmospheric nitrogen deposition. *Atmos. Environ.* **38**, 5045–5056 (2004).

263.Metzger, S., Dentener, F., Krol, M., Jeuken, A. & Lelieveld, J. Gas/aerosol partitioning - 2. Global modeling results. *J. Geophys. Res. Atmos.* **107**, ACH-1-ACH 17–23 (2002).

264.Neirynck, J. et al. Fluxes of oxidised and reduced nitrogen above a mixed coniferous forest exposed to various nitrogen emission sources. *Environ. Pollut.* **149**, 31–43 (2007).

265.Pandolfi, M. et al. Summer ammonia measurements in a densely populated Mediterranean city. *Atmos. Chem. Phys.* **12**, 7557–7575 (2012).

266.Plessow, K., Spindler, G., Zimmermann, F. & Matschullat, K. Seasonal variations and interactions of N-containing gases and particles over a coniferous forest, Saxony, Germany. *Atmos. Environ.* **39**, 6995–7007 (2005).

267.Ramsay, R. et al. Surface-atmosphere exchange of inorganic water-soluble gases and associated ions in bulk aerosol above agricultural grassland pre- and postfertilisation. *Atmos. Chem. Phys.* **18**, 16953–16978 (2018).

268.Smith, A. M. et al. Ammonia sources, transport, transformation, and deposition in coastal New England during summer. *J. Geophys. Res. Atmos.* **112**, D10S08 (2007).

269.Sutton, M. A. et al. Establishing the link between ammonia emission control and measurements of reduced nitrogen concentrations and deposition. *Environ. Monit. Assess.* **82**, 149–185 (2003).

270.Sutton, M. A. et al. Fluxes of ammonia over oilseed rape - Overview of the EXAMINE experiment. *Agr. Forest Meteorol.* **105**, 327–349 (2000).

271.Tang, Y. S. et al. Acid gases and aerosol measurements in the UK (1999-2015): regional distributions and trends. *Atmos. Chem. Phys.* **18**, 16293–16324 (2018).

272.Tang, Y. S. et al. Drivers for spatial, temporal and long-term trends in atmospheric ammonia and ammonium in the UK. *Atmos. Chem. Phys.* **18**, 705–733 (2018).

273.Tang, Y. S. et al. European scale application of atmospheric reactive nitrogen measurements in a low-cost approach to infer dry deposition fluxes. *Agr. Ecosyst. Environ.* **133**, 183–195 (2009).

274. UKEAP: National Ammonia Monitoring Network. Data were downloaded from https://uk-air.defra.gov.uk/networks (accessed on 16 October 2020) (2020).

275.Van Zanten, M. C., Kruit, R. J. W., Hoogerbrugge, R., Van der Swaluw, E. & Van

Pul, W. A. J. Trends in ammonia measurements in the Netherlands over the period 1993–2014. *Atmos. Environ.* **148**, 352–360 (2017).

276. Vogt, E., Held, A. & Klemm, O. Sources and concentrations of gaseous and particulate reduced nitrogen in the city of Munster (Germany). *Atmos. Environ.* **39**, 7393–7402 (2005).

277. Wolff, V., Trebs, I., Foken, T. & Meixner, F. X. Exchange of reactive nitrogen compounds: concentrations and fluxes of total ammonium and total nitrate above a spruce canopy. *Biogeosciences* **7**, 1729–1744 (2010).

278. Zimmerling, R., Dammgen, U., Kusters, A., Grunhage, L. & Jager, H. J. Response of a grassland ecosystem to air pollutants. IV. The chemical climate: concentrations of relevant non-criteria pollutants (trace gases and aerosols). *Environ. Pollut.* **91**, 139–147 (1996).

279. Zimmermann, F., Plessow, K., Queck, R., Bernhofer, C. & Matschullat, J. Atmospheric N- and S-fluxes to a spruce forest - Comparison of inferential modeling and the throughfall method. *Atmos. Environ.* **40**, 4782–4796 (2006).
